# Synthesis and Comparison of the Photophysical Properties of Anionic Diaryl [M(C^C)(CN)_2_]^x–^ (M= Au^III^, Pt^II^) Complexes

**DOI:** 10.1021/acs.inorgchem.6c00495

**Published:** 2026-05-11

**Authors:** Iker Gil Gomez de Segura, Antonio Martín, Manfred Bochmann, Elena Lalinde, Julio Fernandez-Cestau

**Affiliations:** † Departamento de Química—Instituto de Investigación en Síntesis Química (IQUR), 16764 Universidad de La Rioja, E-26006 Logroño, Spain; ‡ Instituto de Síntesis Química y Catálisis Homogénea (ISQCH) CSIC, Universidad de Zaragoza, C/Pedro Cerbuna 12, Zaragoza 50009, Spain; § School of Chemistry, 6106 University of East Anglia, Norwich Research Park, Norwich NR4 7TJ, U.K.

## Abstract

The d^8^ complexes A­[Au­(C^C)­(CN)_2_] and A_2_[Pt­(ĈC)­(CN)_2_] (where C^C = 4,4′-di-tert-butylbiphenyl-2,2′-diyl and A^+^ = NBu_4_^+^, K^+^) were synthesized from [Au­(C^C)­Cl]_2_ and [Pt­(C^C)­COD], which were themselves obtained from Sn­(C^C)^n^Bu_2_. These complexes are bright photoemitters but exhibit remarkable differences in the origin of their photoluminescence. The tin complex Sn­(C^C)^n^Bu_2_ displays blue/white photoluminescence originating from an admixture of transitions. In contrast, the gold complexes exhibit green, long-lived phosphorescence (lifetimes up to 100 μs), which theoretical calculations attribute to ^3^IL­(C^C) transitions. Although the platinum complexes show similar absorption and emission energies, theoretical calculations indicate an admixture with ^3^MLCT character in their emissive states. This is evidenced by (i) luminescence lifetimes up to 1 order of magnitude shorter than those of the gold complexes under similar conditions; (ii) a greater contribution from fluorescence with respect to phosphorescence in solution, and (iii) reduced susceptibility to ^3^O_2_ quenching, a consequence of the shorter triplet-state lifetime. The combination of water solubility, efficient ISC, and a long-lived triplet state with high sensitivity to dissolved O_2_ endows K­[Au­(C^C)­(CN)_2_] with excellent catalytic activity in the photo-oxidation of *p*-bromothioanisole. This result underscores the potential of this class of Au­(III) salts for photocatalysis in green solvents.

## Graphical abstract

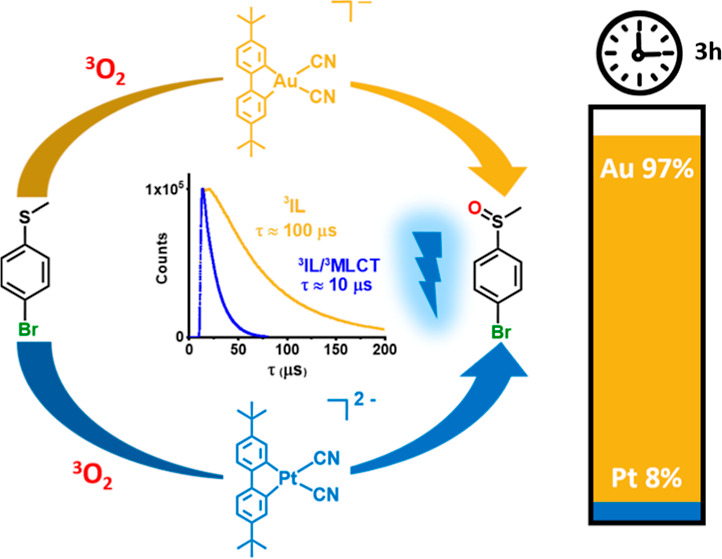

## Introduction

Square-planar d^8^ platinum­(II) complexes exhibit impressive photophysical properties, making them attractive for optoelectronic and photonic applications.^1–5^ Their isoelectronic gold­(III) analogues have more recently emerged as excellent alternatives.^6,7^ In both families, the introduction of strong ligand-field splitting ligands is essential to favor emissive pathways by preventing the population of metal-centered (MC) d-states, which promote nonradiative deactivation.

A highly prolific strategy to achieve this consists in the introduction, via cyclometalation, of 2-arylpyridine (C^N) ligands as chromophores.^2,7–9^ This approach typically generates emissive states with mixed triplet ligand-centered (^3^LC) and metal-to-ligand charge transfer (^3^MLCT) character. The photophysical properties can be readily modulated by the strategic introduction of substituents on the chelating ligand or by employing different heterocycles in place of pyridine.^10,11^ To further enhance emission, the remaining coordination sites are often occupied by strong σ-donor ligands, such as alkyl, aryl, alkynyl, N-heterocyclic carbenes, or thiolates.^12,13^

Despite their isoelectronic relationship, Pt­(II) and Au­(III) display significant chemical differences. For instance, the chemistry of Pt­(II) olefin complexes is a centenary topic, while π-complexes of Au­(III) have been remarkably elusive and were only isolated a few years ago.^14–16^ In our search for alternative platforms to stabilize Au­(III) alkene and alkyne complexes, we employed the tert-butyl-substituted 2,2′-biphenyl-diyl ligand originally developed by Mohr and co-workers.^17^ Beyond their stability, complexes based on this motif often show intense photoluminescence, and several novel series of emissive biphenyl-derived C^C gold­(III) complexes have been reported.^18–20^

In parallel, emissive biphenyl complexes are also known in platinum­(II) chemistry.^21^ Notable examples are the class of highly luminescent anionic Pt­(II)-biaryl diyl complexes with cyanide ligands reported by Kato et al. in 2020.^22^

The cyanide ligand is, in fact, widely employed in the design of emissive materials. First, it is a simple, accessible ligand that induces a high ligand-field splitting.^23,24^ Second, its presence enables the formation of supramolecular assemblies through secondary terminal N­(cyano)···M interactions with cations, leading to rich photophysical behavior.^23,25^ This concept dates back to 1969, when Krogmann’s reported his famous tetracyanoplatinate salt^26^ and has been expanded in many subsequent studies. The work of Leznoff on both platinum and gold systems has been particularly instrumental in demonstrating the profound influence of the cation on the material’s photophysics.^27,28^ Remarkably, by using a Lu^3+^ cation, this group recently reported the structure of [Lu­(bipyO_2_)_4_]­{[Au­(CN)_4_]_3_}, which exhibits typically elusive aurophilic Au­(III)···Au­(III) interactions.^29^

Motivated by the aforementioned differences between Au­(III) and Pt­(II) and the potential of cyanide ligands, we report herein the synthesis and characterization of the complexes A_n_[M­(C^C)­(CN)_2_] (A^+^ = NBu_4_^+^, K^+^; *n* = 1, M = Au; *n* = 2, M = Pt; C^C = 4,4′-di-tert-butylbiphenyl-2,2′-diyl). The photophysical properties of these complexes were investigated, revealing significant intrinsic differences in the origin of their emissions depending on the metal center. The observed strong quenching in aerated solutions was rationalized, and, based on this, we evaluated the efficacy of the most promising complex as a photosensitizer in the oxidation of *p*-bromothioanisole in MeOH-H_2_O solution.

## Results and Discussion

### Synthesis and Spectroscopic Characterization

The synthesis of the highly insoluble chloride-bridged dimer [Au­(C^C)­Cl]_2_ **2** was adapted from the method of Mohr et al.,^17^ which itself was based on the work of Usón for analogous biphenyl complexes.^30^ The successful strategy employs the dibenzostannole Sn­(C^C)^n^Bu_2_ 1, prepared from an in situ synthesized dilithio biaryl reactant **A**, as a soft diarylating agent with one equivalent of HAuCl_4_.^17^ All our attempts to perform direct metalation of gold using intermediate **A** resulted in the reduction of the gold source, evidenced by the formation of purple gold nanoparticles and the recovery of 4,4′-di-tert-butylbiphenyl.

In contrast, the related complex [Pt­(C^C)­(COD)] (**4**) was in fact initially generated, as an orange solid in a ca. 40% yield, by reacting the dilithio biaryl **A** with PtCl_2_COD at a very low temperature (Method A). However, an alternative synthesis is achieved via transmetalation from tin; refluxing PtCl_2_(COD) with Sn­(C^C)^n^Bu_2_ **1** in CHCl_3_ for 24 h, in similar yield.

Subsequent ligand substitutions yielded the target dicyano complexes. Treatment of gold dimer **2** with two equivalents of NBu_4_CN or KCN produced NBu_4_[Au­(C^C)­(CN)_2_] **3** and K­[Au­(C^C)­(CN)_2_] **3** ^**K**^, respectively. Similarly, displacement of the COD ligand from platinum complex **4** with NBu_4_CN or KCN afforded the dianionic complexes (NBu_4_)_2_[Pt­(C^C)­(CN)_2_] **5** and K_2_[Pt­(C^C)­(CN)_2_] **5** ^**K**^.

Beyond confirming the identity of the complexes, the spectroscopic characterization revealed several noteworthy features. The ^1^H NMR spectra, recorded in acetone-d_6_, are distinguished by an unshielded resonance for the H^2^ proton of the C^C ligand (see Scheme 1 for numbering), which exhibits ^195^Pt satellites for the platinum complexes **4**, **5**, and **5** ^**K**^.

**Scheme 1:**
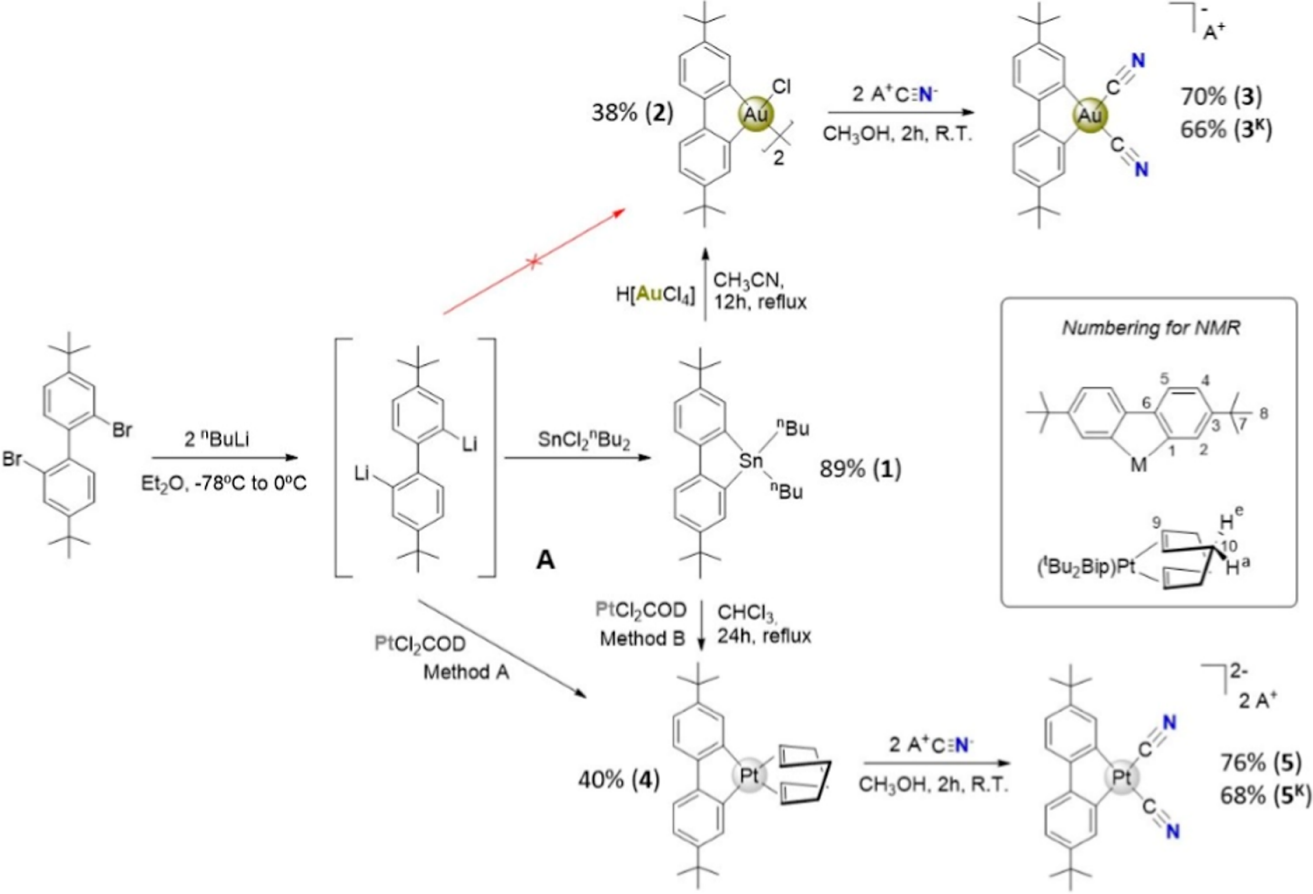
Synthesis of Complexes

The most diagnostic signals in the ^13^C­{^1^H} NMR spectra correspond to the cyanide carbons, and to further confirm the identity of the C­(CN) signals, the ^13^C-labeled complexes K­[Au­(C^C)­(^13^C≡N)_2_] 3^K^* and K_2_[Pt­(C^C)­(^13^C≡N)_2_] 5^K^* were prepared on a small scale (see experimental section), and their ^13^C­{^1^H} NMR spectra were recorded in acetone-d^6^ (Figure 1a). In the gold series, these signals appear at very similar shifts, 141.0 (**3**) and 141.1 (3^K^*). In contrast, in the platinum series a notable difference is observed (δ 146.2 ^1^J_Pt–C_ = 817 Hz for **5** vs δ 150.3 ppm with a ^1^J_Pt–C_ = 802 Hz for 5^K^*).

**Figure 1:**
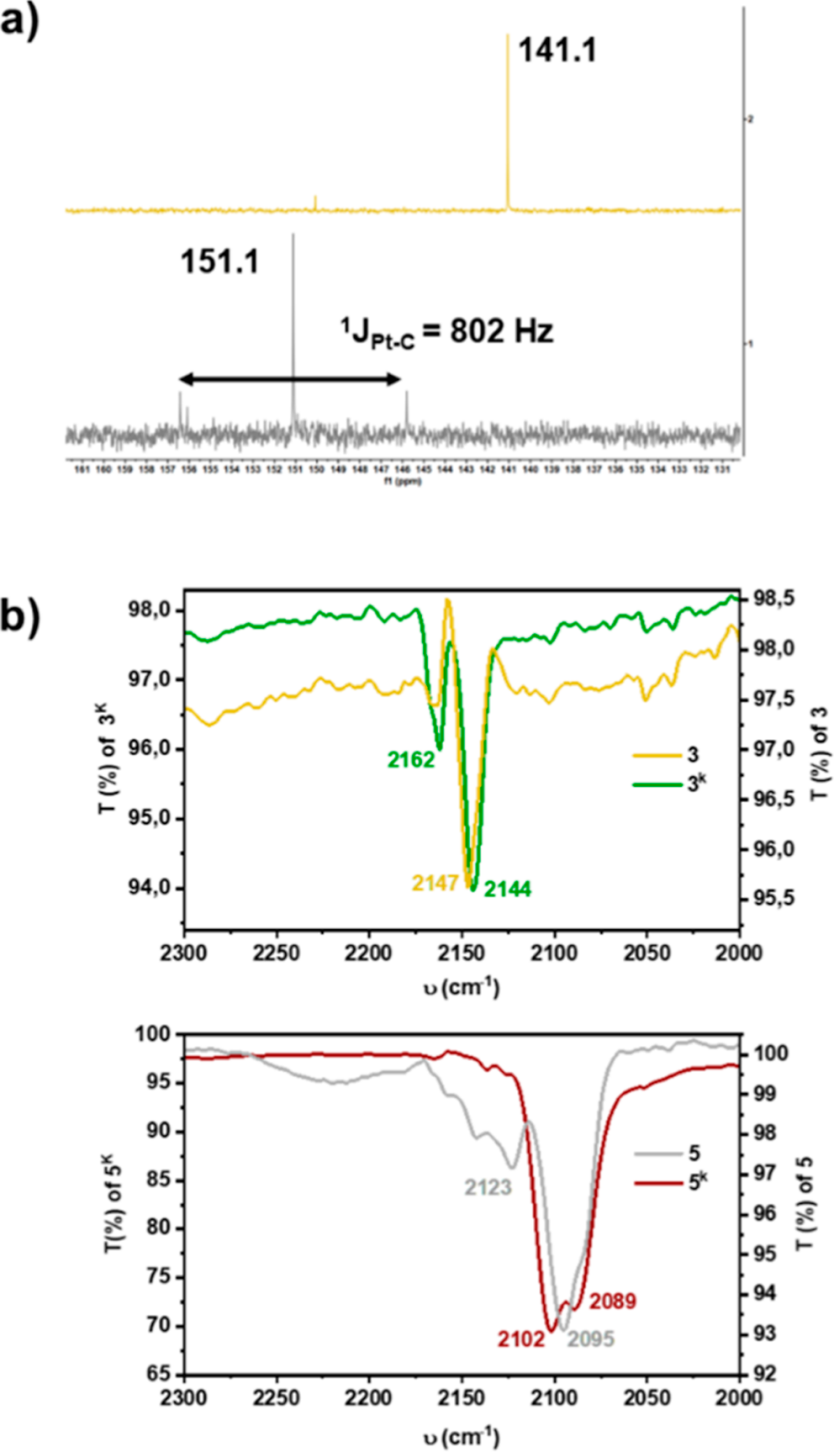
(a) ^13^C­{^1^H} NMR spectra of K­[Au­(C^C)­(^13^CN)_2_] (3^K^*) and K_2_[Pt­(C^C)­(^13^CN)_2_] (5^K^*). (b) IR spectra of complexes **3**, **3** ^**K**^, **5**, and **5** ^**K**^.

This chemical shift mobility and variations in coupling constants between **5** and 5^K^* are consistently explained by the hypothetical presence of secondary C≡N···K^+^ interactions in solution. These interactions presumably decrease the electron density on the cyanide carbon, and for the dianionic complex **5** ^**K**^, it is very possible that the 2:1 electrolyte stoichiometry particularly favors the formation of such ion pairs with K^+^ cations.

This interpretation is further supported by IR spectroscopy (Figure 1b). The ν­(C≡N) stretching frequencies for the platinum complexes **5** and **5** ^**K**^ appear at lower energies than those of their gold analogues **3** and **3** ^**K**^, consistent with greater π-back-donation from Pt­(II) compared to Au­(III). The pronounced shift observed between the NBu_4_^+^ and K^+^ salts in the platinum series is attributed to C≡N···K^+^ interactions in the solid state, a conclusion suggested by X-ray crystallographic data.

**X-ray diffraction studies:**

Crystals of 1, **3**, **3** ^**K**^, and **4** were used for single-crystal diffraction experiments. To better understand the photophysical properties of the tin complex, we isolated the white crude material as bright, colorless blocks. We took the opportunity to resolve the X-ray structure of the complex that reveals the expected tetrahedral coordination environment for the Sn­(IV) center (see Supporting Information, Section S2). The coordinates from this structure were subsequently used as inputs for theoretical calculations, as discussed later.

The molecular structures of the gold and platinum complexes **3** and **4** are shown in Figure 2, with selected bond lengths and angles provided in the caption. In both complexes, the metric parameters are consistent with a distorted square-planar geometry for a d^8^ metal ion, accommodating the constraints of the C^C chelate. The Au–C­(cyanide) bond distances in **3** (2.049(9)–2.061(8) Å) are notably longer than those reported for cyanide ligands trans-to a pyridine nitrogen in neutral cyclometalated Au­(C^N)­(CN)_2_ complexes (1.966(5)–1.981(5) Å). However, they are slightly shorter than Au–C­(cyanide) bonds trans to a phenyl ring in the same series (2.066(3)–2.078(5) Å).^24^ This trend reflects the anionic character of complex **3**, which is further evidenced by its Au–C­(biaryl) distances (2.045(8)–2.049­(8 Å) being longer than the corresponding Au–C­(aryl) bonds in neutral Au­(C^N)­(CN)_2_ complexes (2.020(5)–2.041(4) Å).

**Figure 2:**
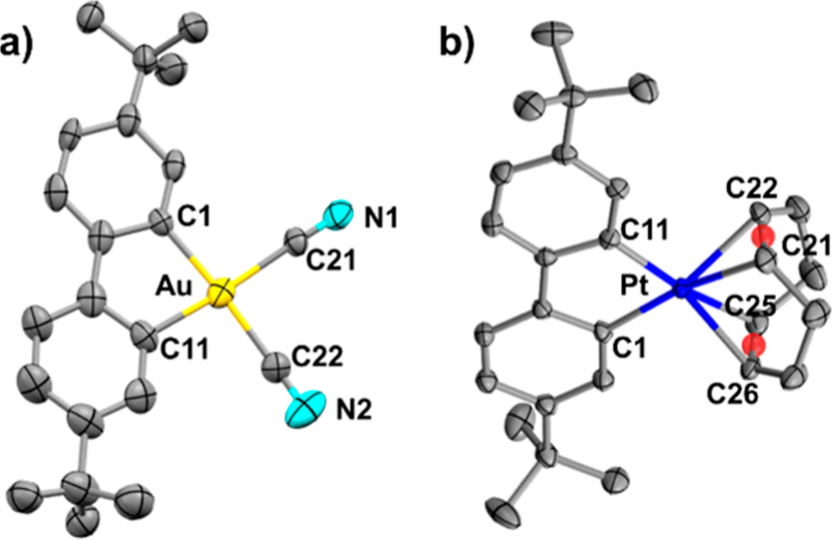
Molecular structures [selected bond distances (Å) and angles (°)]: (a) **3**: Au–C1 2.049(8), Au–C11 2.045(8), Au–C21 2.061(8), C21–N1 1.13(1), Au–C22 2.049(9), C22–N2 1.13(1), C1–Au–C11 81.8(3), C1–Au–C21 94.5(3), C11–Au–C22 95.0(4), C21–Au–C22 88.8(3), C1–Au–C22 175.4(3), C11–Au–C21 174.8(3), Au–C21–N1 177.1(8), Au–C22–N2 177.5(9). (b) **4**: Pt–C1 2.035(2), Pt–C11 2.035(3), Pt-centroid­(C21C22) 2.156, Pt-centroid­(C25C26) 2.138, C1–Pt–C11 81.2(1), C1–Pt-centroid­(C25C26) 96.47, C11–Pt-centroid­(C21C22) 97.54, centroid­(C21C22)-Pt-centroid­(C25C26) 84.95, C1–Pt-centroid­(C21C22) 176.57, and C11–Pt-centroid­(C25C26) 176.60.

In the platinum complex **4**, the distances from the Pt­(II) center to the centroids of the COD olefin bonds (2.138, 2.156 Å) are comparable to those found in similar complexes (2.12–2.18 Å).^22^

Colorless blocks of potassium salt **3** ^**K**^ were grown by layering an acetone solution with diisopropyl ether. Although the obtained crystals were twinned, resulting in a model of insufficient quality for a detailed discussion of the bond parameters, the structure unequivocally confirms the molecular connectivity. Different views of the molecular packing are shown in Figure 3 from which key conclusions can be drawn.

**Figure 3:**
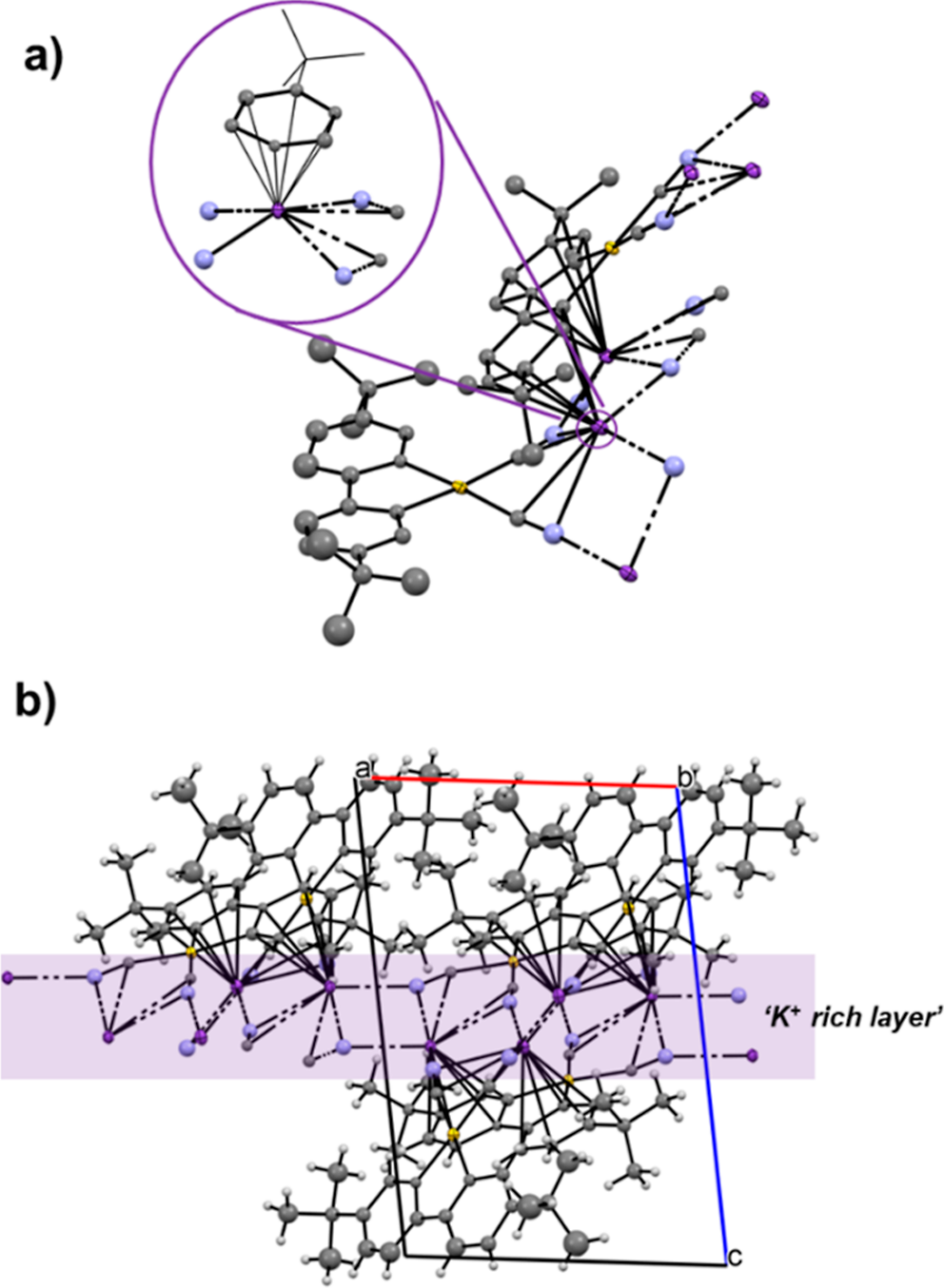
(a) Asymmetric unit and coordination environment of the K^+^ cations (inset) in the X-ray structure of **3** ^**K**^. (b) View of the packing along the *ac* face. (The X-ray data do not allow an accurate analysis of bond distances and angles).

Unlike many cyanide complexes with K^+^ that crystallize with solvent molecules (e.g., water and alcohols), the packing in **3** ^**K**^ is exceptionally tight. The K^+^ ions reside in a coordination sphere comprised of (i) two direct N­(cyano)···K^+^ interactions with cyanide groups from two different molecules, (ii) two π-interactions with the cyanide ligands of the same Au­(III) anion, and (iii) one π-(η^6^-arene) interaction with the C^C ligand of an adjacent [Au­(C^C)­(CN)_2_]^−^ anion (Figure 3a). Furthermore, as shown in Figures 3b and S20, these interactions propagate to form K^+^-rich layers perpendicular to the *c* crystallographic axis. This dense, solvent-free packing is structurally significant and has direct implications for the photophysical properties of the complexes, as discussed in the following section.

### Photophysical Properties and Theoretical Calculations

Detailed photophysical measurement procedures are provided in the Supporting Information (Section S3), with key data summarized in Table 1. To gain deeper insight into these properties, theoretical calculations were performed in the gas phase and in CH_2_Cl_2_ solution using the Gaussian 16 package (for details on functionals and basis sets, see Supporting Information, Section S4).

**Table 1:** Photophysical Properties of the Complexes

		λ_abs_/nm (ε/mM^–1^ cm^–1^)	λ_*e*m_/nm (λ_ex_/nm)	τ/μs [λ_ex_ ^a^ ^,^ ^b^ ^,^ ^c^-λ_em_/nm]	ϕ/% (λ_ex_/nm)^d^
1	Crystals		346, 354, 474, 494_max_, 530sh (290)	0.003 [375^a^-475]	
	Powder		342, 354, 372, 436, 462, 490_max_ (290) 484 (360)//435, 462, 490_max_ (400)//666, 730_max_ (400 delay 10 μs)	0.004 (37%), 0.015 (63%) [375^a^-490]	
	CH_2_Cl_2_ ^e^	234 (32.20), 287 (25.90), ∼ 400*br* (2.6)	605, 650_max_, 694sh (334, 346, 364, 385, 440)	11.9 (60%), 5.1 (40%) [440^b^-660]	
	Me-THF^e^	215 (28.38), 233 (19.87), 241sh (15.52), 288 (15.33), 318 (0.86)	420_max_, 590, 645 (360)		
**2**	Solid		488, 524, 560_max_, 600 (385)		
**3**	Crystals		488, 525_max_, 562, 601*sh* (350)	106.4 [350^b^-525]	9.2 (350)
	PMMA^f^		488, 524_max_, 560, 604sh (350)	51.8 (67%), 80.6 (33%) [360^b^-524]	20 (350)
	CH_2_Cl_2_ ^e^	229 (22.02), 251 (24.07), 259 (28.77), 272sh (4.45), 286 (3.94), 305 (5.19), 317 (6.36), 339 (1.95)	402, 492, 528_max_, 564, 608 (270, 318, 342)	0.0028 [295^c^-405] 64.1 [318^b^-528]	
	Me-THF^e^	201 (16.89), 218 (12.00), 250 (15.85), 258 (19.27), 287 (1.22), 305 (3.04), 317 (3.85), 343 (0.82)	369, 383, 402, 488, 523_max_, 561, 603 (318, 344)	1.8 (10%), 11.2 (90%) [318^b^-523]	
**3** ^K^	solid		492, 528_max_, 564, 610sh (360) 564_max_ (420)	55.6 (63%), 76.6 (37%) [360^b^-528] 11.4 [420^b^-564]^g^	7.4 (350)
	MeOH/H_2_O (1:9)^e^	196 (48.40), 219 (25.66), 249 (29.51), 258 (38.45), 285 (4.34), 304 (6.20), 316 (8.08), 346 (2.36)	492, 530, 566, 612sh (344)	37.0 [340^b^-530]	
**4**	Solid		550, 590_max_, 630, 680sh (394)	14.1 [394^b^-590]	<1 (360)
	PMMA^f^		554, 590_max_, 630, 690sh (394)	11.3 [395^b^-590]	1.3 (380)
	CH_2_Cl_2_ ^e^	232 (26.38), 274 (46.98), 292 (44.26), 308sh (16.90), 363 (1.99), 391 (1.19)	561, 595_max_, 641 (360, 390)	2.2 (7%), 13.8 (93%) [390^b^-595]	
**5**	Oil		500, 537_max_, 577, 620sh (350)	11.0 (75%), 3.4 (25%) [340^b^-535]	14 (350)
	PMMA^b^		462_weak_, 500, 538_max_, 570, 620sh (342)	13.8 [360^b^-538]	38 (342)
	CH_2_Cl_2_^e^	196, 202, 222, 267, 288, 307, 321, 340, 362	506, 542_max_, 579, 624sh (334, 365, 380)	11.7 [334^b^-542]	
	Me-THF^e^	202, 213, 237, 267, 289, 307, 332, 339, 359, 374	405_max_, 416sh, 504, 542, 578, 628sh (338)	0.0037 [295^c^-405]	
**5** ^**K**^	Solid		505, 540_max_, 580, 622sh (340) 514sh, 576_max_ (452)	13.8 [360^b^-542]	2.3 (340)
	MeOH/H_2_O (1:9)^e^	330 (63.41), 340 (74.25), 352 (65.81), 377(28.76)	387, 507, 544_max_, 581, 627sh (330, 360, 400)	2.5 (10%), 15.8 (90%) [330^b^-544]	
					

a TCSP nanoLED with λ 375 nm and pulse lengths of 50 ps.

b μF2 pulse lamp (power: 100 W, fuse: 3.15 Amp A/S).

c TCSP nanoLED with λ 295 nm and pulse lengths of 50 ps.

d Hamamatsu absolute PL quantum yield measurement system.

e 5 × 10^–4^ M. Deoxygenated.

f The PMMA film (1 wt %) was kept under N_2_ for several days before measurement.

g The decay was fitted to three components, keeping two fixed to the components found for the main band.

#### Photophysics of the Tin Complex (1)

Although compound **1** has been known for some time, its photophysical properties have remained unexplored, which is surprising given the rich luminescence of heterofluorenes,^31–34^ and their application in PhOLEDs (phosphorescent organic light-emitting diodes).^35^ The crude solid of 1 exhibits an intense whitish-blue emission under a 365 nm hand lamp. As shown in Figure 4a, excitation at 290 nm produces a complex emission profile consisting of a high-energy component (346, 354 nm), associated with an excitation maximum at 320 nm, and an intense low-energy band (λ_max_ = 490 nm) associated with a broad excitation band (360–425 nm).

**Figure 4:**
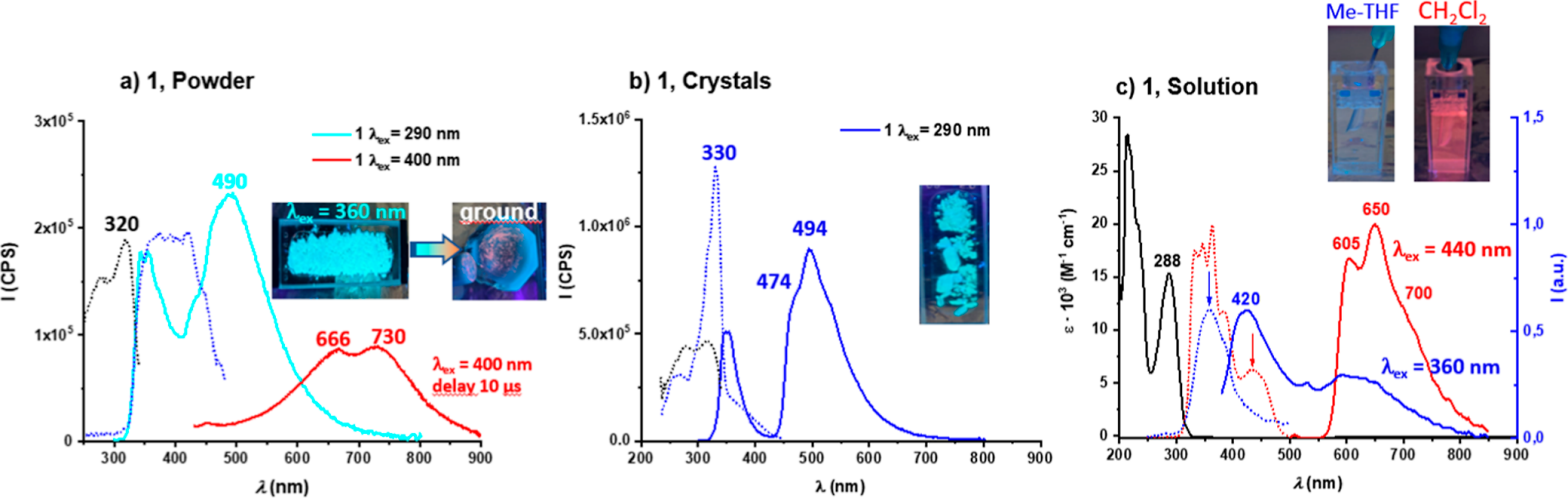
(a) PL spectra (solid lines for emission and dotted lines for excitation) of complex 1 in the solid state and pictures of the crude and the ground solid under UV (365 nm) light. (b) PL spectra of crystals of complex 1 and the picture of the crystals under UV light. (c) UV–vis absorption spectra of complex **1** in Me-THF (5 × 10^–4^ M) (black line). PL spectra of complex **1** in Me-THF (5 × 10^–4^ M) (blue lines) and CH_2_Cl_2_ (5 × 10^–4^ M) (red lines) and pictures of the solutions under UV light. CPS (counts per second).

Excitation within this low-energy range reveals the 490 nm band, accompanied by long tails extending into the deep red region with variable relative intensities depending on the particular batch of sample. Time-resolved measurements at 490 nm show biexponential decay with lifetimes of 4 and 15 ns, consistent with prompt singlet fluorescence. However, the delayed emission spectrum (measured with a 10 μs gate after excitation) of the crude solid reveals a low-energy profile with peaks at 666 and 730 nm. This suggests that the solid-state emission of 1 originates from a mixture of high-energy ^1^IL­(C^C) blue fluorescence (the dominant component) and lower-energy emission from either ^3^IL­(C^C) phosphorescence or excimer formation. The excimer assignment is further supported by the appearance of a red emission upon grinding the solid, likely due to the formation of aggregates through close π···π interactions. Unfortunately, we were unable to measure the steady-state emission or lifetime of this ground solid, as the signal was too low.

As mentioned in the X-ray section, large colorless crystals were grown from the crude powder. Their photoluminescence spectrum upon 290 nm excitation (Figure 4b) is similar to that of the powder but with better resolution: the high-energy (346, 354 nm) and low-energy bands (474, 494, 530 nm) are more distinct, and the vibrational structure of the latter is clearly visible (see Figure 4b).

Theoretical calculations confirm that the frontier orbitals are localized on the C^C fragment. While low-energy phosphorescence in heterofluorenes has been linked to heavy-atom-enhanced intersystem crossing (ISC),^31,34^ TADF has been reported in Sn complexes due to intramolecular heavy-atom effects.^36^ The precise origin of all emission bands in 1 remains to be unequivocally assigned, but we plan to come back to this kind of system in the future.

In solution (Figure 4c), the lowest-energy UV–vis absorption band of **1** in CH_2_Cl_2_ and Me-THF is located at 288 nm and is assigned to ^1^IL­(C^C) transitions. Specifically, this band is attributed to the S_1_ state (calculated λ = 273 nm, 86% HOMO→LUMO) and the S_2_ state (calculated λ = 263 nm, 74% HOMO→LUMO+1). Additional low-energy features with low absorptivity are present, particularly in CH_2_Cl_2_. These apparent residual absorptions are highly relevant, as they are associated with the luminescence in fluid media. In deoxygenated Me-THF, excitation at 360 nm produces photoluminescence dominated by a blue component (420 nm), with an additional low-energy band. In CH_2_Cl_2_, the low-energy component (605, 650, 700 nm) is dominant and visible to the naked eye. The emission decay fits a biexponential model with microsecond-range lifetimes (11.9 μs (60%) and 5.1 μs (40%)).

#### Photophysics of the Gold and Platinum Complexes

Like for the tin complex, the lowest-lying energy absorption bands of the gold and platinum complexes **3**-**5** are dominated by the biphenyl chromophore but with interesting particularities that start to show the different character of each family. Complex **3** shows in CH_2_Cl_2_ and Me-THF similar absorption bands (**3** 339, 317 nm; **3** ^**K**^ 346, 316 nm) ascribed to transitions with marked C^C character (see Table 1 and Figure S22). These bands are notably red-shifted in relation to those observed in the tin complex **1** (CH_2_Cl_2_ 318, 288 nm), reflecting the influence of the metal center. According to theoretical calculations (see Section S4 and Figure 5), the introduction of the gold center has two clear effects. The first is the rise in energy of the C^C-based HOMO and slight stabilization of the LUMO, which explains the red-shift of the lowest energy absorption band of **3** with respect to **1**. The second is the participation of the gold (15%) and the cyanides (8%) orbitals in the LUMO, revealing some ligand-to-cyanide-metal charge transfer contribution (LML’CT) to the low-lying absorption feature in both complexes (**3** 339; **3** ^**K**^ 349 nm). The charge transfer character of the HOMO–LUMO transition is evident but different in nature for complex **5**. Thus, in this region, the related dianionic Pt complexes show a band at ca. 340 nm but with a low energy tail extending to 374 nm (Figures S22, Table 1). As can be seen in Figure 5, in this dianion, the HOMO, mainly contributed from the C^C ligand with a remarkable Pt contribution (30%), rises notably in energy with respect to **3**, a fact that should be attributed not only to the higher Pt contribution but also to the overall charge of the molecule. The LUMO orbital has a similar composition to **3**, and therefore, the lowest energy transition has ^1^IL/^1^MLCT character. These calculations also support the slight red shift of the lowest energy absorption of **5** with respect to **3**, given the smaller HOMO–LUMO gap. Finally, with the support of theoretical calculations, the lowest energy absorption of complex **4** (391 nm) can be attributed to a ^1^LL’CT (C^C→COD) transition (Figure 5).

**Figure 5:**
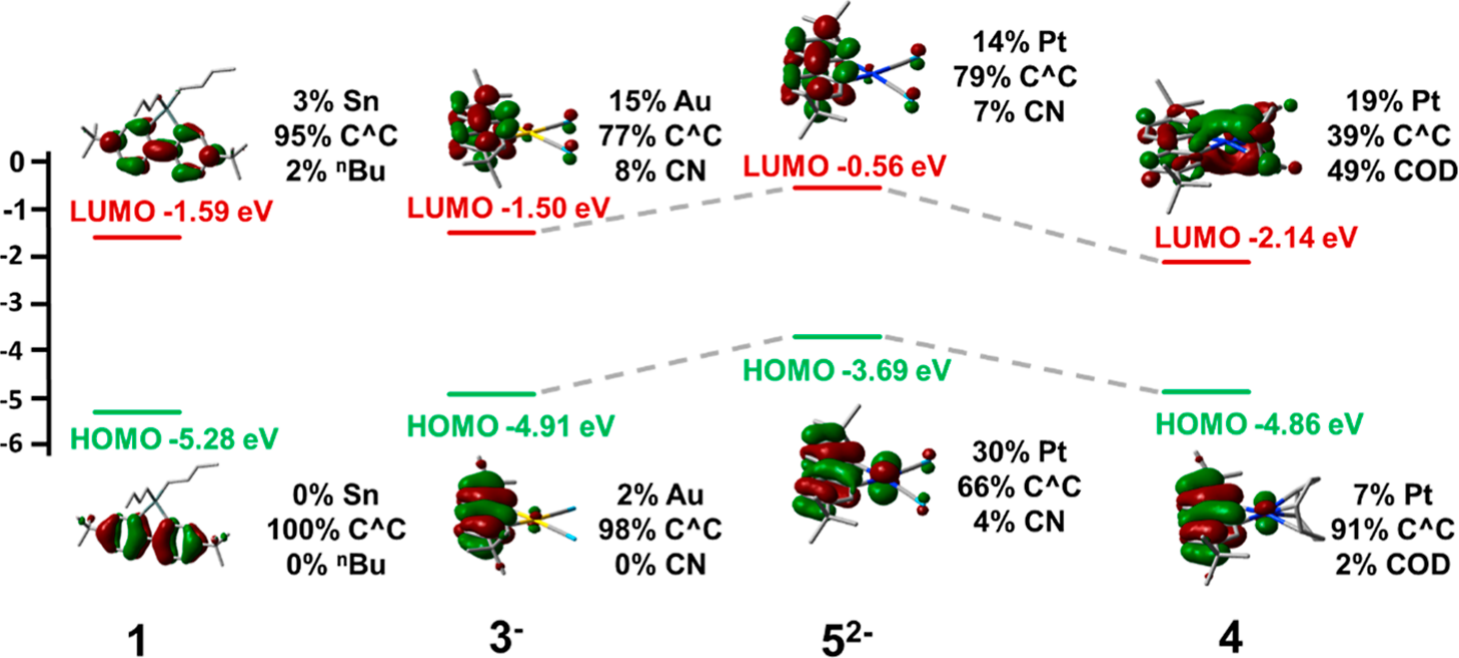
Calculated energies, composition, and images for the frontier orbitals of **1**, **3^‑^**, **5** ^**2–**^, and **4** in CH_2_Cl_2_.

It must be noted already that despite their structural similarities, the electronic structures of this series of complexes show remarkable differences, and these differences are also present in their photoluminescent properties. The introduction of the heavy sixth-period ions, Au­(III) and Pt­(II), with high spin–orbit coupling, enhances intersystem crossing. Thus, the emissions of complexes **2** and **4** are clearly phosphorescent, exhibiting structured bands in the green and yellow/orange regions, respectively (Figure S23). Introducing cyanide ligands increases the emission intensity. As shown in Figures 6 and S24a, complexes **3** and **5** show bright green phosphorescence in the solid state and in a PMMA matrix. The emission bands appear at very similar energies, with the slight red shift for **5** compared to **3**, which is consistent with a higher energy of the SOMO-1 orbital due to the greater participation of the platinum orbitals.

**Figure 6:**
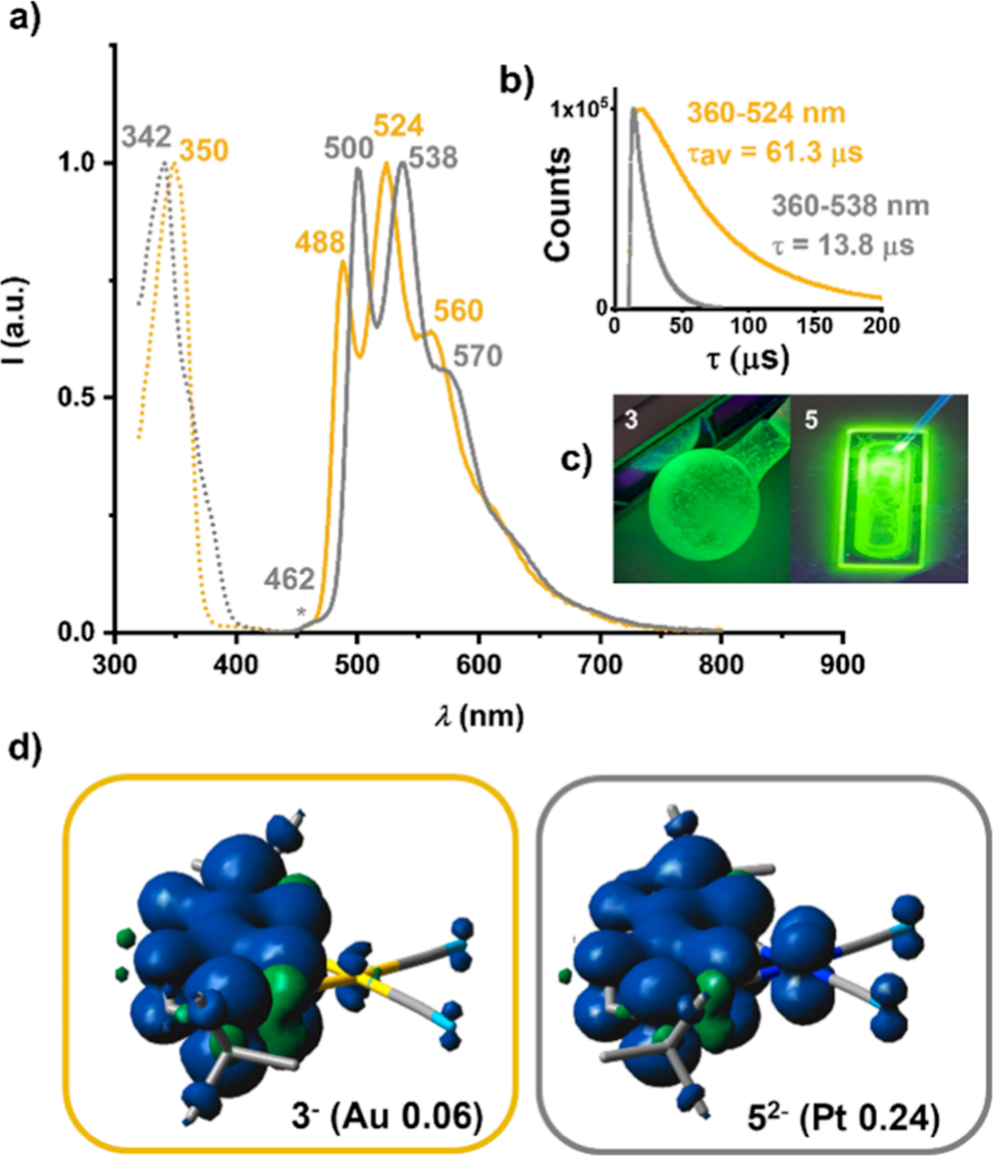
(a) PL spectra (solid lines for emission and dotted lines for excitation) of complex **3** (gold) and **5** (gray) in PMMA (1%). (b) Decay curves of the emission bands for the same films. (c) Pictures of complexes **3** and **5** under UV (365 nm) illumination. (d) Spin density plots for ions **3^‑^** and **5** ^**2–**^.

Despite similarities in the steady-state spectra, time-resolved measurements of the photoluminescence reveal a striking difference. The gold complex **3** exhibits a much longer emission lifetime than the platinum complex **5** in PMMA [51.8 (67%), 80.6 (33%) μs **3** vs. 13.8 μs **5**] (Figure 6) and in the solid state [106.4 μs **3** vs. 11.0 (75%), 3.4 (25%) μs **5**] (Figure S24b). Theoretical calculations provide crucial insight into this distinction, which is a hallmark of the two families. The vibrational structure, Stokes shifts, and prior knowledge suggest ^3^LC­(C^C) transitions. However, while the SOMO and SOMO-1 of the [Au­(C^C)­(CN)_2_]^−^ anion (**3^‑^**, gas phase: SOMO 93% C^C, SOMO-1 98% C^C) are predominantly localized on the C^C fragment, a fact summarized by the spin density plot in Figure 6d, indicating Au­(III) acts as a mere spectator, the frontier orbitals of the [Pt­(C^C)­(CN)_2_]^2–^ dianion (**5** ^**2–**^, gas phase: SOMO 92% C^C, SOMO-1 78% C^C, 19% Pt) show, similarly to the description for the S_0_→S_1_ transition, significant ^3^MLCT character. This is reflected in a 0.24 Pt contribution to the corresponding spin density plot (Figure 6d). Consequently, our calculations not only predict the red-shifted emission for **5** (500 nm) versus **3** (488 nm) but also rationalize the difference in phosphorescence lifetime, unequivocally attributing it to the distinct metal participation in the frontier orbitals.

Another striking feature, visible to the naked eye, is the pronounced sensitivity of the emission to air. Drying the PMMA films under a stream of N_2_ while illuminating with UV light (365 nm) caused a dramatic increase in emission intensity at the point of N_2_ incidence. This effect is even more dramatic for a pure sample of **5** deposited on a slide (see Figure 6c and video in the Supporting Information) and also very noticeable for crystalline **3**, though difficult to capture on camera. Finally, as can be seen in Figure 6a, the emission spectrum of **5** in PMMA includes a minor high-energy feature at 462 nm, which we assign, by extrapolation to the observations in solution (discussed below), to residual ^1^IL­(C^C) fluorescence. These high-energy features are also noticeable in the solid samples, as can be seen in Figure S24.

The potassium salts **3** ^**K**^ and **5** ^**K**^ exhibit emission profiles very similar to, though slightly red-shifted compared to, their NBu_4_^+^ analogues **3** and **5** (Figure S25a,b). As can be seen in Figure S25c,d, this apparent red-shift of the ^3^IL­(C^C) transition originates from the admixing of the structured green band with a lower-energy component (λ_em_ = 564 nm for **3** ^**K**^, λ_ex_ = 420 nm; λ_em_ = 514sh, 576 nm for **5** ^**K**^, λ_ex_ = 452 nm). These lower-energy components likely arise from packing-induced transitions facilitated by the close proximity of anions in the potassium salts, as seen in the crystal structure of **3** ^**K**^ and suggested by the IR data.

For complex **3** in solution, excitation in the lowest energy band produces an intense green photoluminescence in both Me-THF (Figure 7a) and CH_2_Cl_2_ (Figure S26a). The emission spectrum results from the convolution of a high-energy fluorescent component (lifetime ∼ 2.8 ns in CH_2_Cl_2_) and a structured, lower-energy band. Given its similarity to the high-energy band of complex 1, the former is assigned to a ^1^IL­(C^C) transition, and the structured lower energy band is assigned to ^3^IL­(C^C) phosphorescence, a conclusion supported by the excellent agreement between the calculated emission maximum in CH_2_Cl_2_ (482 nm) and the first peak of the recorded band (492 nm).

**Figure 7:**
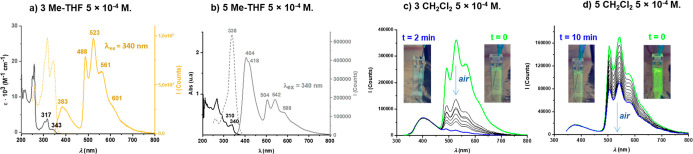
(a) Absorption, excitation, and emission spectra of complex **3** in Me-THF (5 × 10^–4^ M). (b) Absorption, excitation, and emission spectra of complex **5** in Me-THF (5 × 10^–4^ M). (c) Emission spectra of complex **3** in CH_2_Cl_2_ (5 × 10^–4^ M) freshly deoxygenated (green line, t = 0, see picture) and after opening the cuvette to air (gray lines) to finish with the emission spectra after 2 min exposed to air (blue line, t = 2 min, see picture). (d) Emission spectra of complex **5** in CH_2_Cl_2_ (5 × 10^–4^ M) freshly deoxygenated (green line, t = 0, see picture) and after opening the cuvette to air (gray lines) to finish with the emission spectra after 10 min exposed to air (blue line, t = 10 min, see picture).

Like for complex **3**, the emission of **5** in solution reveals a mixture of ^1^IL­(C^C) fluorescence and phosphorescence, but the balance between these states is strongly metal- and solvent-dependent. In CH_2_Cl_2_, the phosphorescence is the main component, with fluorescence appearing only as a shoulder (Figures 7d and S26b). In Me-THF, the fluorescence band is predominant (Figure 7b), allowing us to determine its lifetime (3.7 ns).

The differing susceptibilities to oxygen quenching provide a clear distinction between the singlet and triplet natures of these emissions. Upon exposure to air, the CH_2_Cl_2_ solution of complex **3** exhibits rapid quenching of the phosphorescent component; the fluorescence becomes predominant, and the naked eye emission color shifts from green to cyan (Figure 7c). For complex **5** under the same conditions, this effect is less pronounced, and the phosphorescence remains the dominant emissive pathway even after 10 min of air exposure.

A key motivation for preparing the potassium salts **3** ^**K**^ and **5** ^**K**^ was to translate the photophysical properties of these anionic complexes into aqueous solution. This translation was successful, as shown in Figure S26c,d. Complex **3** ^**K**^ exhibits intense ^3^IL­(C^C) phosphorescence in deoxygenated water, which is dramatically quenched upon air exposure. Consistent with the behavior in polar organic solvents (Me-THF), the emission of **5** ^**K**^ in water is dominated by the fluorescent band at 392 nm, and the quenching of its weak phosphorescent component is far less marked.

A coherent hypothesis explaining all these observations is as follows. For both the Au­(III) and Pt­(II) anions, the high spin–orbit coupling (SOC) of the metal center promotes rapid intersystem crossing (ISC) from S_1_ to T_1_. However, the nature of the T_1_ state dictates the subsequent photophysical behavior. For gold, the T_1_ state possesses pure ^3^IL­(ĈC) character. For platinum, the T_1_ state has mixed ^3^IL/^3^MLCT character. Consequently, the quenching by oxygen is far more dramatic for the gold complex, whose long-lived, pure ^3^IL­(C^C) triplet state is highly susceptible to energy transfer to ^3^O_2_, compared to the charge-transfer, shorted-lived phosphorescent platinum complex. This hypothesis is further supported by the direct determination of the singlet oxygen generation by monitoring the intensity of the emission of ^1^O_2_ at 1270 nm (see Section S5 in the Supporting Information). Complex **3** in oxygenated CH_3_CN (5 × 10^–5^ M) shows a value of 95% vs phenalenone (λ_ex_ 348 nm), while complex **5** (5 × 10^–5^ M) in oxygenated CH_3_CN shows a value of 36% at 334 nm and 45% at 342 nm.

All these features, the different natures of the triplet states, and, as a consequence, the different efficiencies in ^1^O_2_ generation are critical for understanding the photocatalytic activity of these complexes.

### Electrochemistry

Cyclic voltammetry studies were carried out in CH_2_Cl_2_ (5 × 10^–4^ M) at room temperature for complexes **1**, **3**, and **5**. Details of the experiments carried out can be found in the Supporting Information (Section S6 and Table S35). In all cases, the electrochemical window of the solvent allows only seeing the oxidation processes. As can be seen in Figure 8 and section S6, tin complex **1** shows an irreversible oxidation with E_pa_ at +1.42 V that is attributed to be a ligand-centered redox process and gives a value of −5.775 eV for the E_HOMO_, slightly higher than the prediction by DFT calculations. For the gold monoanionic complex **3**, the presence of a quasi-reversible oxidation at +1.28 V, gives a value of −5.648 eV for the E_HOMO_, again, higher than the calculated value. The cathodic shift with respect to the neutral complex **1** and with respect with other neutral biphenyl Au­(III) complexes^37^ is consistent with the anionic nature of complex **3**. Given the nature of the HOMO predicted by DFT calculations, this oxidation is centered in the C^C ligand. In sharp contrast, the platinum complex **5** shows three irreversible oxidation waves with E_pa_ at +0.52, 0.96, and 1.27 V. The first oxidation matches very well with the calculated energy of the HOMO (−4.872 eV vs −4.86 eV) that, as was discussed before, is an orbital with a contribution of 66% (C^C) and 30% (Pt), so the first oxidation could be attributed to metal-perturbed C^C centers. The second and third one-electron oxidations are tentatively associated with Pt­(II)→Pt­(III) and Pt­(III)→Pt­(IV) processes, which is coherent with the nature of HOMO–1 (56% Pt) and HOMO–2 (94% Pt) and with previous studies.^21^

**Figure 8:**
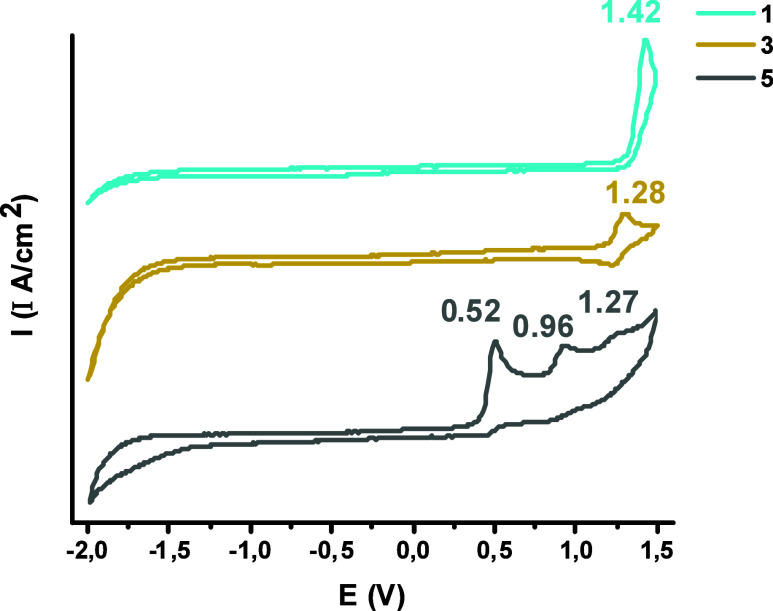
Cyclic voltammetry of **1**, **3**, and **5** in CH_2_Cl_2_ (5 × 10^–4^ M).

### Photocatalytic Studies

Phosphorescent cyclometalated transition metal complexes are widely employed as sensitizers in energy transfer and photoredox catalysis, a field historically dominated by Ru­(II), Ir­(III), and Pt­(II) complexes.^38–41^ In contrast, Au­(III) phosphorescent complexes have been significantly less explored in this context. This is intriguing, given that Au­(III) complexes often feature long-lived ^3^IL excited states (with lifetimes up to hundreds of microseconds) and benefit from the large spin–orbit coupling constant of gold (around 5100 cm^–1^), which promotes highly efficient intersystem crossing (ISC). Consequently, the emergence of promising Au­(III) cyclometalated photocatalysts is not surprising.^42,43^

The photo-oxidation of sulfides to sulfoxides using molecular oxygen is a reaction of significant interest due to its relevance in synthesizing biologically active compounds and has been broadly studied, which makes it ideal as a model. This transformation typically proceeds via the generation of reactive oxygen species (ROS), such as singlet oxygen (^1^O_2_) or superoxide (O_2_^•–^), through energy or electron transfer from an excited photocatalyst to dissolved oxygen. As previously discussed, all complexes in this study are sensitive to oxygen, but the long-lived ^3^IL­(ĈC) phosphorescent state of the Au­(III) series transfers energy to ^3^O_2_ with more efficacy than the mixed short-lived ^3^IL/^3^MLCT Pt­(II) state. The investigation of the different activity of **3** (Table 2, entry 2) and **5** (Table 2, entry 1) in the photooxidation of *p*-bromothioanisole (Scheme 2) in CD_3_OD reflects this feature, with the gold complex **3** showing much higher catalytic activity compared with **5** (see section S5 for the detailed methodology of all the photocatalytic studies).

**Table 2:** Homogeneous UV-Light Oxidative Reactions with Different Conditions

Entry	Cat. (%)	Solvent	λ = 350 nm	Atm	*t*	% Conv
1	**5** (5)	CD_3_OD	+	O_2_	3 h	8
2	**3** (5)	CD_3_OD	+	O_2_	3 h	82
3	**3** ^**K**^ (1)	CD_3_OD/D_2_O (1:1)	+	O_2_	6 h	95
4	**3** ^**K**^ (5)	CD_3_OD/D_2_O (1:1)	+	O_2_	2.5 h	97
5	**3** ^**K**^ (0)	CD_3_OD/D_2_O (1:1)	+	O_2_	2.5 h	0
6	**3** ^**K**^ (1)	CD_3_OD/D_2_O (1:1)	-	O_2_	6 h	0
7	**3** ^**K**^ (5)	CD_3_OD/D_2_O (1:1)	+	N_2_	2.5 h	5
8^a^	**3** ^**K**^ (5)	CD_3_OD/D_2_O (1:1)	+	O_2_	2.5 h	4
9^b^	**3** ^**K**^ (5)	CD_3_OD/D_2_O (1:1)	+	O_2_	2.5 h	∼1

a DABCO, 3 equiv.

b BQ, 3 equiv.

**Scheme 2:**
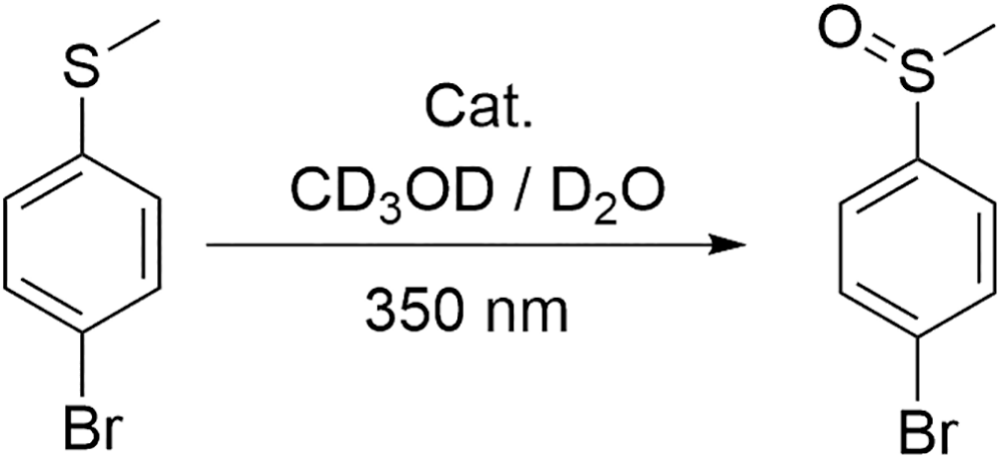
Photooxidation of *p*-Bromothioanisole to the Corresponding Sulfoxide

Developing catalysts that operate in environmentally benign solvents like water is a key objective. After confirming the superior activity of **3** vs **5** but leveraging the water solubility of **3** ^**K**^, along with its thermal and photochemical robustness, we decided to investigate in detail its performance in the photooxidation of *p*-bromothioanisole under different conditions.

Complex **3** ^**K**^ effectively catalyzes the selective oxidation of *p*-bromothioanisole to the sulfoxide with a catalyst loading of just 1%, achieving high conversion in 6 h. With a 5% loading, the reaction is complete in 2.5 h. This performance is comparable to recent reports on water-soluble Pt­(II) porphyrin-based photosensitizers.^44^

While Messerle et al. have proposed mechanisms involving metal-O_2_ or metal–sulfur interactions for the catalytic transformation of thioanisole,^45^ and the latter has been invoked to explain the activity of certain Au­(I) complexes,^46^ but such affinity for sulfur is not characteristic of Au­(III). Therefore, the high photocatalytic activity of **3** ^**K**^ must be attributed primarily to the efficient energy transfer from its long-lived triplet excited state to molecular oxygen, facilitated by the strong spin–orbit coupling of gold.

To elucidate the mechanism, we sought to identify the reactive oxygen species involved. The photocatalytic reaction was evaluated in the presence of specific quenchers: the singlet oxygen quencher 1,4-diazabicyclo[2.2.2]­octane (DABCO, 3 equiv.; Table 2, Entry 8) and the superoxide radical quencher 1,4-benzoquinone (BQ, 3 equiv.; Table 2, Entry 9). In both cases, a significant decrease in reaction yield was observed. This inhibition by both quenchers suggests that both ^1^O_2_ and O_2_^•‑^ are generated by **3** ^**K**^ and contribute to the oxidation process.

## Conclusions

In summary, we have synthesized and characterized a series of anionic d^8^ complexes, [Au­(C^C)­(CN)_2_]^−^ 3^‑^ and [Pt­(C^C)­(CN)_2_]^2–^ **5** ^**2–**^ (C^C = 4,4′-ditert-butylbiphenyl-2,2′-diyl), along with their NBu_4_^+^ and K^+^ salts. These complexes were prepared from the chloride-bridged dimer [Au­(C^C)­Cl]_2_ and the novel complex [Pt­(C^C)­(COD)] (**4**), both accessible via transmetalation from Sn­(C^C)^n^Bu_2_ **1**.

A comparative photophysical study revealed that while the emissions are predominantly localized on the C^C chromophore, the nature of the metal center dictates key excited-state properties. The tin precursor **1** exhibits deep-blue ^1^IL­(C^C) fluorescence with additional low-energy features tentatively assigned to excimer emission. In contrast, the gold and platinum complexes display green phosphorescence. A critical distinction lies in the character of their triplet states: the platinum complexes exhibit mixed ^3^IL/^3^MLCT emission with notably shorter lifetimes, whereas the gold complexes feature pure ^3^IL­(C^C) phosphorescence with substantially longer lifetimes. Besides, the electrochemistry of both families is also different, with gold showing one quasi-reversible oxidation wave and platinum showing up to three irreversible oxidations.

These fundamental differences manifest in a higher sensitivity of the gold complexes to oxygen quenching via energy transfer and ^1^O_2_ generation. Therefore, the water-soluble complex K­[Au­(C^C)­(CN)_2_] **3** ^**K**^ emerges as an efficient photosensitizer for the photo-oxidation of *p*-bromothioanisole in a methanol/water mixture. This work underscores the distinct photophysical signatures of isoelectronic Au­(III) and Pt­(II) centers within the same ligand framework and highlights the potential of anionic Au­(III) complexes as effective photocatalysts in green solvents.

## Experimental Section

### General Considerations

When required, manipulations were performed by using standard Schlenk techniques under dry N_2_. All solvents were dried by means of the appropriate method. Chloroform-d was dried with activated 4 Å molecular sieves before using. 2,2′-Dibromo-4,4′-ditert-butylbiphenyl, (C^C)­Sn^n^Bu_2_ **1**, [Au­(C^C) Cl]_2_ **2** ^17^ and PtCl_2_COD^47^ were synthesized according to literature procedures. Single crystals of (C^C)­Sn^n^Bu_2_ **1** colorless blocks were grown by slow diffusion of hexane in a solution of the crude material in CH_2_Cl_2_ at room temperature. A block with dimensions 0.35 × 0.24 × 0.13 mm was selected for X-ray diffraction analysis. Details of the results are included in Table S1 in the Supporting Information. The microanalyses were carried out with a CA FLASH 2000 (Thermo Fisher Scientific) microanalyzer. Infrared spectra were recorded using a PerkinElmer Spectrum 65 FT-IR spectrometer with a diamond ATR attachment. MALDI-TOF spectra were collected in a Microflex MALDI-TOF Bruker spectrometer in the negative ion mode. ^1^H, ^13^C­{^1^H}, ^195^Pt NMR experiments were recorded using a Bruker DPX–400 spectrometer equipped with a ^1^H,BB smartprobe and Z-gradients. NMR spectra are referenced to the residual protons of the deuterated solvent. The numbering scheme used for the NMR is shown below.

Cyanide salts are acutely toxic. All reactions must be conducted in a certified fume hood following safety protocols, including the use of gloves and dedicated waste streams. Exposure to acidic conditions must be strictly avoided to prevent the formation of the hazardous hydrogen cyanide gas.
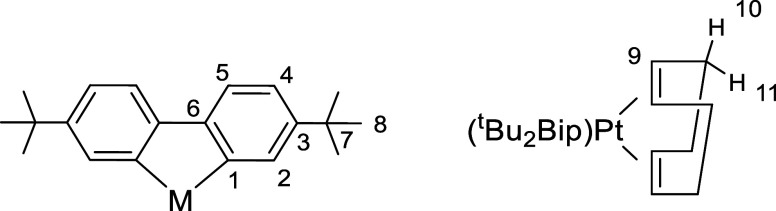


#### Synthesis of NBu_4_[Au­(C^C)­(C≡N)_2_] (3)

[Au­(C^C)­Cl]_2_ **2** (0.321 g, 0.323 mmol) and NBu_4_CN (0.347 g, 1.292 mmol) were mixed in 20 mL methanol, and the reaction mixture was stirred at 65̊C for 30 min. The resulting solution was filtered through Celite, and the filtrate was evaporated to dryness. The residue obtained was crushed and washed with ethanol (ca. 3 × 5 mL) and diethyl ether (ca. 3 × 5 mL) to give **3** as a white solid that was filtered and air-dried (0.342 g, 70%). Anal. Calcd for C_38_H_60_N_3_Au (755.87): C, 60.38; H, 8.00; N, 5.56. Found: C, 59.96; H, 8.17; N, 5.37. IR (cm^–1^): ν­(C≡N) 2147 (br, w). MALDI-TOF (−): *m*/*z* (%) 514.4 [M-NBu_4_+H] (100%), 250.7 [HAu­(CN)_2_] (76%). Exact Mass (−): *m*/*z* 513.1613. ^1^H NMR ((CD_3_)_2_CO), 400 MHz, 298 K): δ 8.27 (d, ^4^J_H–H_ = 2.1 Hz, 2H, H^2^), 7.25 (d, ^3^J_H–H_ = 8.0 Hz, 2H, H^5^), 7.12 (dd, ^3^J_H–H_ = 8.0, ^4^J_H–H_ = 2.1 Hz, 2H, H^4^), 3.48 (m, 8H, N–CH_2_–(CH_2_)_2_CH_3_, NBu_4_^+^), 1.82 (m, 8H, NCH_2_–(CH_2_)–CH_2_CH_3_, NBu_4_^+^), 1.43 (m, 8H, N­(CH_2_)_2_–(CH_2_)–CH_3_, NBu_4_^+^), 1.31 (s, 18H, H^8^), 0.98 (t, 12H, N­(CH_2_)_3_-CH_3_, NBu_4_^+^). ^13^C­{^1^H} NMR ((CD_3_)_2_CO), 100 MHz, 298 K) δ 155.1 (C^1^), 153.6 (C^3 or 6^), 149.9 (C^6 or 3^), 141.0 (CN), 136.2 (C^2^), 124.1 (C^4^), 120.6 (C^5^), 59.5 (N–CH_2_–(CH_2_)_2_CH_3_, NBu_4_^+^), 35.3 (C^7^), 31.8 (C^8^), 24.6 (NCH_2_–(CH_2_)–CH_2_CH_3_, NBu_4_^+^), 20.4 (N­(CH_2_)_2_–(CH_2_)–CH_3_, NBu_4_^+^), 13.9 (N­(CH_2_)_3_-CH_3_, NBu_4_^+^).

Single crystals of NBu_4_[Au­(C^C)­(C≡N)_2_] (**3**): Colorless needles were grown by slow diffusion of MeOH in a saturated solution of the crude material dissolved in CH_2_Cl_2_ at room temperature. A needle with dimensions 0.33 × 0.19 × 0.01 mm was selected for X-ray diffraction analysis. Details of the results are included in Table S1 in the Supporting Information.

**Synthesis of K­[Au­(C^C)­(C≡N)_2_] (3^K^)**

Following the same procedure described for **3** but using [Au­(C^C)­Cl]_2_ **2** (0.3145 g, 0.316 mmol) and KCN (0.082 g, 1.264 mmol). Complex **3** ^**K**^ was isolated as a white solid (0.231 g, 66%). Anal. Calcd for C_22_H_24_N_2_AuK (552.50): C, 47.83; H, 4.38; N, 5.07. Found: C, 47.40; H, 4.32; N, 5.00. IR (cm^–1^): ν­(C≡N) 2162 (w), 2144 (m). MALDI-TOF (−): *m*/*z* (%) 1053.7 [2M-2K + CN + H] (56%), 517.0 [M-K+3H] (100%). Exact Mass (−): *m*/*z* 513.1607 ^1^H NMR ((CD_3_)_2_CO), 400 MHz, 298 K): δ 8.25 (d, ^4^J_H–H_ = 2.0 Hz, 2H, H^2^), 7.24 (d, ^3^J_H–H_ = 8.0 Hz, 2H, H^5^), 7.12 (dd, ^3^J_H–H_ = 8.0, ^4^J_H–H_ = 2.0 Hz, 2H, H^4^), 1.30 (s, 18H, H^8^). ^13^C­{^1^H} NMR ((CD_3_)_2_CO), 100 MHz, 298 K): δ 155.0 (C^1^), 153.5 (C^3 or 6^), 149.9 (C^6 or 3^), 141.1 (CN), 136.1 (C^2^), 124.1 (C^4^), 120.6 (C^5^), 35.2 (C^7^), 31.8 (C^8^).

Crystals of K­[Au­(C^C)­(C≡N)_2_] (**3** ^**K**^): Colorless needles were growth by slow diffusion of diisopropyl ether in a saturated solution of the crude material dissolved in acetone. A needle with dimensions of 0.5 × 0.27 × 0.05 mm was selected for X-ray diffraction analysis. Unfortunately, the data are not good enough for publishing and are not included in the Supporting Information or deposited in the CCDC. The data were used for connectivity only.

^13^C­{^1^H} NMR ((CD_3_)_2_CO), 75.5 MHz, 298 K) of K­[Au­(C^C)­(^13^C≡N)_2_] (3^K^*)

In an NMR tube, 0.6 mL of (CD_3_)_2_CO, [Au­(C^C)­Cl]_2_ **2** (10 mg, 0.010 mmol), and K^13^CN (0.020 mmol) were mixed and sonicated for 5 min until the complete solution of the Au precursor is observed. The ^13^C­{^1^H} NMR spectrum is recorded with 32 scans to unequivocally assign the ^13^CN signal at δ 141.1 ppm.

#### Synthesis of Pt­(C^C)­(COD) (4)

Method A: To 2,2′-Dibromo-4,4′-ditert-butylbiphenyl (0.561 g, 1.33 mmol) dissolved in 60 mL of diethyl ether, 1.33 mL of ^n^BuLi (2.5 M) was added dropwise at 0 °C and stirred at room temperature for 1 h. After this time, the reaction mixture was cooled down to −90 °C, PtCl_2_(COD) (0.642 g, 1.33 mmol) was added, and then the reaction mixture was gently warmed up and stirred for 12 h at room temperature in a strict N_2_ atmosphere. After this time, the volatiles are evaporated, and the brownish residue was dissolved in CH_2_Cl_2_ and filtrate through Celite. The CH_2_Cl_2_ was evaporated, and the residue was crushed with Et_2_O/Hexane (3:7) to give **4** as an orange solid (0.303 g, 40%).

Method B: [PtCl_2_COD] (0.771 g, 1.60 mmol) and (C^C)­Sn^n^Bu_2_ 1 (0.800 g, 1.60 mmol) were mixed in 30 mL of CH_2_Cl_2_, and the reaction mixture was stirred at 62 °C for 24 h. The resulting solution was treated with a chromatographic column (R_f_ = 0.6) using a mixture of ethyl acetate/hexane (1/9) as eluent. The tubes with the product were evaporated to dryness to give **4** as an orange solid (0.3628 g, 40%).

Anal. Calcd for C_28_H_39_Pt (567.67): C, 60.66; H, 7.55. Found: C, 61.01; H, 7.26. MALDI-TOF (+): *m*/*z* (%) 568.6 [M + H] (100%). ^1^H NMR ((CD_3_)_2_CO), 400 MHz, 298 K): δ 7.17 (d, ^4^J_H–H_ = 2.4 Hz, ^3^J_H–Pt_ = 56 Hz, 2H, H^2^) overlapped with 7.17 (d, ^3^J_H–H_ = 7.5 Hz, ^4^J_H–Pt_ = 20 Hz, 2H, H^5^), 6.99 (dd, *J* = 2.4 Hz, 7.5 Hz, 2H, H^4^), 5.57 (m, ^3^J_H–Pt_ = 40 Hz, 4H, H^9^), 2.7–2.5 (m, 8H, 4H^10^, 4H^11^), 1.27 (s, 18H, H^8^). ^13^C NMR ((CD_3_)_2_CO), 101 MHz, 298 K): δ 157.9 (^1^J_C–Pt_ = 1112 Hz, C^1^), 154.3 (^2^J_C–Pt_ = 122 Hz, C^3 or 6^), 148.5 (^3^J_C–Pt_ = 53 Hz, C^6 or 3^), 130.5 (^3^J_C–Pt_ = 44 Hz, C^4^), 123.9 (^4^J_C–Pt_ = 6 Hz, C^5^), 119.8 (^2^J_C–Pt_ = 64 Hz, C^2^), 104.1 (^2^J_C–Pt_ = 58 Hz, C^9/10^), 35.1 (C^7^), 31.8 (C^8^).

Single crystals of Pt­(C^C)­(COD) (**4**): Orange blocks were grown by slow evaporation of a solution of the crude material in a mixture of Et_2_O/Hexane (3:7) at room temperature. A block with dimensions 0.38 × 0.24 × 0.15 mm was selected for X-ray diffraction analysis. Details of the results are included in Table S1 in the SI.

**Synthesis of (NBu_4_)_2_[Pt­(C^C)­(C≡N)_2_] (5)**

Pt­(C^C)­(COD) **4** (0.047 g, 0.083 mmol) and NBu_4_CN (0.044 g, 0.165 mmol) were mixed in MeOH, and the reaction mixture was stirred at room temperature for 30 min. The volatiles are vacuum evaporated, and the residue was washed with Et_2_O (ca. 3 × 5 mL) to give **5** as a yellow oil (0.0625 g, 76%). Anal. Calcd for C_54_H_96_N_4_Pt (996.45): C, 65.09; H, 9.71; N, 5.62. Best analysis found: C, 67.13; H, 10.04; N, 5.38. IR (cm^–1^): ν­(C≡N) 2124, 2095. MALDI-TOF (−): *m*/*z* (%) 969.1 [Pt­(C^C)­(C≡N)]_2_ (86%), 514.4 [M-NBu_4_+3H] (100%), 250.3 [Pt­(CN)_2_+2H] (100%). Exact Mass (−): *m*/*z* 512.1677 and Exact Mass (+): *m*/*z* 996.7557. ^1^H NMR ((CD_3_)_2_CO), 400 MHz, 298 K): δ 8.36 (d, ^4^J_H–H_ = 2.1, ^3^J_H–Pt_ = 58 Hz, 2H, H^2^), 7.00 (m, 2H, H^5^), 6.75 (dd, ^3^J_H–H_ = 7.8, ^4^J_H–H_ = 2.1 Hz, 2H, H^4^), 3.37 (m, 8H, N–CH_2_–(CH_2_)_2_CH_3_, NBu_4_^+^), 1.61 (m, 8H, NCH_2_–(CH_2_)–CH_2_CH_3_, NBu_4_^+^), 1.34 (m, 8H, N­(CH_2_)_2_–(CH_2_)–CH_3_, NBu_4_^+^), 1.29 (s, 18H, H^8^), 0.87 (t, 12H, N­(CH_2_)_3_-CH_3_, NBu_4_^+^). ^13^C­{^1^H} NMR ((CD_3_)_2_CO), 100 MHz, 298 K): δ 163.6 (C^1^, ^1^J_Pt–C_ = 867 Hz), 156.7 (C^6^, ^2^J_Pt–C_ = 92 Hz), 146.4 (C^3^, ^3^J_Pt–C_ = 51 Hz), 146.2 (CN, ^1^J_Pt–C_ = 817 Hz), 137.7 (C^2^, ^2^J_Pt–C_ = 61 Hz), 118.54 (C^4^), 117.9 (C^5^, ^3^J_Pt–C_ = 53 Hz), 59.5 (N–CH_2_–(CH_2_)_2_CH_3_, NBu_4_). 34.9 (C^7^), 32.4 (C^8^), 24.9 (NCH_2_–CH_2_–(CH_2_CH_3_), NBu_4_), 20.4 (N-(CH_2_CH_2_)–CH_2_–CH_3_, NBu_4_), 14.1 (N-(CH_2_)_3_-CH_3_, NBu_4_). ^195^Pt NMR ((CD_3_)_2_CO), 87.30 MHz, 298 K): δ −4667.

**Synthesis of K_2_[Pt­(C^C)­(C≡N)_2_] (5^K^)**

Following the same procedure described for **5** but starting from Pt­(C^C)­(COD) **4** (0.050 g, 0.088 mmol) and KCN (0.011 g, 0.171 mmol) **5** ^**K**^ was isolated as a brown foam (0.036 g, 68%). Anal. Calcd for C_22_H_24_N_2_PtK_2_ (589.72): C, 44.81; H, 4.10; N, 4.75. Found: C, 44.52; H, 4.29; N, 4.63. IR (cm^–1^): ν­(C≡N) 2102, 2089. MALDI-TOF (−): *m*/*z* (%) 251.4 [Pt­(CN)_2_+3H] (100%). ^1^H NMR ((CD_3_)_2_CO), 400 MHz, 298 K): δ 8.12 (d, ^4^J_H–H_ = 2.2, ^3^J_H–Pt_ = 54 Hz, 2H, H^2^), 7.00 (d, ^3^J_H–H_ = 7.9 Hz, 2H, H^5^), 6.75 (dd, ^3^J_H–H_ = 7.9, ^4^J_H–H_ = 2.2 Hz, 2H, H^4^), 1.26 (s, 18H, H^8^).

^13^C­{^1^H} NMR ((CD_3_)_2_CO), 75.5 MHz, 298 K) of K_2_[Pt­(C^C)­(^13^C≡N)_2_] (5^K^*)

In an NMR tube, 0.6 mL of (CD_3_)_2_CO, Pt­(C^C)­(COD) **4** (10 mg, 0.017 mmol), and K^13^CN (0.034 mmol) are mixed and sonicated for 5 min until the complete solution of the precursor is observed. The ^13^C­{^1^H} NMR spectrum is recorded with 32 scans to unequivocally assign the ^13^CN signal at δ 151.1 ppm with a ^1^J­(C–^195^Pt) of 802 Hz.

## Supplementary Material





## Notes

Effect of N2 flow on the photoluminescence (λ_ex_ 365 nm) of complex **5** (MP4)
NMR, details about X-ray crystallography, photophysical properties, theoretical calculations, singlet oxygen measurements, and photocatalysis (PDF)

These data can be obtained free of charge from The Cambridge Crystallographic Data Centre via www.ccdc.cam.ac.uk/data_request/cif
