# Supplementary material for: Synthesis and Comparison of the Photophysical Properties of Anionic Diaryl [M(C^C)(CN)2]x– (M= AuIII, PtII) Complexes
Source: Inorg Chem. 2026 May 11;65(20):11024–35. doi: 10.1021/acs.inorgchem.6c00495 (PMC13213907; doi:10.1021/acs.inorgchem.6c00495)
Supplement: Supplementary file 2 [file ic6c00495_si_002.pdf]

SUPPORTING INFORMATION

Synthesis and Comparison of Photophysical Properties of Anionic Diaryl $[M(C^\wedge C)(CN)_2]^{x-}$ ($M = Au^{III}$, Pt^{II}) Complexes

Iker Gil Gomez de Segura[a], Antonio Martín[b], Manfred Bochmann[c], Elena Lalinde[a] and Julio Fernandez-Cestau[a]*

[a] I. Gil Gómez de Segura https://orcid.org/0009-0008-0988-0725, Prof. Dr. E. Lalinde https://orcid.org/0000-0001-7402-1742, Dr. J. Fernandez-Cestau https://orcid.org/0000-0001-7663-6222, Departamento de Química – Instituto de Investigación en Síntesis Química (IQUR), Universidad de La Rioja, E-26006 Logroño, Spain.

b Prof. Dr. Antonio Martín https://orcid.org/0000-0002-4808-574X Instituto de Síntesis Química y Catálisis Homogénea (ISQCH) CSIC, Universidad de Zaragoza, C/ Pedro Cerbuna 12, Zaragoza 50009, Spain.

c Prof. Dr. M. Bochmann https://orcid.org/0000-0001-7736-5428, School of Chemistry, University of East Anglia, Norwich Research Park, Norwich NR4 7TJ, UK.

E-mail: juliofernandez50@gmail.com

**NMR and Mass Spectras**	**S1**
**X-ray crystallography**	**S2**
**Photophysical properties**	**S3**
**Theoretical calculations**	**S4**
**Singlet Oxygen Measurements**	**S5**
**Electrochemical Properties**	**S6**
**Photocatalysis**	**S7**
**References**	

S1. NMR and Mass Spectras

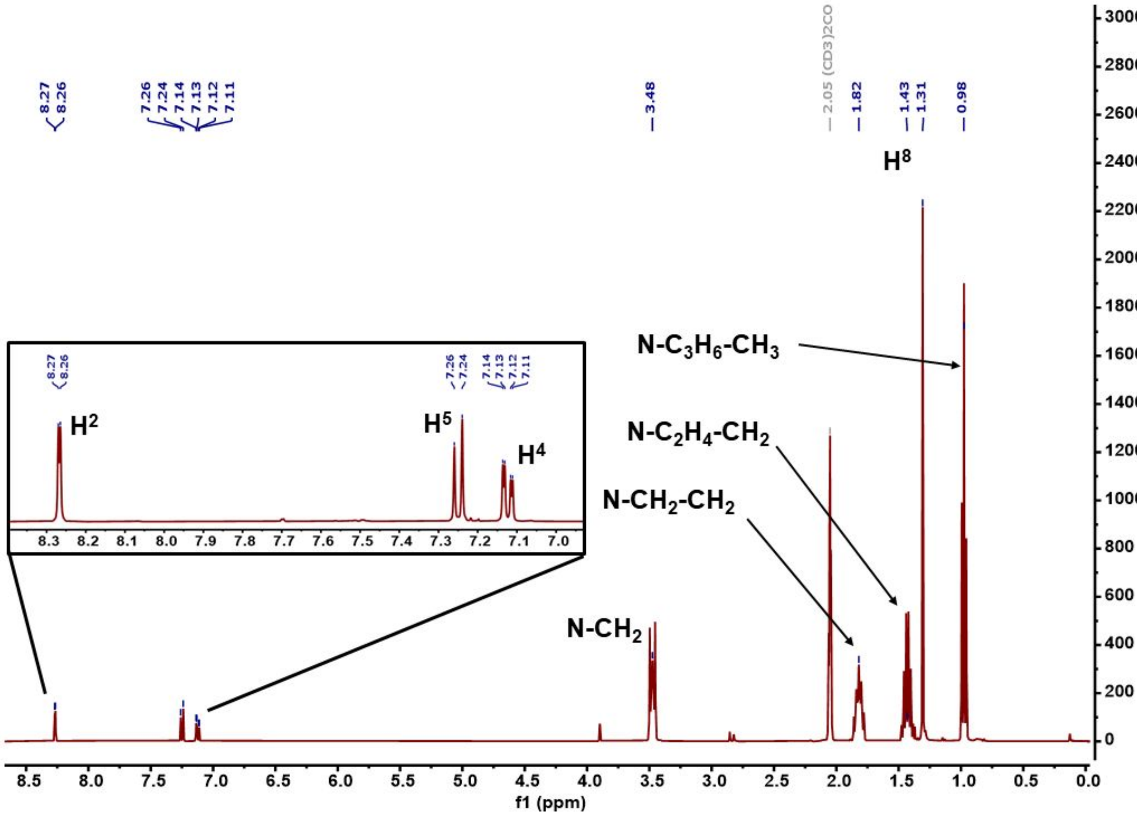

Figure S1. ^{1}H NMR spectrum of **NBu$_4$[Au(C^C)(C≡N)$_2$] 3**, $(CD_3)_2CO$.

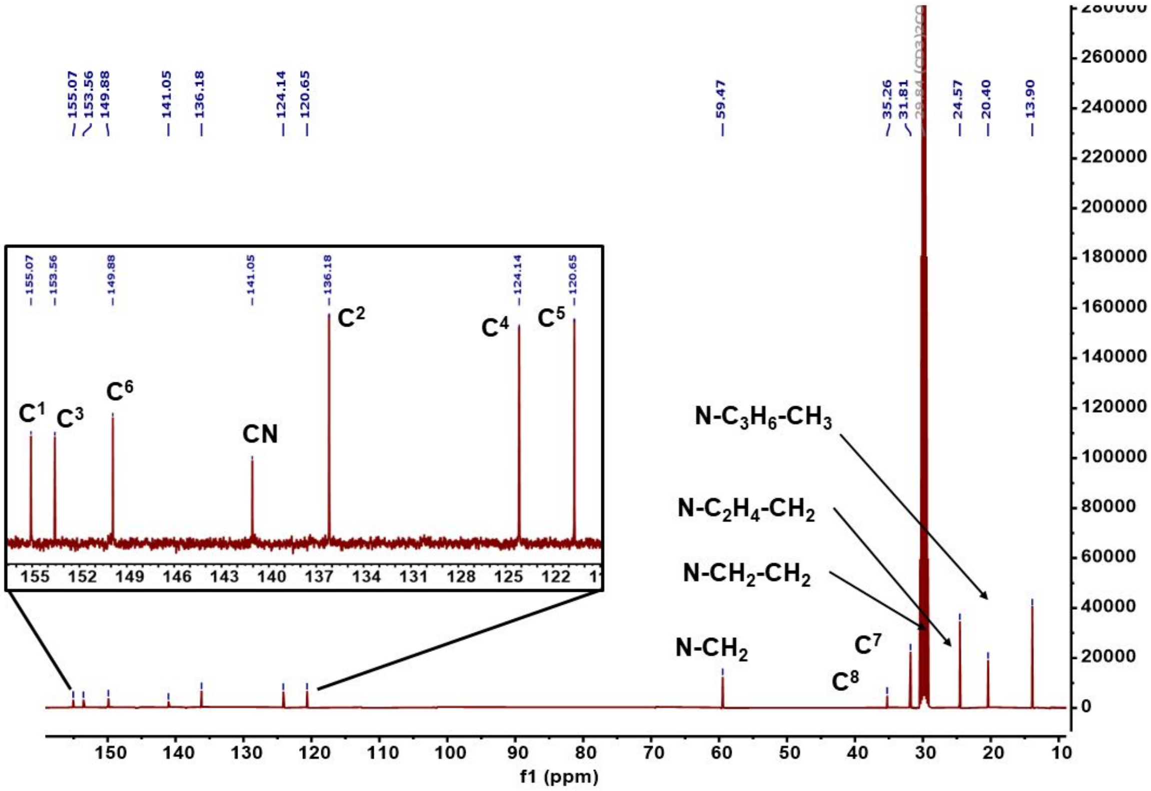

Figure S2. ^{13}C NMR spectrum of **NBu$_4$[Au(C^C)(C≡N)$_2$] 3**, $(CD_3)_2CO$.

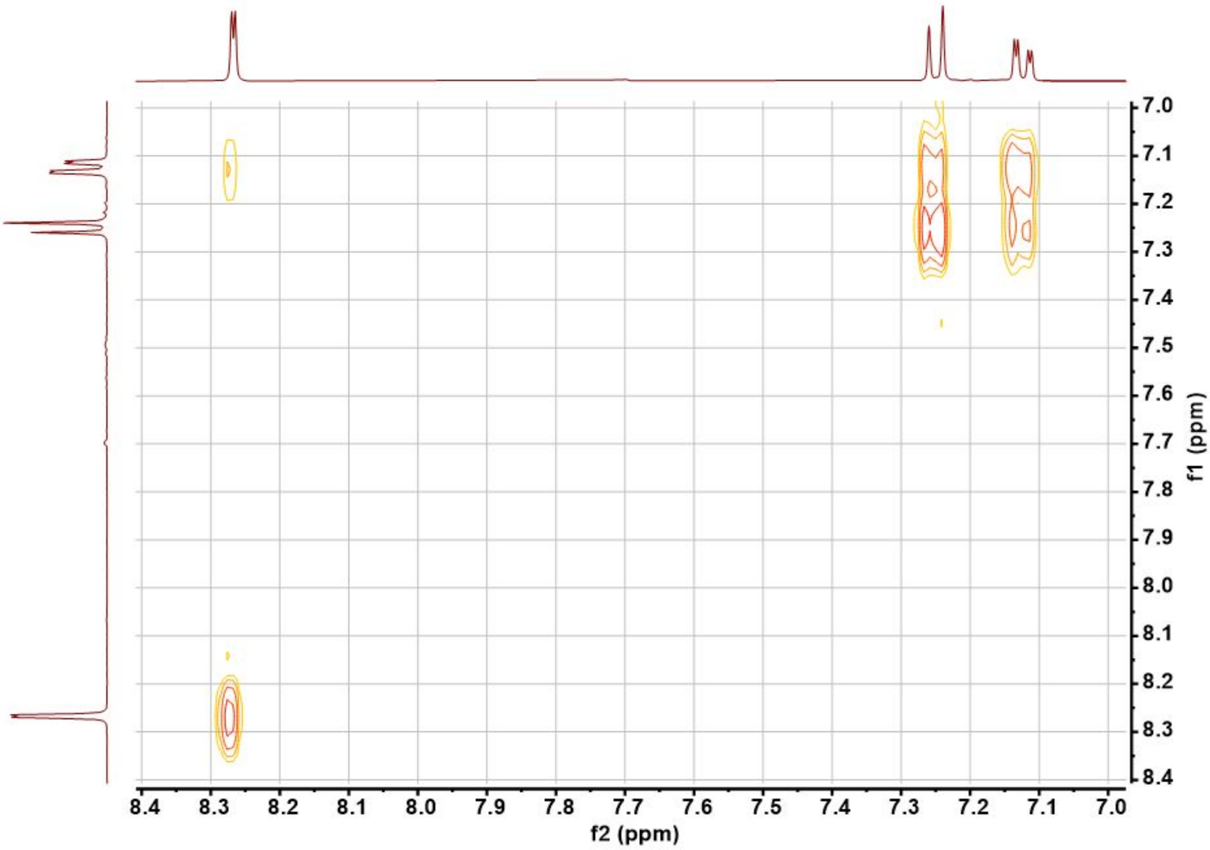

Figure S3. COSY NMR spectrum of **$NBu_4[Au(C^\wedge C)(C{\equiv}N)_2]$ 3**, $(CD_3)_2CO$.

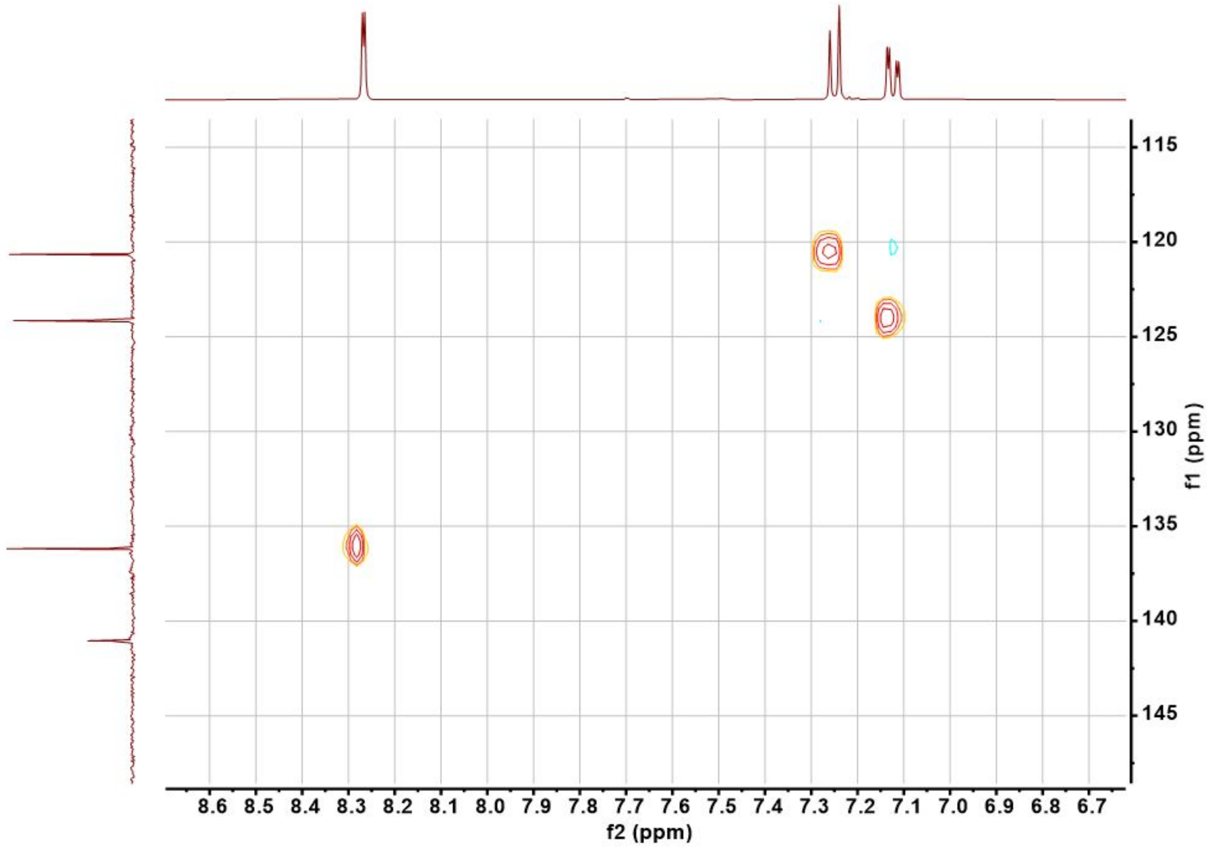

Figure S4. HSQC NMR spectrum of **$NBu_4[Au(C^\wedge C)(C{\equiv}N)_2]$ 3**, $(CD_3)_2CO$.

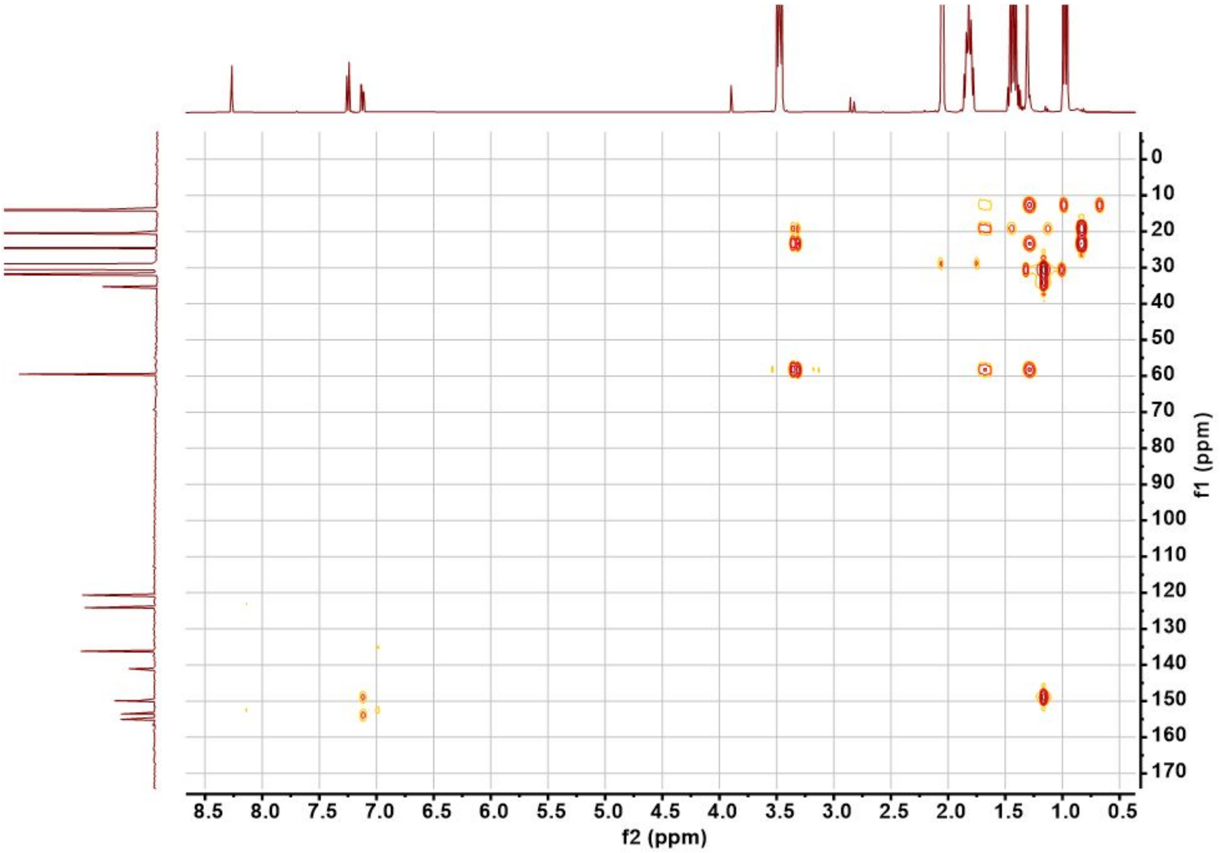

Figure S5. HMBC NMR spectrum of **NBu$_4$[Au(C^C)(C≡N)$_2$] 3**, $(CD_3)_2CO$.

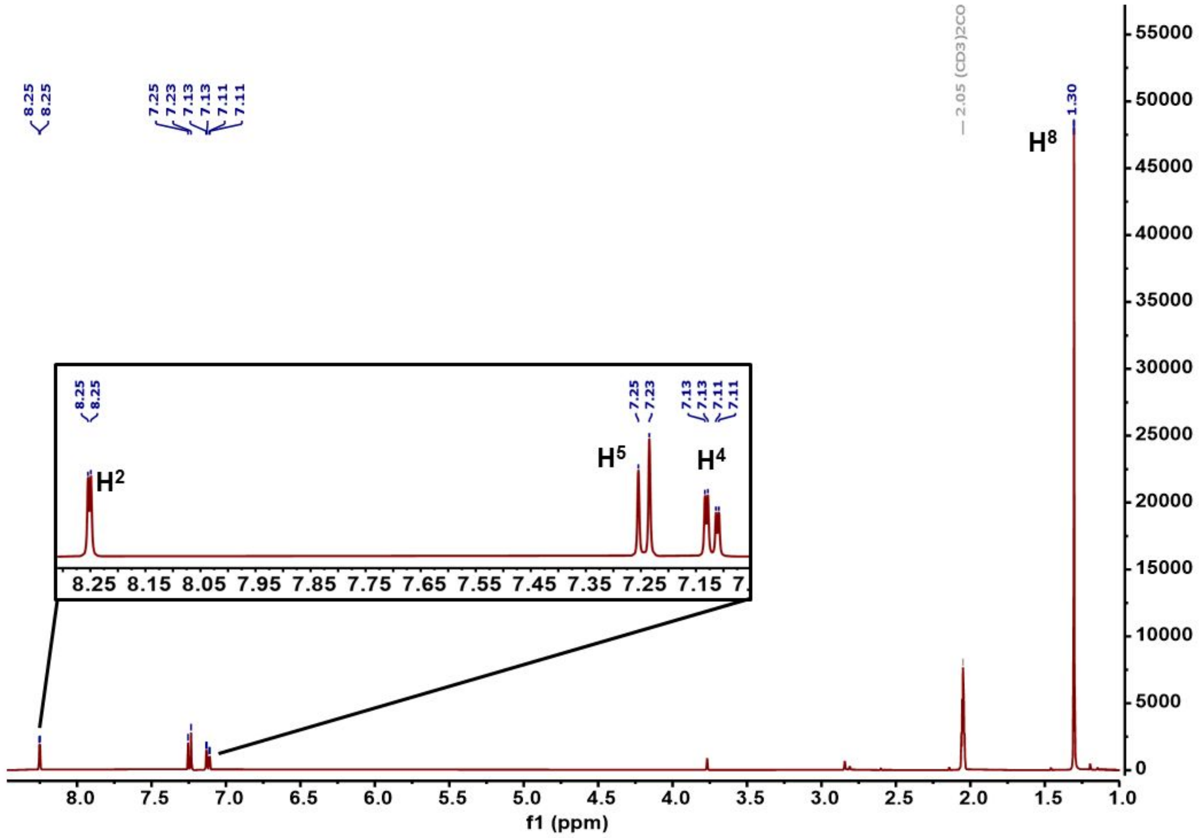

Figure S6. ^{1}H NMR spectrum of **K[Au(C^C)(C≡N)$_2$] 3^K**, $(CD_3)_2CO$.

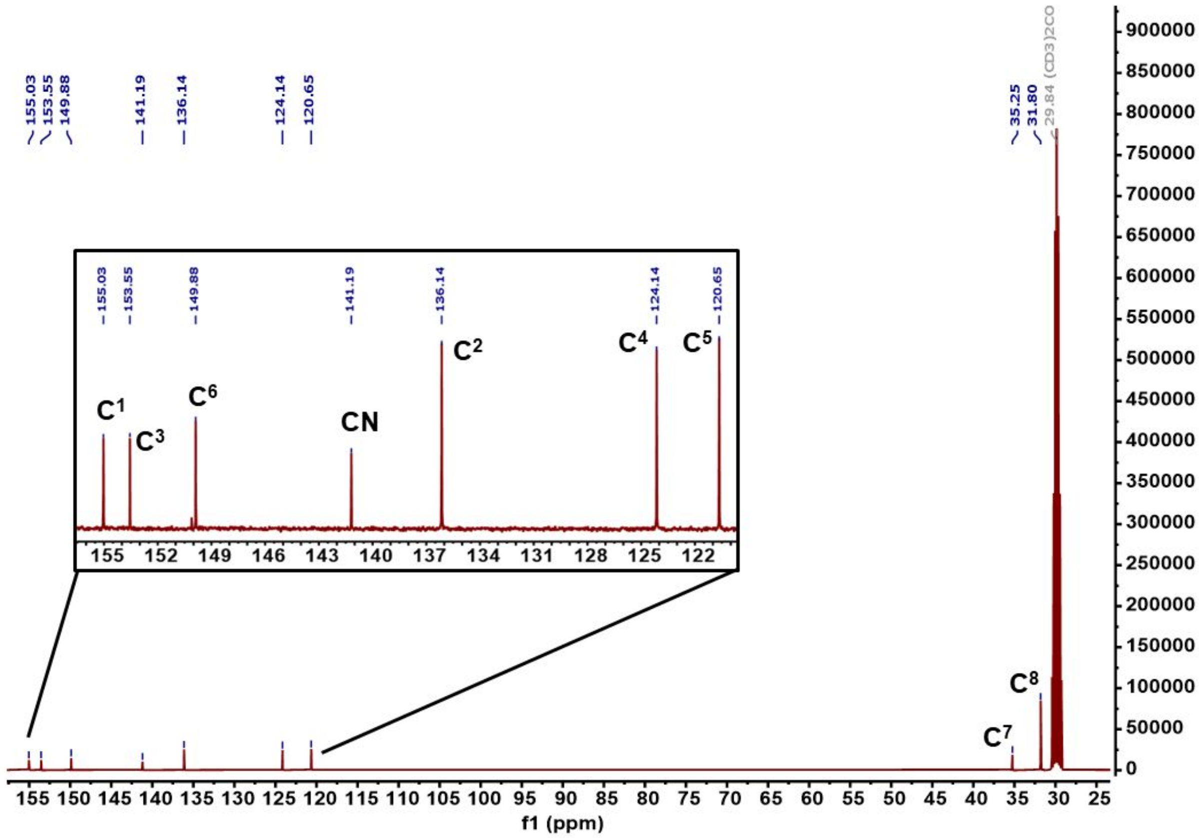

Figure S7. ^{13}C NMR spectrum of **K[Au(C^C)(C≡N)$_2$] 3^K**, $(CD_3)_2CO$.

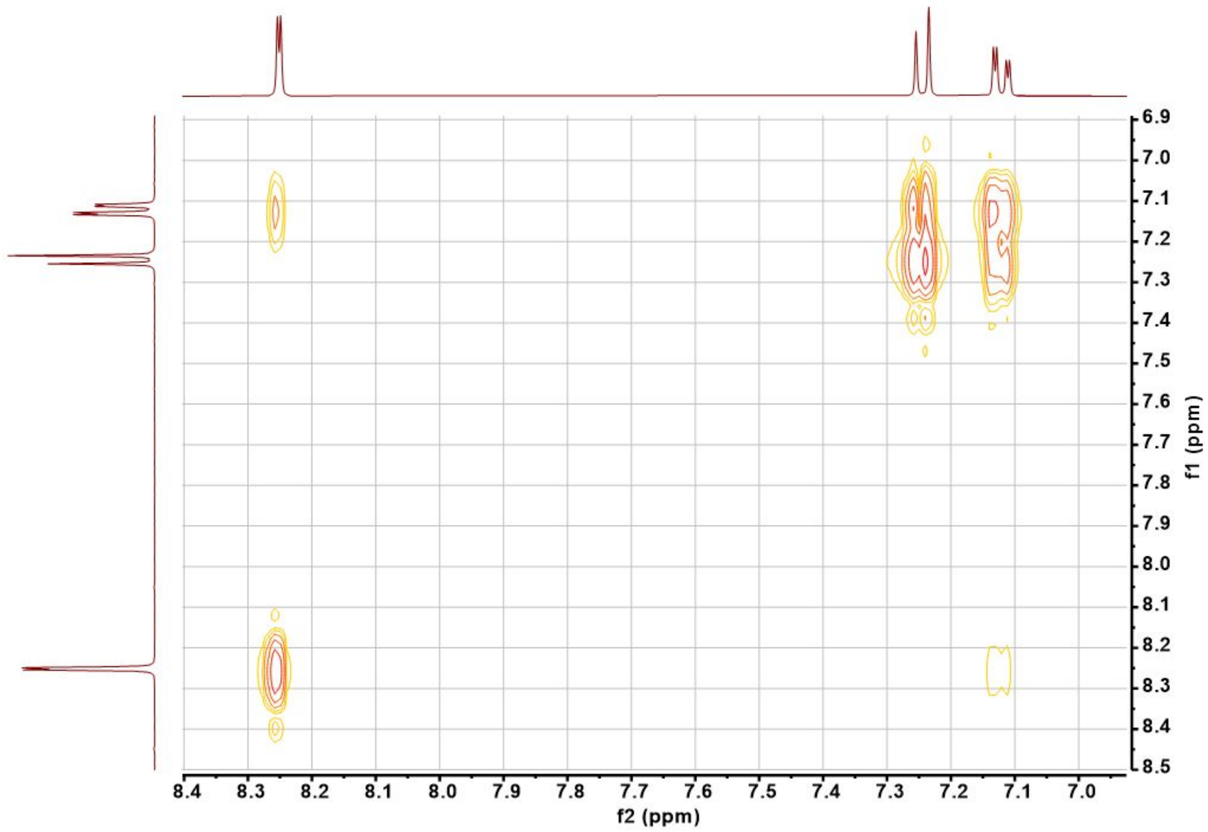

Figure S8. COSY NMR spectrum of **K[Au(C^C)(C≡N)$_2$] 3^K**, $(CD_3)_2CO$.

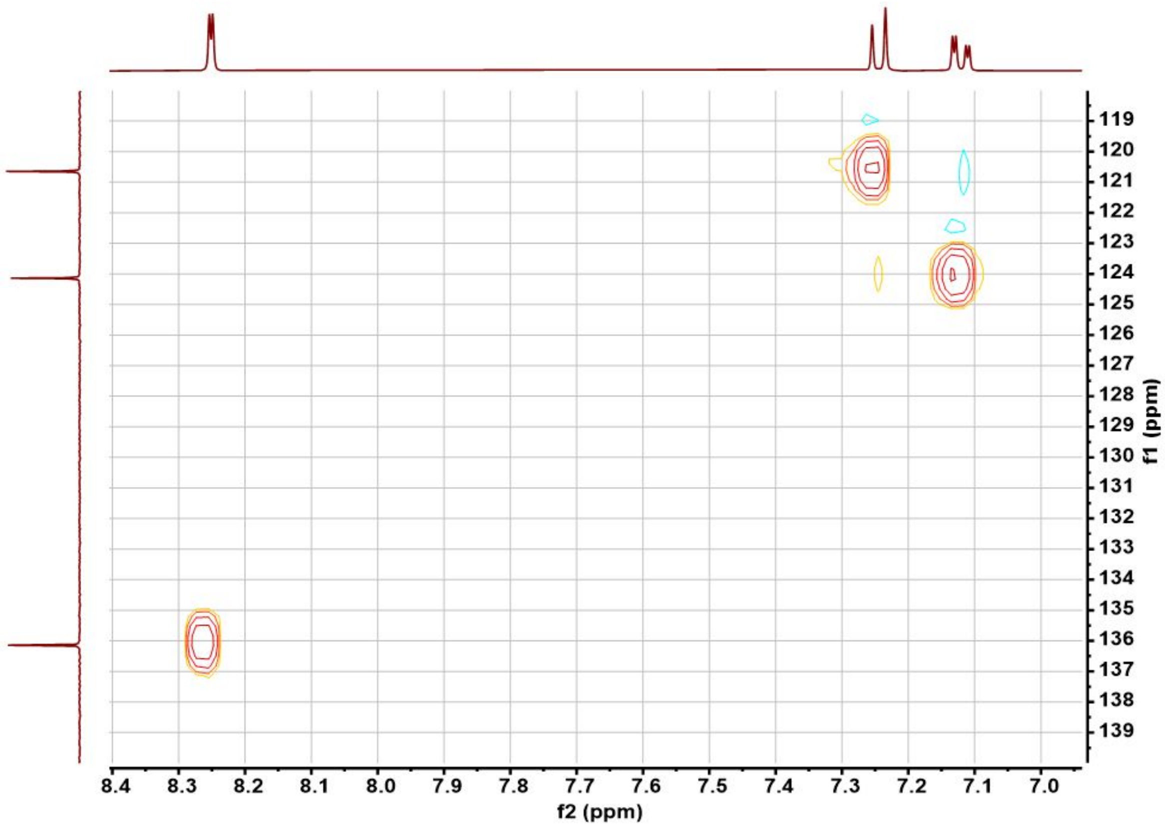

Figure S9. HSQC NMR spectrum of **K[Au(C^C)(C≡N)$_2$] 3^K**, $(CD_3)_2CO$.

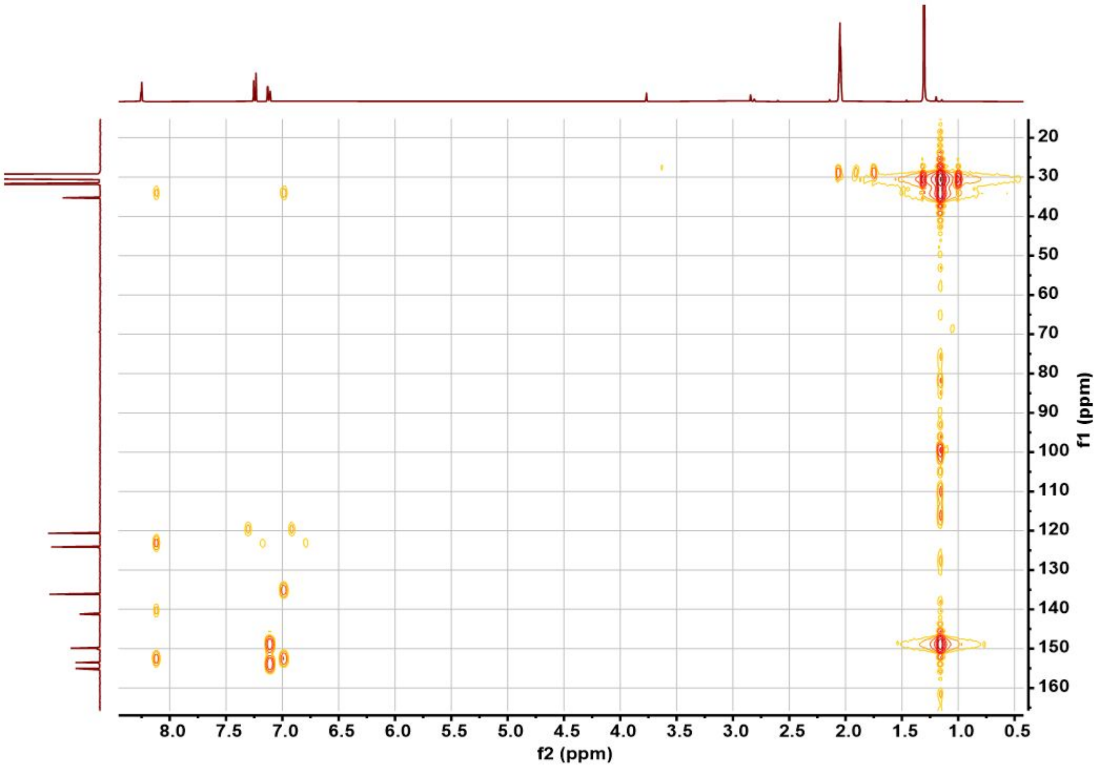

Figure S10. HMBC NMR spectrum of **K[Au(C^C)(C≡N)$_2$] 3^K**, $(CD_3)_2CO$.

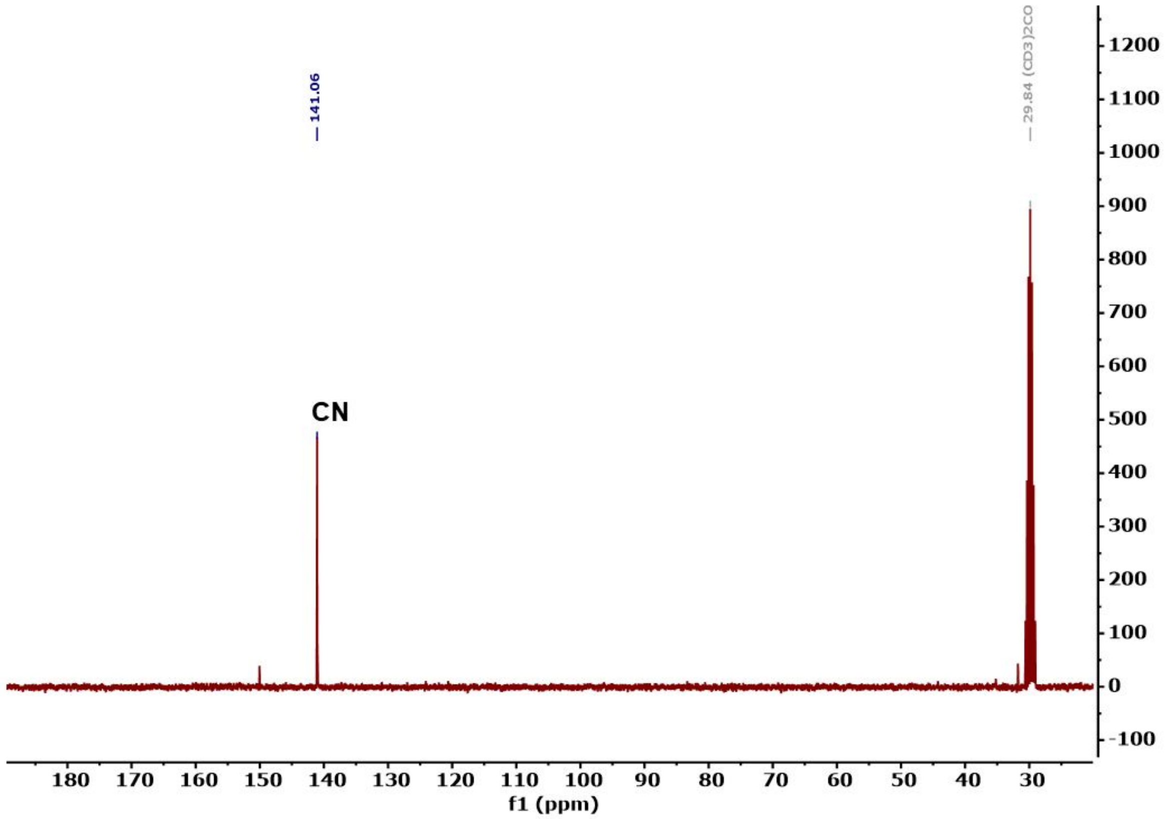

Figure S11. ^{13}C NMR spectrum of **K[Au(C^C)(^{13}C≡N)$_2$] 3^{K}**, $(CD_3)_2CO$.

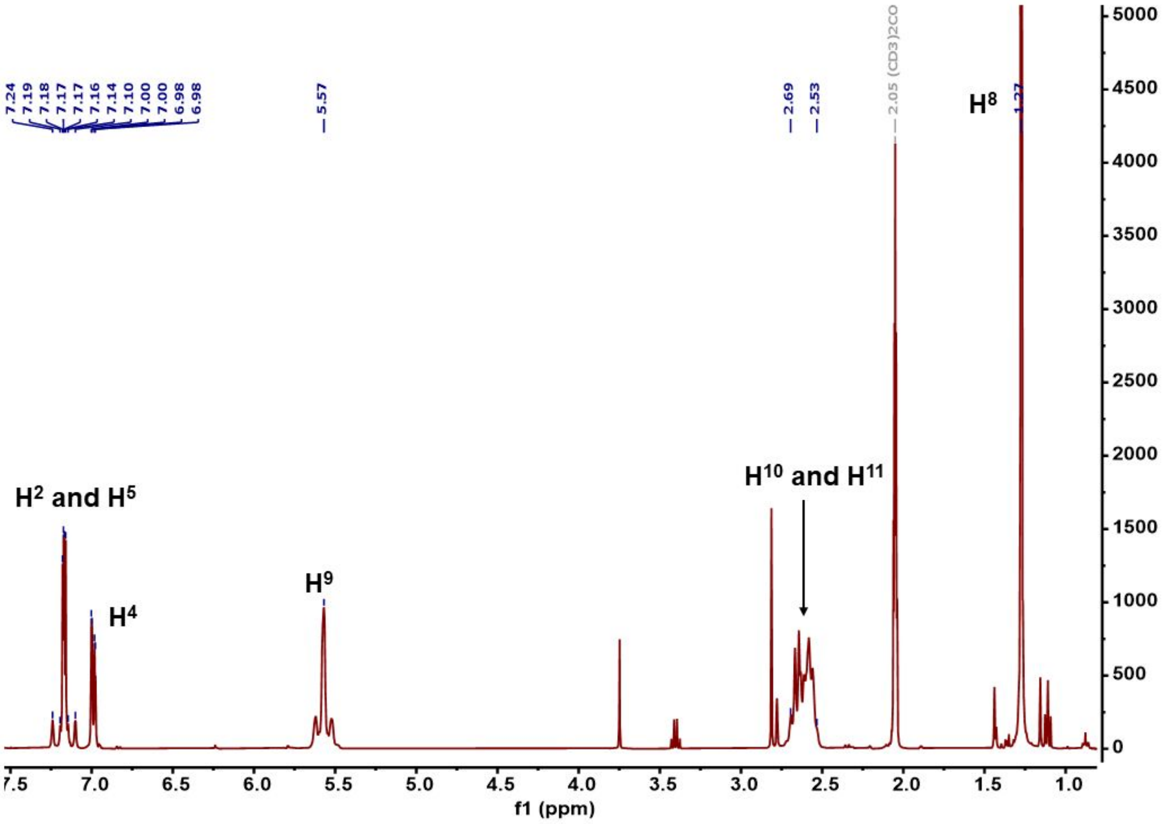

Figure S12. ^{1}H NMR spectrum of **Pt(C^C)(COD) 4,** $(CD_3)_2CO$.

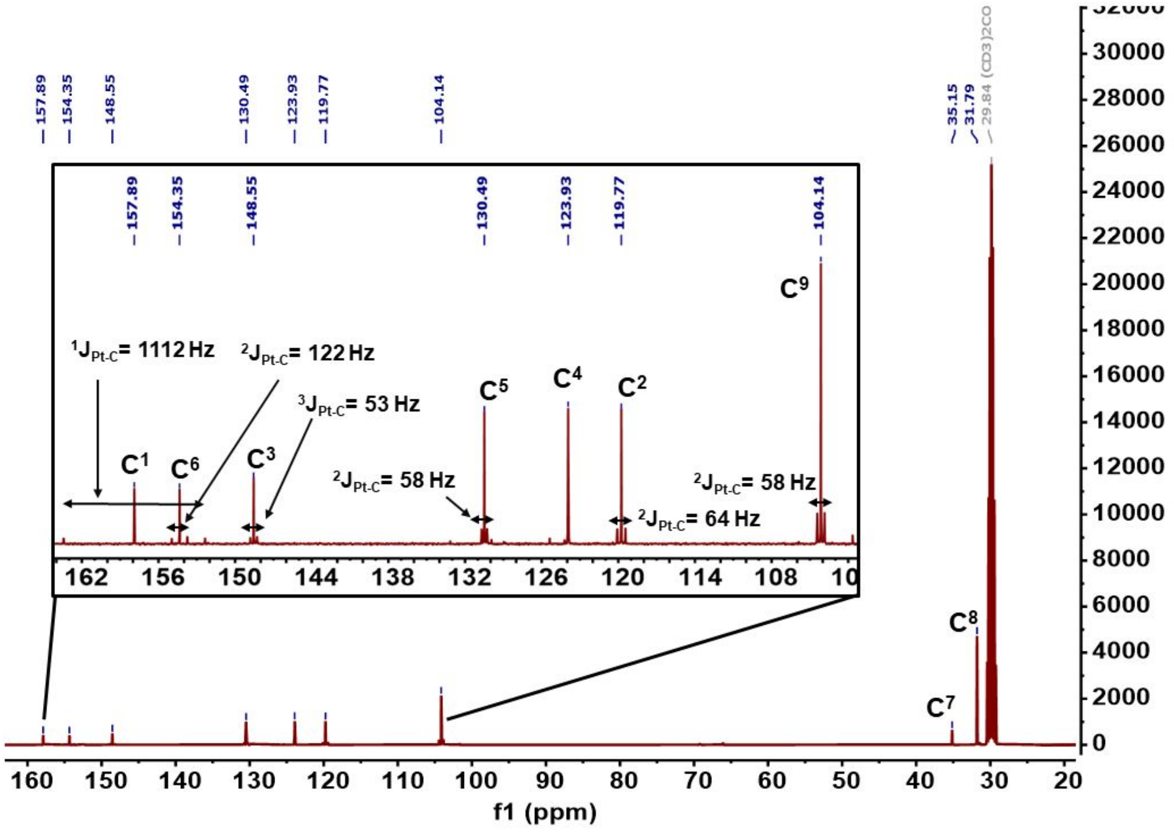

Figure S13. ^{13}C NMR spectrum of **Pt(C^C)(COD) 4**, $(CD_3)_2CO$.

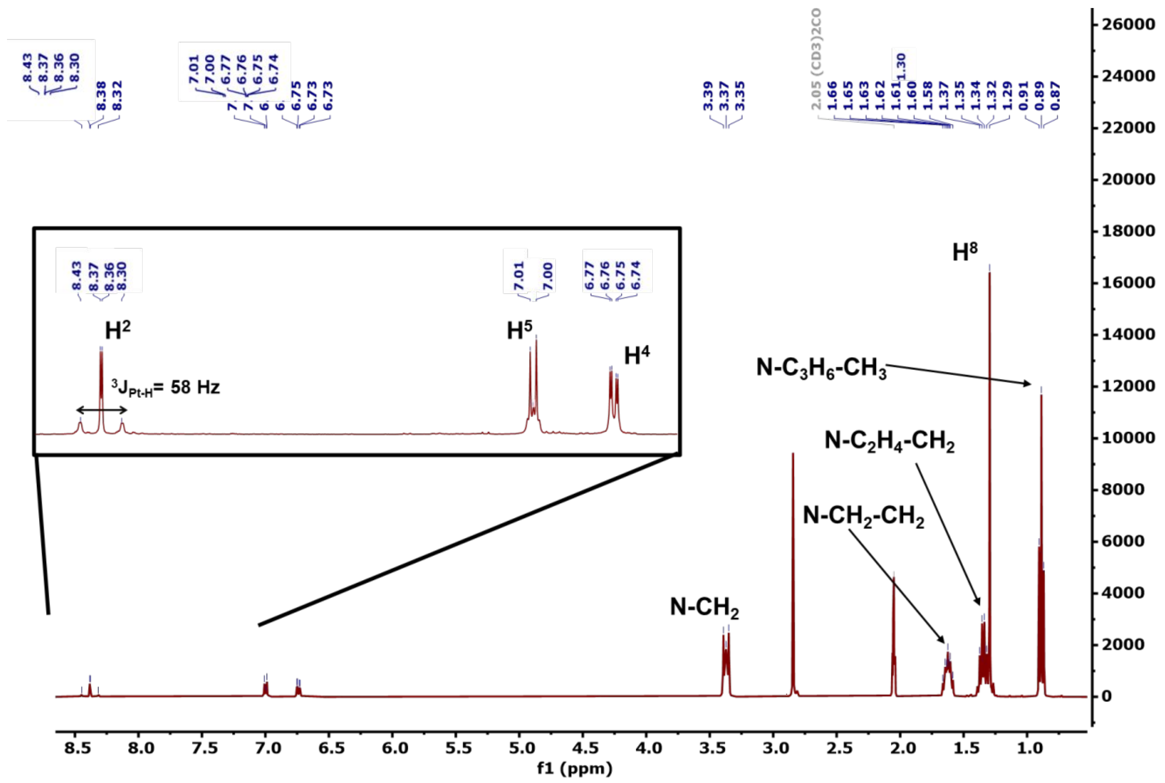

Figure S14. ^{1}H NMR spectrum of **$(NBu_4)_2$[Pt(C^C)(C≡N)$_2$] 5**, $(CD_3)_2CO$.

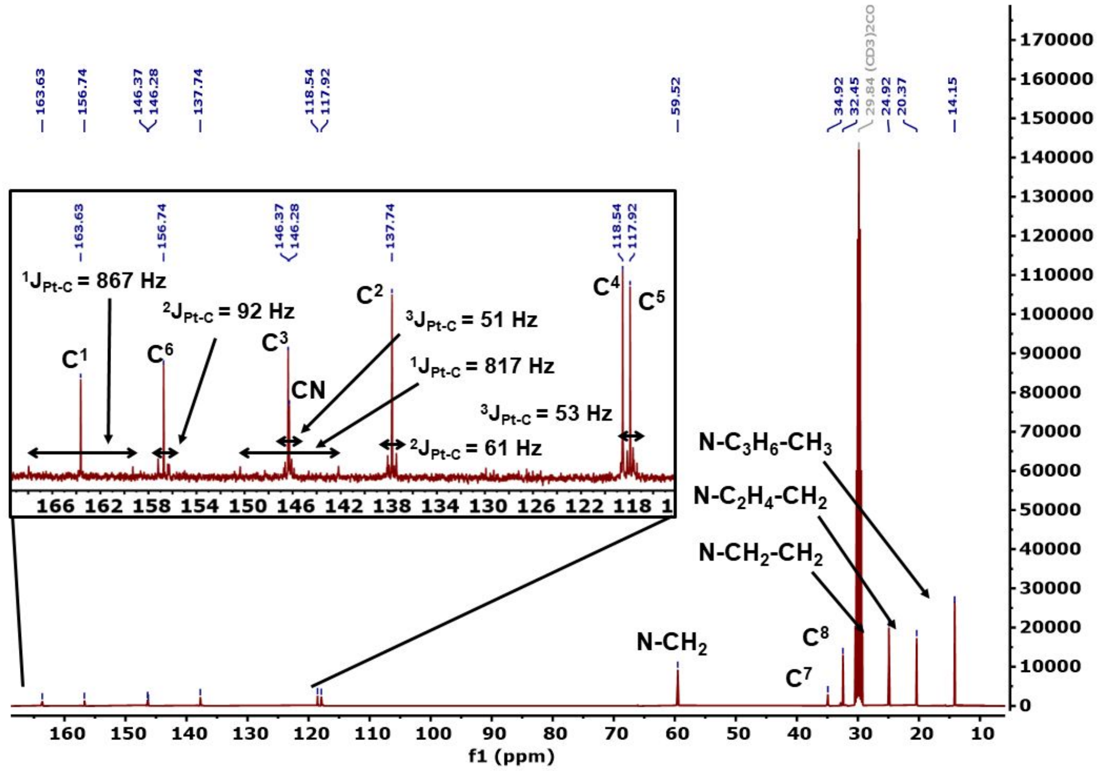

Figure S15. ^{13}C NMR spectrum of **(NBu$_4$)$_2$[Pt(C^C)(C≡N)$_2$] 5**, $(CD_3)_2CO$.

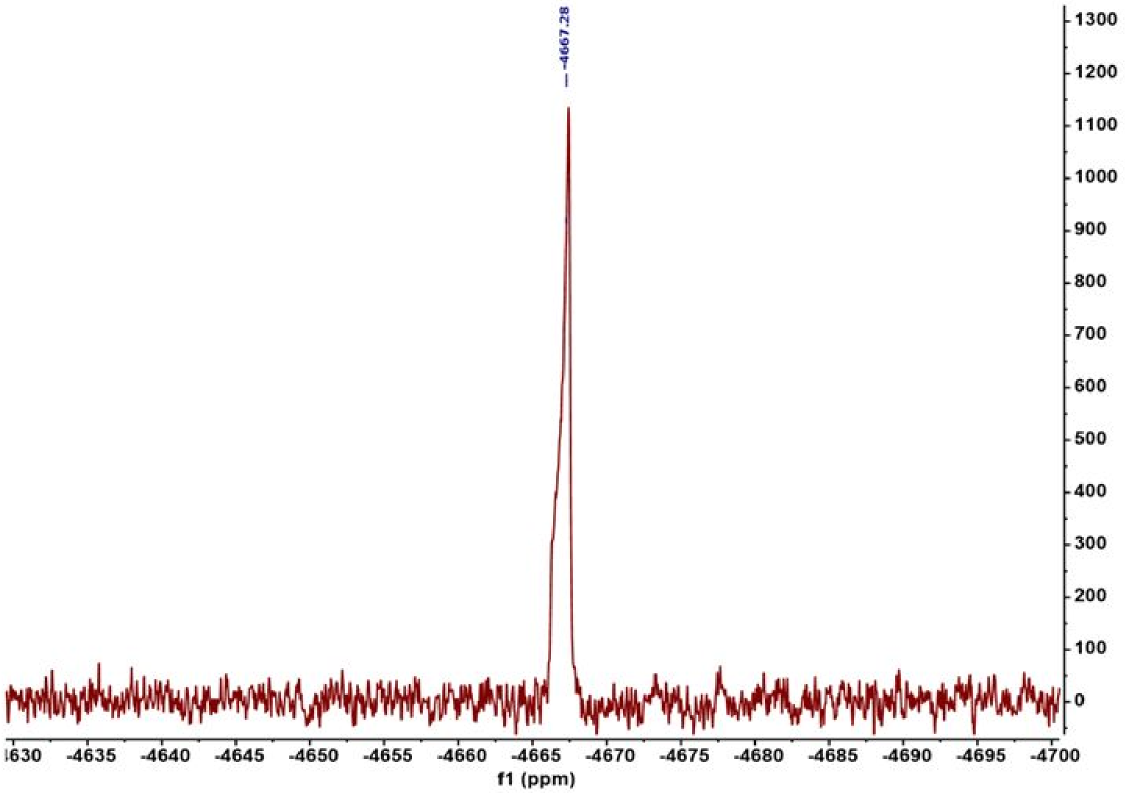

Figure S16. ^{195}Pt NMR spectrum of **(NBu$_4$)$_2$[Pt(C^C)(C≡N)$_2$] 5**, $(CD_3)_2CO$.

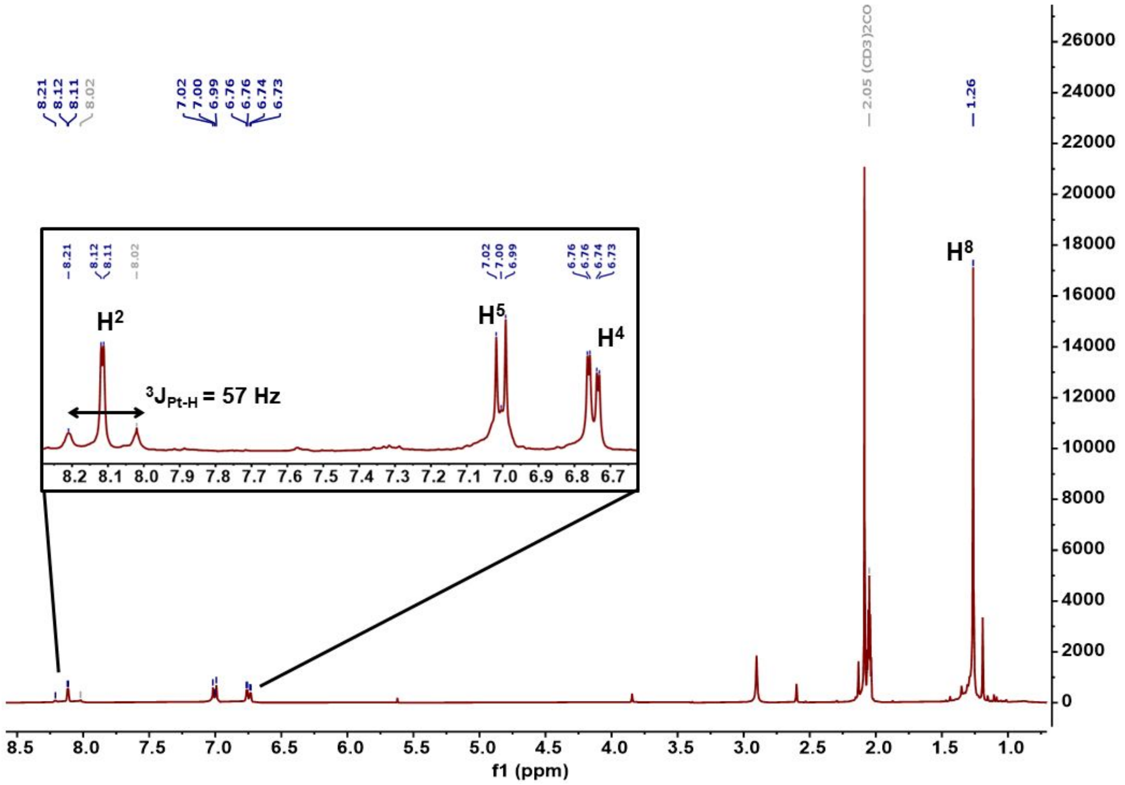

Figure S17. ^{1}H NMR spectrum of **K_2[Pt(C^C)(C≡N)$_2$] 5^K**, $(CD_3)_2CO$.

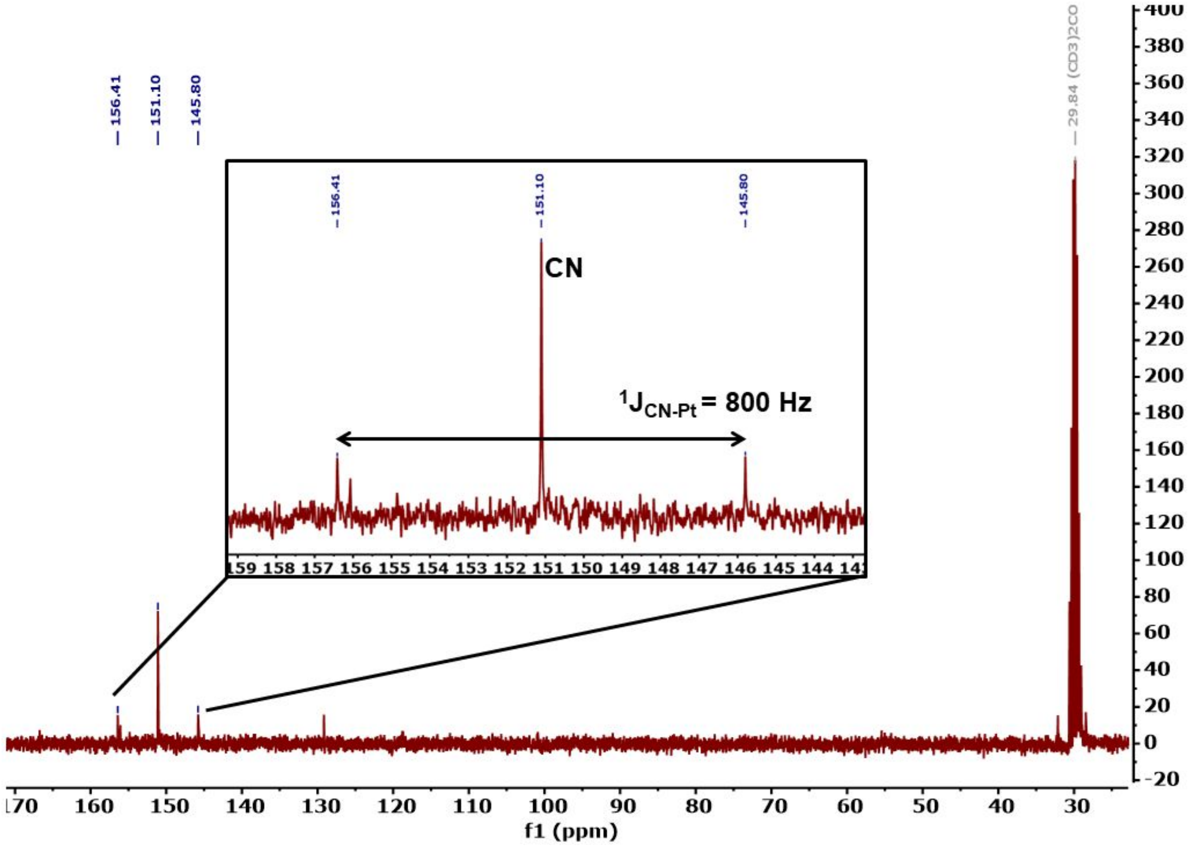

Figure S18. ^{13}C NMR spectrum of **K_2[Pt(C^C) (^{13}C≡N)$_2$] 5^K**, $(CD_3)_2CO$.

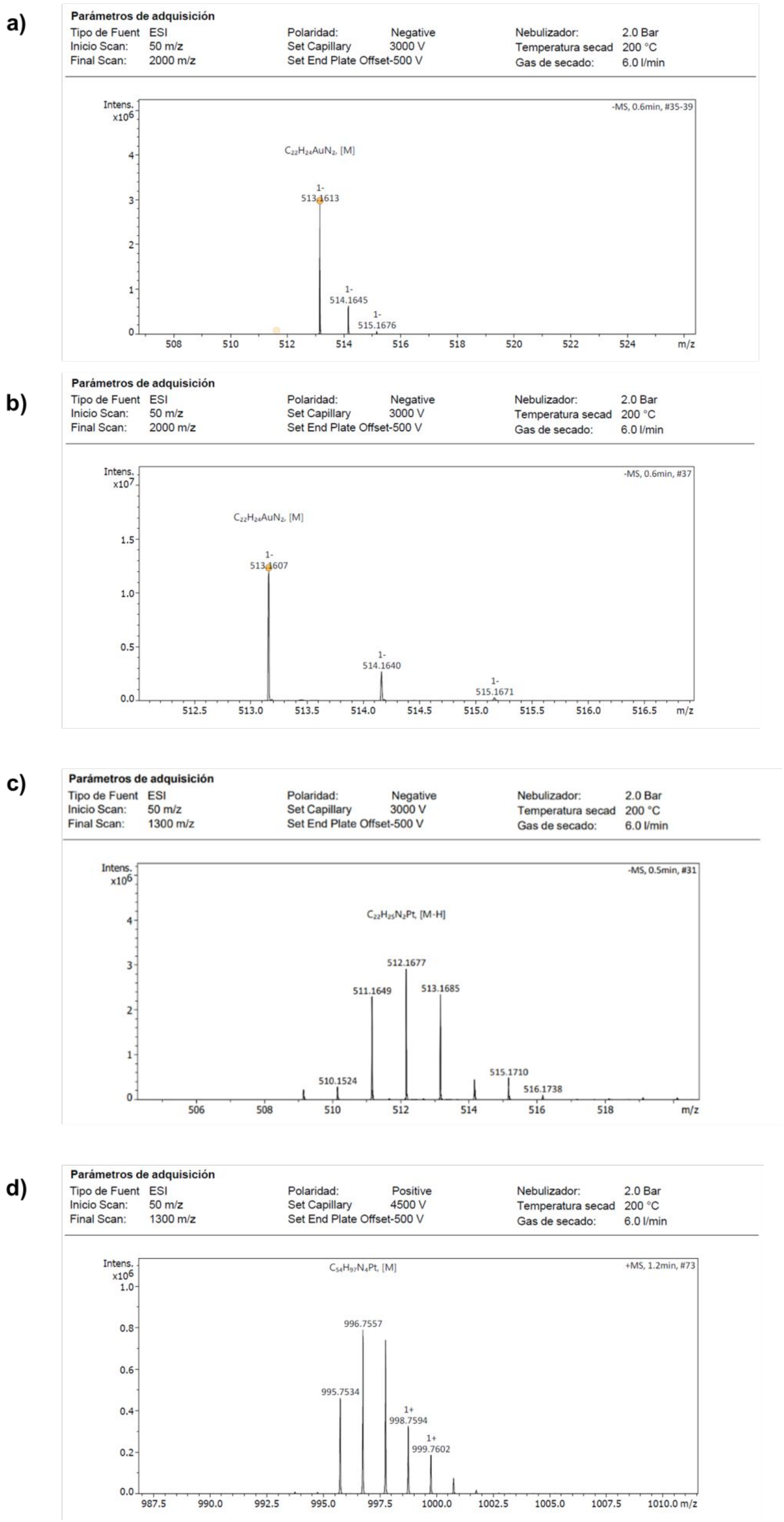

Figure S19. Exact Masses with negative polarity of (a) **3**, (b) **3**$^{\mathbf{K}}$, (c) **5** and with positive polarity of (d) **5.**

2. X-ray crystallography

Crystal data and other details of the structure analysis are presented in Table S1. Suitable crystals for X-ray diffraction studies were obtained by slow diffusion of *n*-hexane into a saturated solution of the respective complexes in CH_2Cl_2 for **1**·0.5*n*-C_6H_{14}, **3**, and **4**. For **1**·0.5*n*-C_6H_{14} and **3**, X-ray intensity data were collected at 100 K on a Bruker Apex Duo CCD diffractometer, and the diffraction frames were integrated and corrected for absorption using the SAINT and SADABS software packages.[S1] For **4**, X-ray intensity data were collected at 100K on an Oxford Diffraction Xcalibur diffractometer, and the diffraction frames were integrated and corrected from absorption by using the CrysAlis RED program.[S2] The radiation used in all cases was graphite monochromated MoK_α (μ = 0.71073 Å). The structures were solved with the ShelXT structure solution program using dual methods and refined by full-matrix least squares on F^2 with olex2.refine 1.5[S3] (**1**·0.5*n*-C_6H_{14} and **3**) or SHELXL (**4**).[S4] All non-hydrogen atoms were assigned anisotropic displacement parameters and refined without positional constraints. All hydrogen atoms were constrained to idealized geometries and assigned isotropic displacement parameters equal to 1.2 or 1.5 times the U_{iso} values of their attached parent atoms. In the case of **3**, data were collected from several samples. Although the appearance of the crystals were excellent, problems always arise in the form of high *R* values and excessively high or even NPD thermal parameters. The data collection corresponding to this resolution, which proved to be the best, was carried out on a nearly two-dimensional sheet-shaped crystal. The data, while not entirely good, are acceptable. A rotational disorder was modelled in one of the *tert*-butyl groups with occupations of 0.666/0.333. Refinement of these models lead to the parameters listed in table S1.

CCDC numbers 2500672-2500674 contain the supplementary crystallographic data for the structures reported here.

Table S1. Crystallographic data of 1, 3 and 4			
**Parameters**	**1**	**3**	**4**
**CCDC number**	2500672	2500673	2500674
**Empirical Formula**	$C_{28}H_{42}Sn$	$C_{38}H_{60}AuN_3$	$C_{31}H_{43}Pt$
**Formula Weight**	497.356	755.887	610.74
**Temperature**	100.00 (K)	100.00 (K)	100(2) K
**Wavelength**	0.71073 Å	0.71073 Å	0.71073 Å
**Crystal System**	Triclinic	Monoclinic	Orthorhombic
**Space Group**	*P-1*	$P2_1/n$	*Pbca*
**Unit Cell Dimensions**	*a* = 8.7877(16) Å<br>α = 111.493(5)°<br>*b* = 10.728(12) Å<br>β = 96.374(6)°<br>*c* = 14.601(4) Å<br>γ = 90.157(6)°	*a* = 9.4313(7) Å<br>α = 90°<br>*b* = 15.3027(12) Å<br>β = 90.217(3)°<br>*c* = 25.259(2) Å<br>γ = 90°	*a* = 17.21402(18) Å<br>α = 90°<br>*b* = 10.54496(10) Å<br>β = 90°<br>*c* = 29.1856(3) Å<br>γ = 90°
**Volume**	1271.4(5)	3645.4(5) $Å^3$	5297.80(9) $Å^3$
**Z**	Z 2 Z' 1	4	8
**Density (calculated)**	1.299 g/cm^3	1.377 g/cm^3	1.531 Mg/m^3
**Absorption coeffcient**	1.016 mm^{-1}	4.064 mm^{-1}	5.313 mm^{-1}
**F(000)**	518.991	1547.3	2456
**Crystal Size**	0.35 x 0.24 x 0.13 mm^3	0.33 x 0.19 x 0.01 mm^3	0.380 x 0.240 x 0.150 mm^3
**2 Theta Range for data collection**	4.08 to 56.94	4.18 to 56.82	2.661 to 29.523°.
**Index ranges**	-11<=h<=11<br>-14<=k<=14<br>- 19<=l<=19	-12<=h<=12<br>-20<=k<=20<br>- 33<=l<=33	-23<=h<=23<br>-14<=k<=13<br>-37<=l<=40
**Reflections Collected**	39485	224612	87347
**Independent Reflections**	6391 [R(int) = 0.0387]	9147 [R_{int} = 0.0970, R_{sigma} = 0.0558]	6953 [R(int) = 0.0407]
**Completeness**	99.98 % (theta=28.47)	99.95 % (theta=28.41)	99.9 % (theta=25.000)
**Refinement Method**	Full-matrix least-squares on F^2	Full-matrix least-squares on F^2	Full-matrix least-squares on F^2
**Data / Restraints / Parameters**	6391/0/270	9147/48/444	6953 / 0 / 312
**Goodness-of-fit on F^2**	1.0291	1.005	1.058
**Final R inidces [I>2sigma(I)]**	R_1 = 0.0245, wR_2 = 0.0576	R_1 = 0.0701, wR_2 = 0.1543	R1 = 0.0234, wR2 = 0.0468
**R índices (all data)**	R_1 = 0.0270, wR_2 = 0.0587	R_1 = 0.0813, wR_2 = 0.1600	R1 = 0.0324, wR2 = 0.0499
**Largest diff. Peak and hole**	0.5650 and -0.8934 e.$Å^{-3}$	3.44 and -4.89 e.$Å^{-3}$	1.489 and -0.998 e.$Å^{-3}$

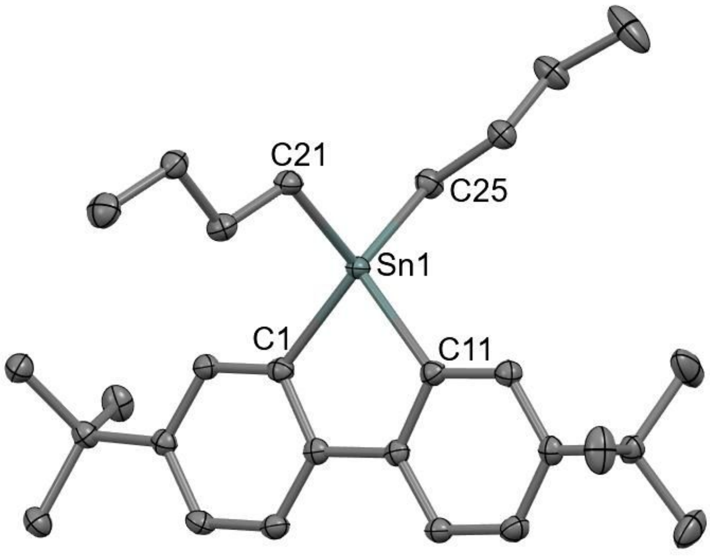

Figure S20. Molecular structure [selected bond distances (Å) and angles (°)] for **1**: Sn-C1 2.133(2), Sn-C11 2.138(2), Sn-C21 2.152(2), Sn-C25 2.144(2), C1-Sn-C11 83.73(7), C1-Sn-C21 114.59(7), C11-Sn-C25 114.43(7), C21-Sn-C25 105.89(7).

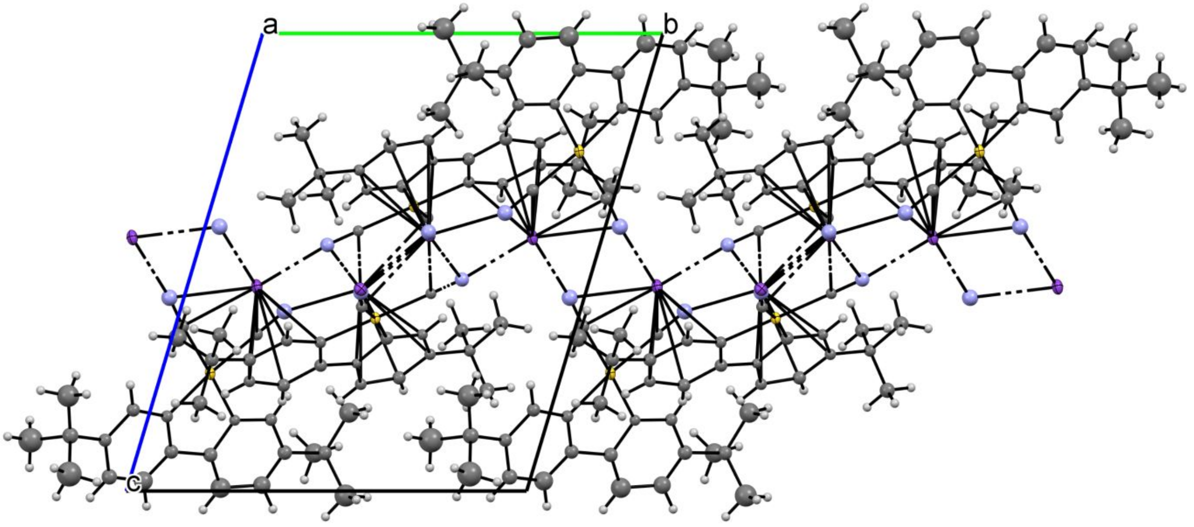

Figure S21. View of the packing along the *bc* face in the X-ray structure of $\mathbf{3^K}$.

S3. Photophysical Properties

The UV–vis absorption spectra were measured with a Hewlett-Packard 8453 spectrophotometer. Excitation and emission spectra were obtained with an Edinburgh FLS 1000 spectrofluorimeter. Lifetime measurements were measured with an Edinburgh FLS 1000 spectrofluorimeter with a μF^2 pulse lamp (power: 100 W, fuse: 3.15 Amp A/S) or with a Datastation HUB-B with a nanoLED controller using the technique "time correlated single photon counting" (TCSPC). The decay data were analyzed using the software DAS6 (Jobin Yvon-Horiba). The absolute quantum yields were determined with a Hamamatsu Absolute PL Quantum Yield Measurement System. The estimated uncertainty is ~5% or better. Polymeric sample preparation: PMMA (polymethyl methacrylate) was purchased from commercial sources. Thin films were prepared by spin/drop casting from a solution of the corresponding complex and the amount of polymer to reach the desired concentration in each case. Films were then placed under vacuum for 10 minutes to remove residual solvent and kept in N_2 atmosphere for several days before measuring.

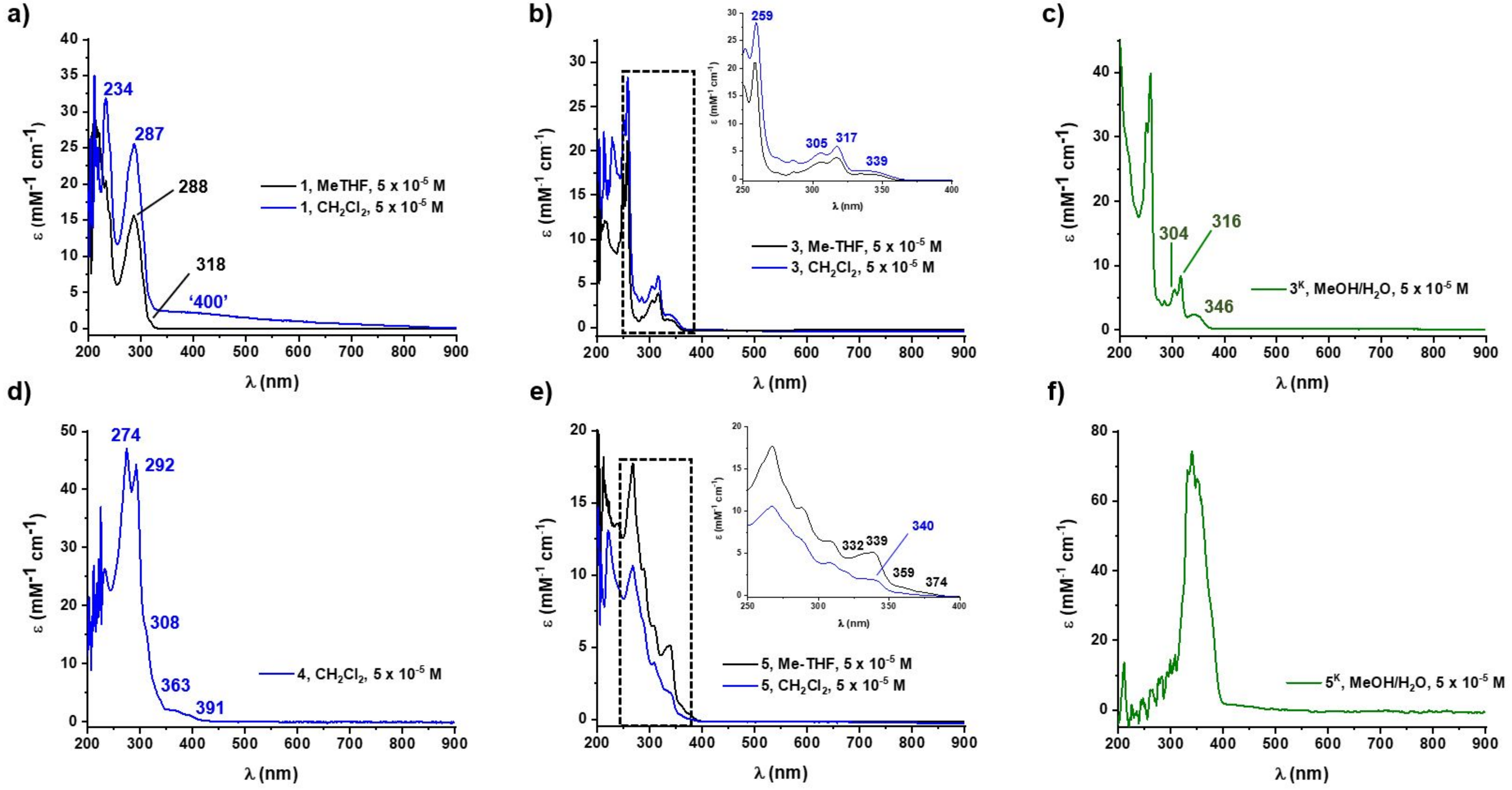

Figure S22. UV-Vis absorption spectra of (a) **1** in Me-THF and CH_2Cl_2 5 × 10^{-5} M solution. (b) **3** in Me-THF and CH_2Cl_2 5 × 10^{-5} M solution. (c) $\mathbf{3^K}$ in MeOH:H_2O 1:9, 5 × 10^{-5} M solution. (d) **4** in CH_2Cl_2 5 × 10^{-5} M solution. (e) **5** in Me-THF and CH_2Cl_2 5 × 10^{-5} M solution. (f) $\mathbf{5^K}$ in MeOH:H_2O 1:9, 5 × 10^{-5} M solution.

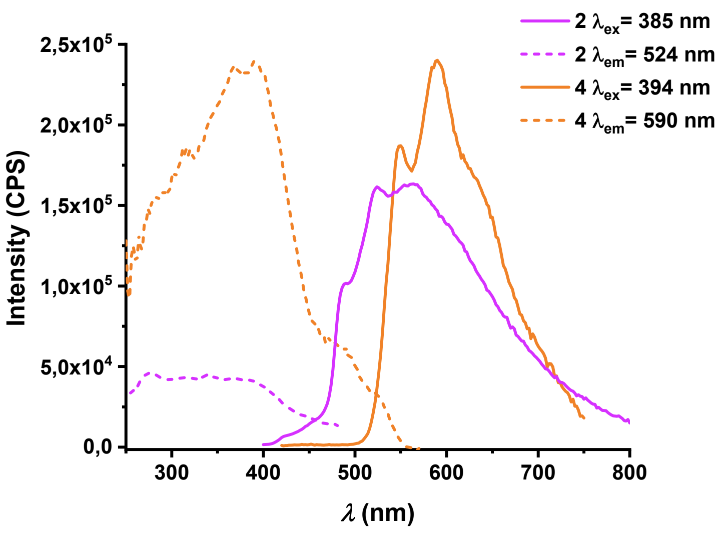

Figure S23. Excitation and Emission spectra of **2** and **4** in the solid state.

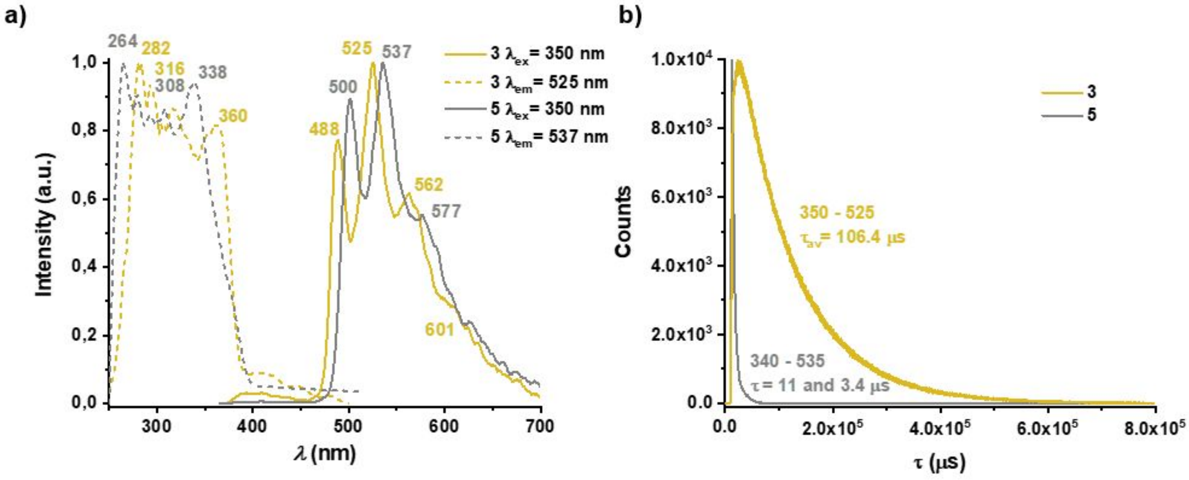

Figure S24. (a) Excitation and Emission spectra of **3** (solid) and **5** (oil). (b) Decay curves of the emission bands for the same samples.

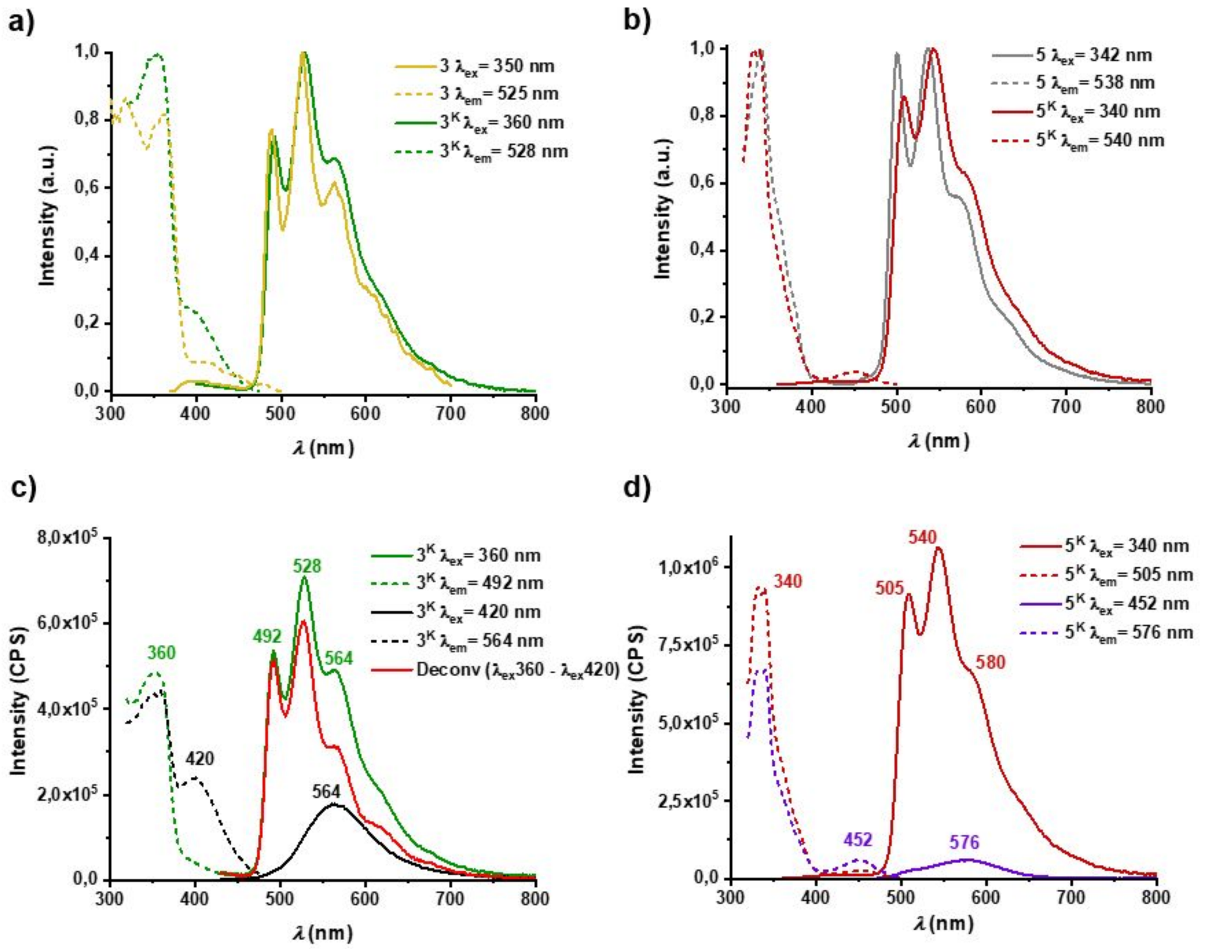

Figure S25. (a) Excitation and Emission spectra of **3** and **3^K** in the solid state. (b) Excitation and Emission spectra of **5** as a film and **5^K** in the solid state. (c) Excitation, Emission spectra and deconvolution of **3^K** in the solid state (d) Excitation, Emission spectra and deconvolution of **5^K** in the solid state.

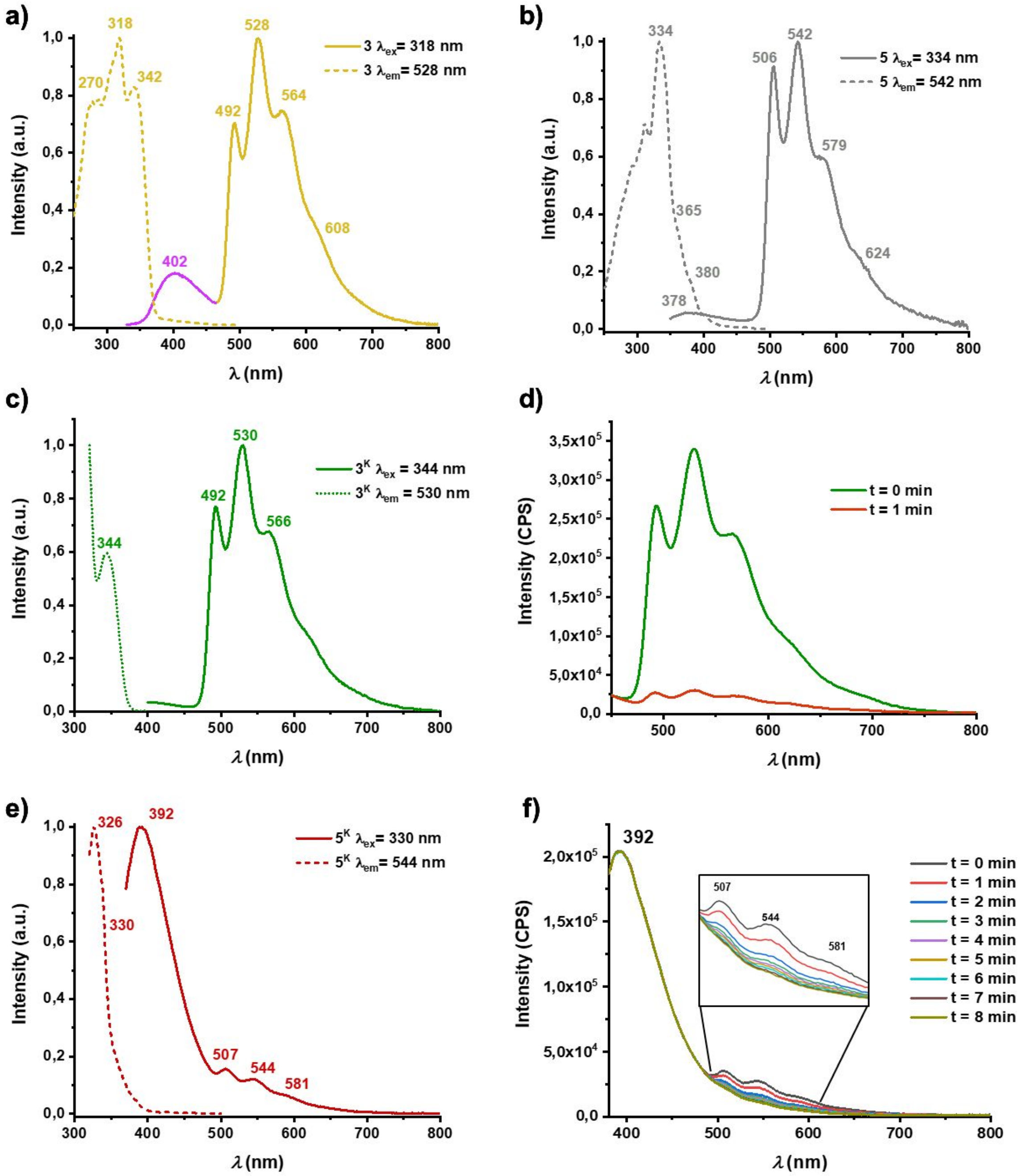

Figure S26. (a) Excitation and Emission spectra of **3** in CH_2Cl_2 (5×10^{-4} M). (b) Excitation and Emission spectra of **5** in CH_2Cl_2 (5×10^{-4} M). (c) Excitation and Emission spectra of $\mathbf{3^K}$ in MeOH:H_2O 1:9 (5×10^{-4} M). (d) Quenching by oxygen of the emission band of $\mathbf{3^K}$ in MeOH:H_2O 1:9 (5×10^{-4} M). (e) Excitation and Emission spectra of $\mathbf{5^K}$ in MeOH:H_2O 1:9 (5×10^{-4} M). (f) Quenching by oxygen of the emission band of $\mathbf{5^K}$ in MeOH:H_2O 1:9 (5×10^{-4} M).

S4. Theoretical Calculations

Calculations were carried out with the Gaussian 16 package[S5] for **1-5^K**. The starting point for geometry optimizations was the molecular geometry obtained through X-ray diffraction analysis for all complexes except **5** and **5^K**. A study of functionals and basis sets was done to determine which one is better. The functionals tested were the B3LYP[S6] with Becke-Johnson D3BJ correction[S7], M06L functional[S8] and M062X functional[S9]; the best results were obtained with M06L functional. The basis sets tested for Pt atoms were LANL2DZ effective core potential and the SDD basis set[S10] and its associated effective core potential, the best results were obtained with the SDD basis set except for **5$^-$** where the best basis set was the LANL2DZ. For the other atoms the basis set tried were: 6-31G(d,p), 6-311G(d,p) and 6-311++G(d,p), the best results were obtained using the 6-311G(d,p) basis sets.[S11] No negative frequency was found in the vibrational frequency analysis of the final equilibrium geometries. TD-DFT was used for excited-state calculations, employing the M062X functional, above basis sets, and the polarized continuum method approach[S12] (PCM) implemented in the Gaussian 16 software, in presence of dichloromethane for all complexes. The predicted emission wavelengths were obtained by energy difference between the triplet state at its optimized geometry and singlet state at the triplet geometry. The results were visualized with GaussView 6.0. Overlap populations between molecular fragments were calculated using the GaussSum 3.0 software.[S13]

Theoretical Calculations of Sn(C^C)^{n}Bu$_2$ (1)

Table S2. Selected parameters of DFT optimized geometries for ground state and triplet state in gas phase.

Distance/angle	Single crystal	S_0	T_1
Sn-C(C^C)	2.1332(18)<br>2.1382(18)	2.16216<br>2.15747	2.15754<br>2.15361
Sn-C(^{n}Bu)	2.1517(19)<br>2.1445(19)	2.18210<br>2.18389	2.18461<br>2.18671
C(C^C)-Sn-C(C^C)	83.74(7)	82.918	82.735
C(C^C)-Sn-C(^{n}Bu)	114.59(7)<br>114.43(7)	113.155<br>112.145	113.497<br>112.552
C(^{n}Bu)-Sn-C(^{n}Bu)	105.88(7)	112.130	111.374

Table S3. Selected frontier Molecular Orbitals and Composition (%) of Frontier MOs in terms of ligands and metals for ground state in gas phase.

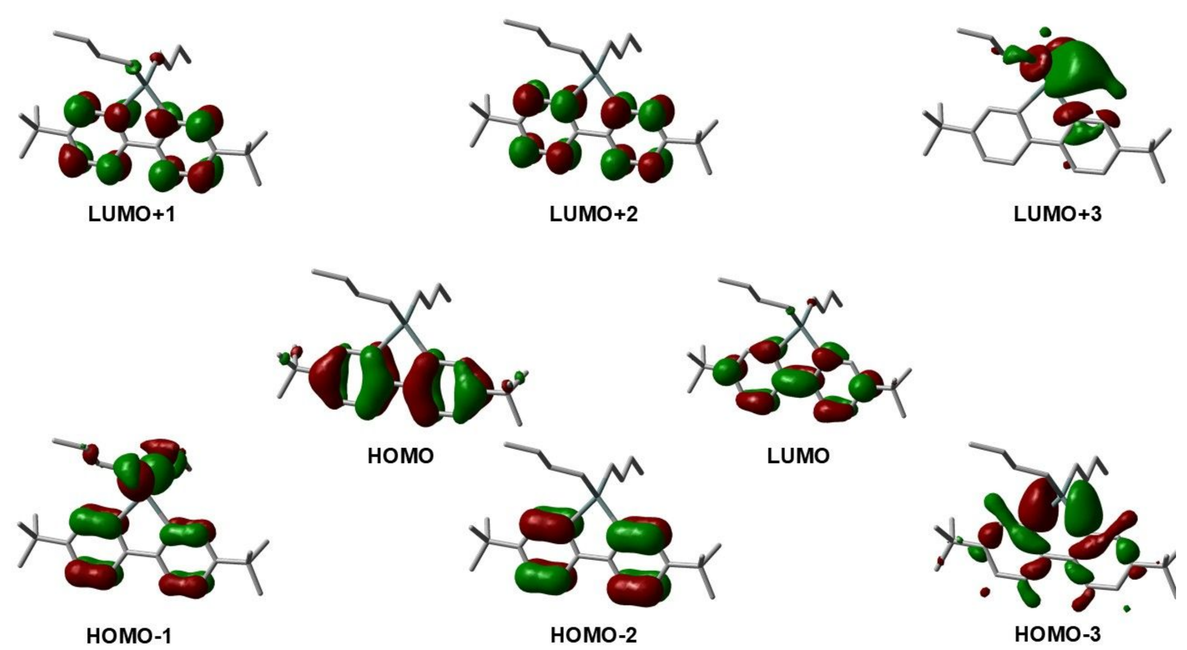

Orbital	Energy (eV)	Sn	C^C	^{n}Bu
LUMO+5	0.41	42	33	26
LUMO+4	0.39	4	93	4
LUMO+3	0.19	51	22	27
LUMO+2	-0.40	0	100	0
LUMO+1	-1.12	6	92	2
LUMO	-1.47	4	94	2
HOMO	-5.15	0	100	0
HOMO-1	-5.96	5	56	39
HOMO-2	-6.07	0	99	1
HOMO-3	-6.16	19	79	2
HOMO-4	-6.55	8	66	26
HOMO-5	-6.58	19	45	37

**Table S4. Selected vertical excitation energies singlets (S_n) and first triplet state (T_1) computed by TD-DFT (Gas Phase) with the orbitals involved.**				
**State**	**λ/nm**	***f***	**Transition (% Contribution)**	**Character**
T_1	371	/	HOMO → LUMO (85%)	IL
S_1	272	0.3339	HOMO → LUMO (81%)<br>HOMO → L+1 (13%)	IL
S_2	262	0.2131	HOMO → L+1 (65%)<br>H-2 → LUMO (16%)<br>HOMO → LUMO (14%)	IL
S_3	239	0.0037	H-1 → LUMO (39%)<br>HOMO → L+3 (34%)<br>H-4 → LUMO (11%)	IL/L'LCT/LMCT
S_6	217	0.1868	H-2 → LUMO (74%)<br>HOMO → L+1 (19%)	IL
S_8	206	0.2577	H-1 → L+1 (40%)<br>H-1 → LUMO (28%)	IL/L'LCT

**Table S5. Plots and composition (%) of the frontier MOs and spin density of the first triplet state in gas phase.**		
**SOMO**	**SOMO-1**	**Spin Density**
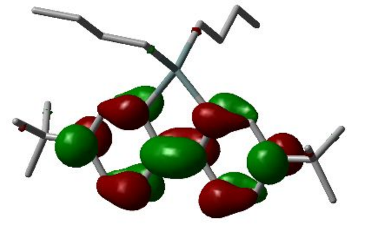	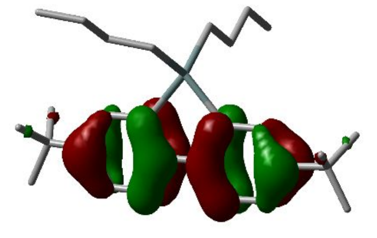	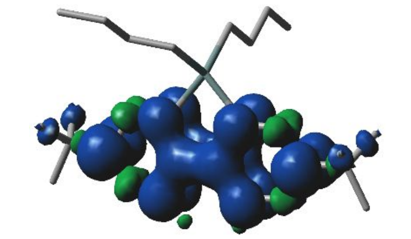
Sn 2%, C^C 96%, ^{n}Bu 2%	Sn 0%, C^C 100%, ^{n}Bu 0%	Sn -0.0178

Table S6. Selected parameters of DFT optimized geometries for ground state and triplet state in CH_2Cl_2 solution.			
**Distance/angle**	**Single crystal**	**S_0**	**T_1**
Sn-C(C^C)	2.1332(18)<br>2.1382(18)	2.16713<br>2.16397	2.16250<br>2.16027
Sn-C(^{n}Bu)	2.1517(19)<br>2.1445(19)	2.17959<br>2.17953	2.18220<br>2.18278
C(C^C)-Sn-C(C^C)	83.74(7)	82.546	82.420
C(C^C)-Sn-C(^{n}Bu)	114.59(7)<br>114.43(7)	112.724<br>112.984	112.841<br>113.243
C(^{n}Bu)-Sn-C(^{n}Bu)	105.88(7)	112.849	112.095

Table S7. Selected frontier Molecular Orbitals and Composition (%) of Frontier MOs in terms of ligands and metals for ground state in CH_2Cl_2 solution.

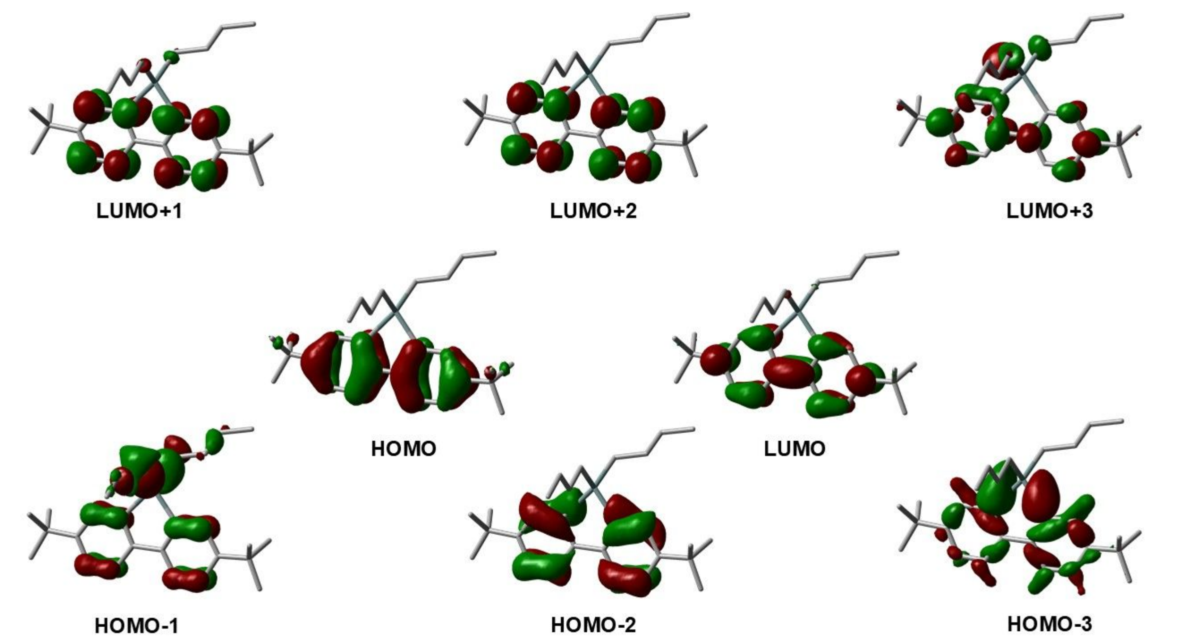

Orbital	Energy (eV)	Sn	C^C	^{n}Bu
LUMO+5	0.47	47	28	24
LUMO+4	0.28	33	46	20
LUMO+3	0.27	16	74	10
LUMO+2	-0.52	0	100	0
LUMO+1	-1.24	6	92	2
LUMO	-1.59	3	95	2
HOMO	-5.28	0	100	0
HOMO-1	-6.01	7	45	48
HOMO-2	-6.20	4	95	1
HOMO-3	-6.20	14	84	2
HOMO-4	-6.58	19	43	38
HOMO-5	-6.62	8	67	24

**Table S8. Selected vertical excitation energies singlets (S_n) and first triplet state (T_1) computed by TD-DFT (CH_2Cl_2 Solution) with the orbitals involved.**				
**State**	**λ/nm**	***f***	**Transition (% Contribution)**	**Character**
T_1	370	/	HOMO → LUMO (86%)	IL
S_1	273	0.5752	HOMO → LUMO (91%)	IL
S_2	263	0.1463	HOMO → L+1 (74%)<br>H-2 → LUMO (15%)	IL
S_3	241	0.0003	H-3 → LUMO (90%)	IL
S_6	218	0.2023	H-2 → LUMO (77%)<br>HOMO → L+1 (17%)	IL
S_8	209	0.4262	H-1 → L+1 (36%)<br>H-1 → LUMO (34%)<br>HOMO → L+2 (13%)	IL/L'LCT
S_{10}	201	0.1348	H-1 → L+1 (29%)<br>HOMO → L+2 (21%)<br>H-1 → LUMO (19%)	IL/L'LCT

**Table S9. Plots and composition (%) of the frontier MOs and spin density of the first triplet state in CH_2Cl_2 solution.**		
**SOMO**	**SOMO-1**	**Spin Density**
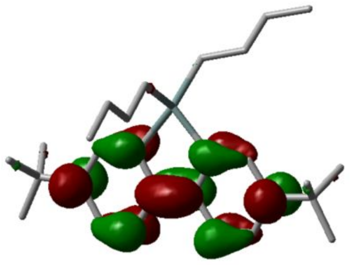	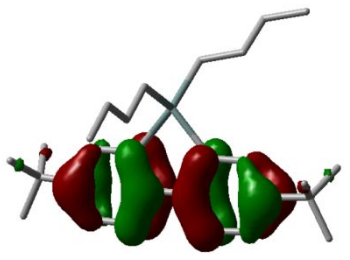	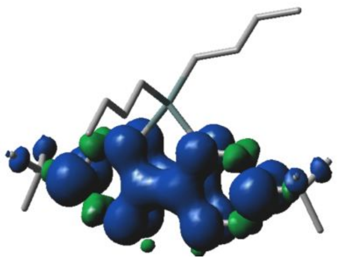
Sn 2%, C^C 97%, ^{n}Bu 2%	Sn 0%, C^C 100%, ^{n}Bu 0%	Sn -0.019

Theoretical Calculations of $[Au(C^\wedge C)(CN)_2]^-$ (3^-)

Table S10. Selected parameters of DFT optimized geometries for ground state and triplet state in gas phase.

Distance/angle	Single crystal	S_0	T_1
Au-C(CN)	2.062(8)<br>2.049(9)	2.07966<br>2.07968	2.07864<br>2.07834
Au-C(C^C)	2.044(9)<br>2.049(8)	2.07556<br>2.07541	2.05936<br>2.05686
C(C^C)-Au-C(C^C)	81.8(3)	80.485	80.834
C(C^C)-Au-C(CN)	94.6(3)<br>95.0(4)	92.709<br>92.749	92.627<br>92.586
C(CN)-Au-C(CN)	88.8(3)	94.056	93.953

Table S11. Selected frontier Molecular Orbitals and Composition (%) of Frontier MOs in terms of ligands and metals for ground state in gas phase.

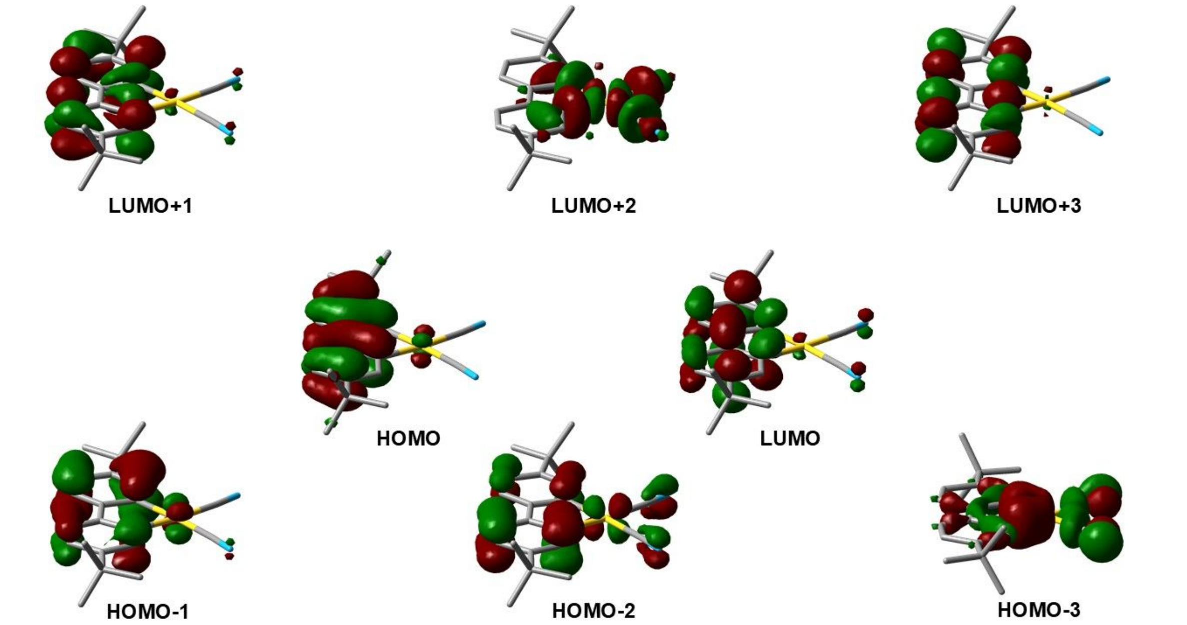

Orbital	Energy (eV)	Au	C^C	CN
LUMO+5	3.01	3	96	1
LUMO+4	2.59	90	9	0
LUMO+3	2.30	1	99	0
LUMO+2	1.85	36	43	21
LUMO+1	1.52	8	89	3
LUMO	1.03	10	86	4
HOMO	-2.46	2	97	0
HOMO-1	-3.32	5	94	1
HOMO-2	-3.40	10	80	10
HOMO-3	-3.50	8	50	42
HOMO-4	-3.53	3	38	59
HOMO-5	-4.07	12	19	69

Table S12. Selected vertical excitation energies singlets (S_n) and first triplet state (T_1) computed by TD-DFT (Gas Phase) with the orbitals involved.				
**State**	**λ/nm**	***f***	**Transition (% Contribution)**	**Character**
T_1	384	/	HOMO → LUMO (83%)	IL
S_1	289	0.2138	HOMO → LUMO (92%)	IL
S_2	265	0.0611	HOMO → L+2 (70%)<br>H-1 → LUMO (21%)	IL/LMCT/LL'CT
S_3	245	0.0001	HOMO → L+3 (68%)<br>H-3 → LUMO (13%)	IL/L'LCT
S_7	225	0.6626	H-1 → LUMO (67%)<br>HOMO → L+2 (26%)	IL/LMCT/LL'CT
S_{10}	205	0.1989	H-2 → L+2 (33%)<br>HOMO → L+5 (19%)<br>H-2 → LUMO (19%)<br>H-5 → LUMO (10%)	L'LCT/IL/LMCT/LL'CT

Table S13. Plots and composition (%) of the frontier MOs and spin density of the first triplet state in gas phase.		
**SOMO**	**SOMO-1**	**Spin Density**
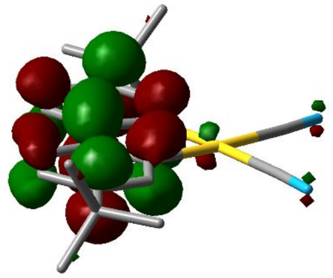	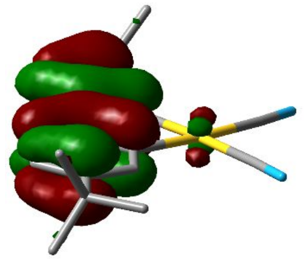	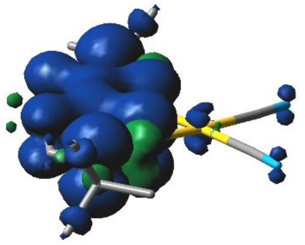
Au 5%; C^C 93%; CN 2%	Au 2%; C^C 98%; CN 0%	Au 0.0405

Table S14. Selected parameters of DFT optimized geometries for ground state and triplet state in CH_2Cl_2 solution.			
**Distance/angle**	**Single crystal**	**S_0**	**T_1**
Au-C(CN)	2.062(8)<br>2.049(9)	2.09071<br>2.09167	2.08779<br>2.08693
Au-C C(C^C)	2.044(9)<br>2.049(8)	2.07252<br>2.07281	2.05210<br>2.05445
C(C^C)-Au-C(C^C)	81.8(3)	80.587	80.813
C(C^C)-Au-C(CN)	94.6(3)<br>95.0(4)	93.596<br>93.755	93.694<br>93.517
C(CN)-Au-C(CN)	88.8(3)	92.062	91.977

Table S15. Selected frontier Molecular Orbitals and Composition (%) of Frontier MOs in terms of ligands and metals for ground state in CH_2Cl_2 solution.

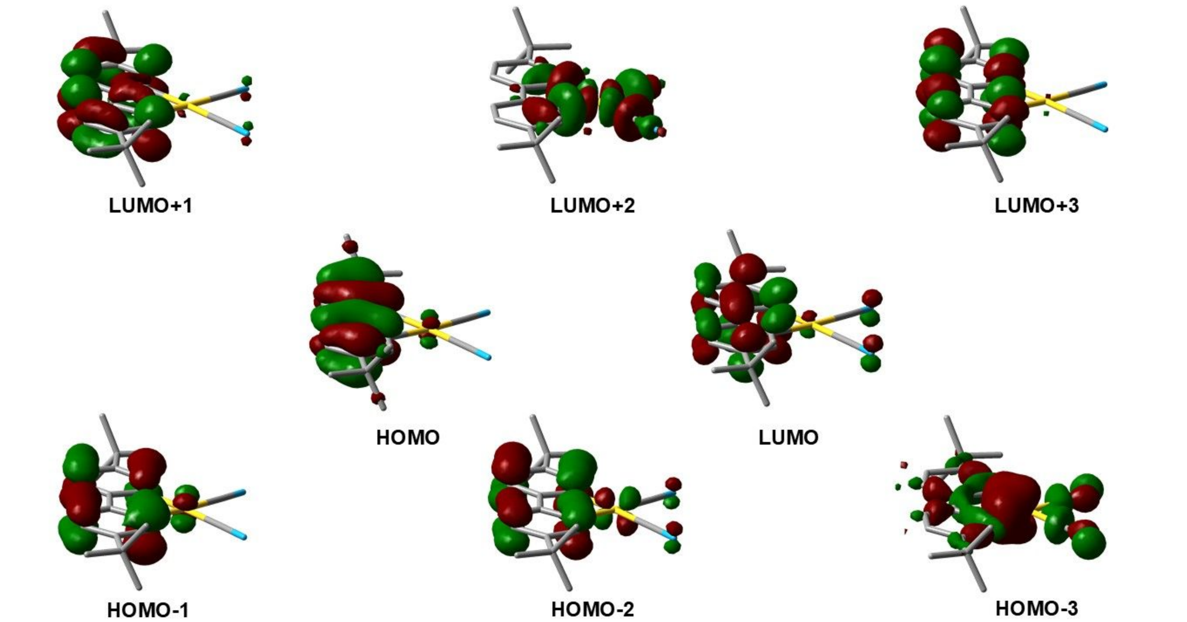

Orbital	Energy (eV)	Au	C^C	CN
LUMO+5	0.32	34	32	33
LUMO+4	0.3	99	2	0
LUMO+3	-0.15	1	99	1
LUMO+2	-0.93	36	45	19
LUMO+1	-0.97	7	90	3
LUMO	-1.5	15	77	8
HOMO	-4.91	2	98	0
HOMO-1	-5.81	4	95	0
HOMO-2	-5.96	8	88	4
HOMO-3	-6.28	10	66	24
HOMO-4	-6.41	5	47	48
HOMO-5	-6.52	1	99	0

Table S16. Selected vertical excitation energies singlets (S_n) and first triplet state (T_1) computed by TD-DFT (CH_2Cl_2 Solution) with the orbitals involved.

State	λ/nm	*f*	Transition (% Contribution)	Character
T_1	388	/	HOMO → LUMO (78%)	IL
S_1	297	0.2316	HOMO → LUMO (93%)	IL
S_2	266	0.1272	HOMO → L+1 (71%)<br>H-1 → LUMO (19%)	IL
S_5	231	0.8536	H-1 → LUMO (69%)<br>HOMO → L+1 (25%)	IL
S_9	211	0.1574	HOMO → L+4 (42%)<br>H-3 → L+3 (38%)	IL/LMCT/L'LCT

Table S17. Plots and composition (%) of the frontier MOs and spin density of the first triplet state in CH_2Cl_2 solution.

SOMO	SOMO-1	Spin Density
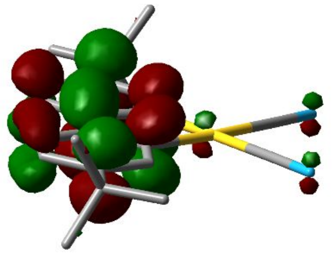	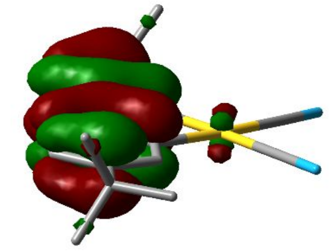	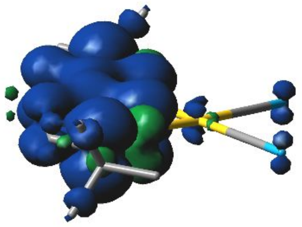
Au 7%; C^C 90%; CN 3%	Au 1%; C^C 99%; CN 0%	Au 0.062

Theoretical Calculations of [Pt(C^C)(COD)] (4)

Table S18. Selected parameters of DFT optimized geometries for ground state and triplet state in gas phase.

Distance/angle	Single Crystal	S_0	T_1
Pt-C(COD)	2.156 (cent 21-22)<br>2.136 (cent 25-26)	2.188<br>2.189	2.164<br>2.164
Pt- C(C^C)	2.035(3)<br>2.034(2)	2.046<br>2.046	2.009<br>2.009
C(C^C)-Pt-C(C^C)	81.18	80.57	80.59
C(C^C)-Pt-C(COD)	96.47<br>97.54	97.92<br>97.92	97.91<br>97.91
C(COD)-Pt-C(COD)	84.95	83.81	83.85

Table S19. Selected frontier Molecular Orbitals and Composition (%) of Frontier MOs in terms of ligands and metals for ground state in gas phase.

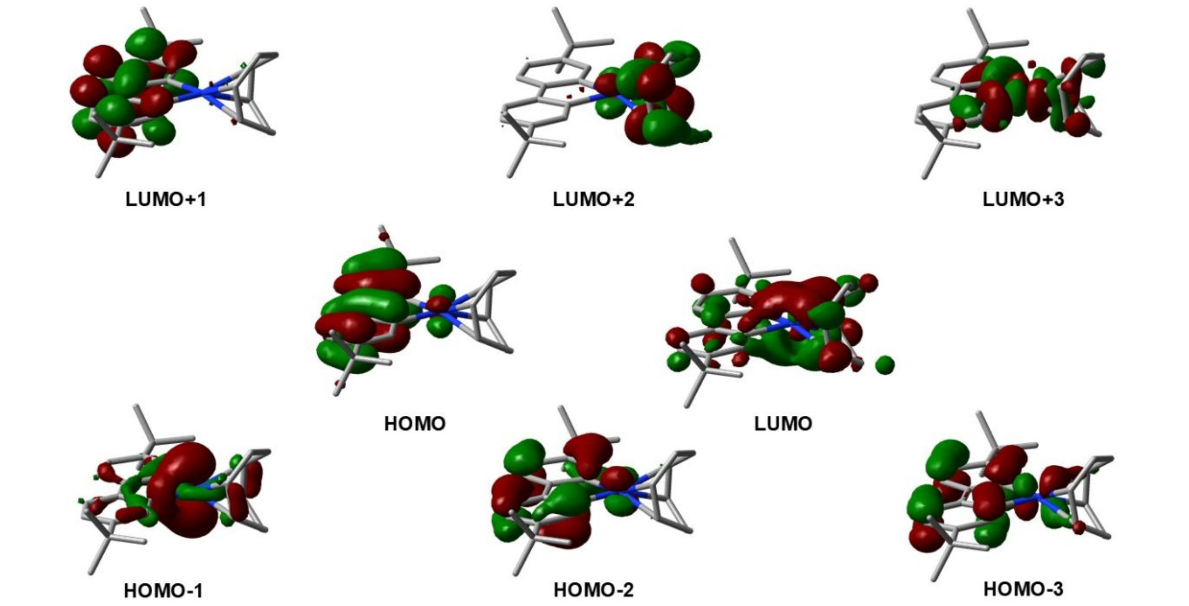

Orbital	Energy (eV)	Pt	C^C	COD
LUMO+5	-0.1	9	71	20
LUMO+4	-0.29	96	4	1
LUMO+3	-0.35	41	45	13
LUMO+2	-0.83	12	7	81
LUMO+1	-0.95	1	94	5
LUMO	-2.09	20	31	49
HOMO	-4.62	5	93	2
HOMO-1	-5.44	54	40	6
HOMO-2	-5.56	11	87	3
HOMO-3	-5.65	16	75	8
HOMO-4	-6.35	4	78	18
HOMO-5	-6.37	5	66	29

Table S20. Selected vertical excitation energies singlets (S_n) and first triplet state (T_1) computed by TD-DFT (Gas Phase) with the orbitals involved.

State	λ/nm	*f*	Transition (% Contribution)	Character
T_1	433	/	HOMO → LUMO (73%)<br>HOMO → L+2 (12%)	IL/LMCT/LL'CT
S_1	362	0.0033	HOMO → LUMO (93%)	IL/LMCT/LL'CT
S_2	305	0.0045	H-3 → LUMO (88%)	IL/LMCT/LL'CT
S_3	282	0.0263	HOMO → L+2 (46%)<br>H-1 → LUMO (39%)	MLCT/ML'CT/ LL'CT
S_4	267	0.1679	H-2 → LUMO (81%)	IL/LMCT/LL'CT
S_5	261	0.4592	HOMO → L+2 (31%)<br>H-1 → LUMO (29%)<br>H-3 → L+7 (12%)	MLCT/ML'CT/ LL'CT
S_9	241	0.4154	H-3 → L+7 (34%)<br>H-6 → L+7 (11%)<br>HOMO → L+2 (10%)	L'LCT/IL/LL'CT

Table S21. Plots and composition (%) of the frontier MOs and spin density of the first triplet state in gas phase.

SOMO	SOMO-1	Spin Density
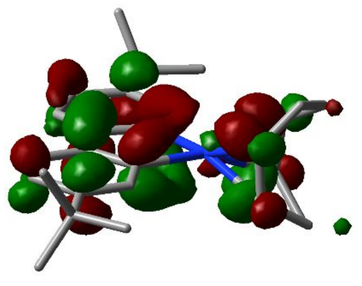	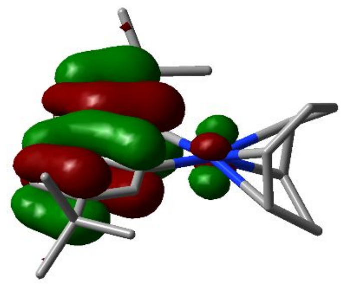	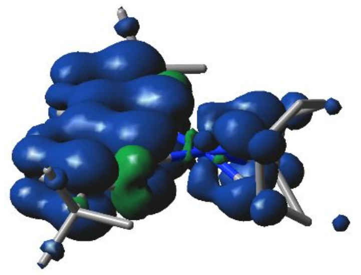
Pt 12%; C^C 61%;<br>COD 27%	Pt 5%; C^C 93%;<br>COD 2%	Pt 0.1222

Table S22. Selected parameters of DFT optimized geometries for ground state and triplet state in CH_2Cl_2 solution.

Distance/angle	Single Crystal	S_0	T_1
Pt-C(COD)	2.156 (cent 21-22)<br>2.136 (cent 25-26)	2.200	2.175
Pt- C(C^C)	2.035(3)<br>2.034(2)	2.047	2.010
C(C^C)-Pt-C(C^C)	81.18	80.54	80.60
C(C^C)-Pt-C(COD)	96.47<br>97.54	98.12	98.08
C(COD)-Pt-C(COD)	84.95	83.47	83.53

Table S23. Selected frontier Molecular Orbitals and Composition (%) of Frontier MOs in terms of ligands and metals for ground state in CH_2Cl_2 solution.

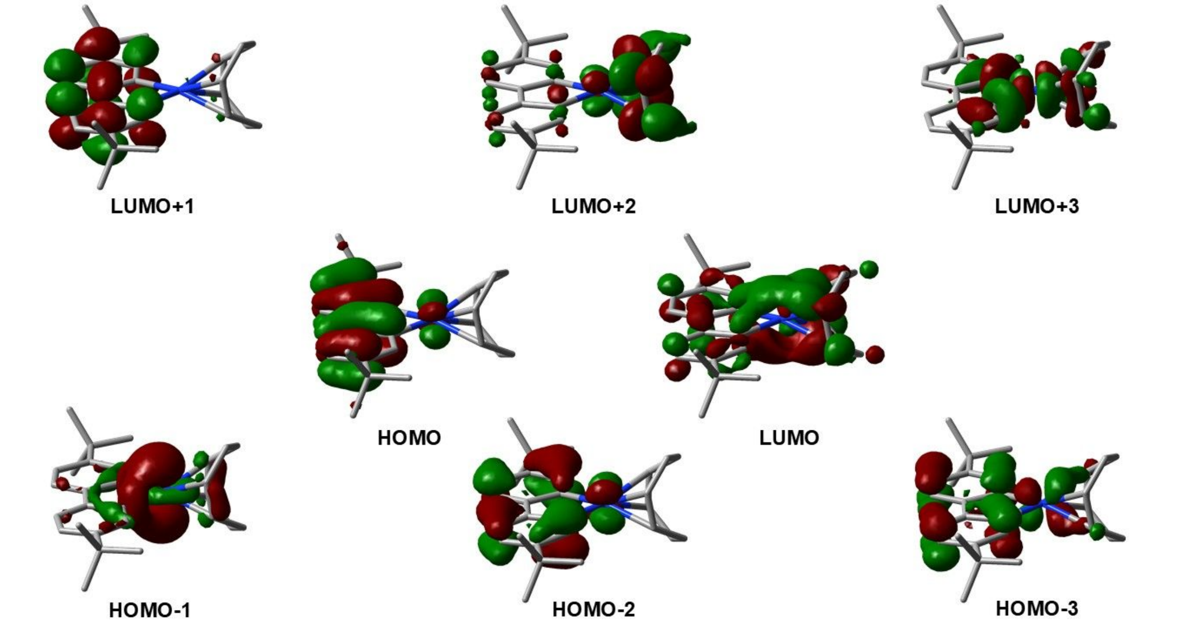

Orbital	Energy (eV)	Pt	C^C	COD
LUMO+5	-0.22	78	19	3
LUMO+4	-0.26	10	65	25
LUMO+3	-0.46	42	44	14
LUMO+2	-0.76	9	16	75
LUMO+1	-1.2	1	93	5
LUMO	-2.14	19	39	43
HOMO	-4.86	7	91	2
HOMO-1	-5.49	61	33	6
HOMO-2	-5.79	14	83	3
HOMO-3	-5.83	23	67	9
HOMO-4	-6.4	6	42	52
HOMO-5	-6.42	80	15	5

Table S24. Selected vertical excitation energies singlets (S_n) and first triplet state (T_1) computed by TD-DFT (CH_2Cl_2 Solution) with the orbitals involved.

State	λ/nm	*f*	Transition (% Contribution)	Character
T_1	421	/	HOMO → LUMO (74%)<br>HOMO → L+2 (12%)	LMCT/LL'CT/IL
S_1	346	0.0107	HOMO → LUMO (92%)	LMCT/LL'CT/IL
S_2	303	0.0093	H-1 → LUMO (86%)	IL/ML'CT
S_3	280	0.1039	HOMO → L+2 (62%)<br>H-2 → LUMO (25%)	LMCT/LL'CT/IL
S_4	265	0.2664	H-3 → LUMO (84%)	IL/LL'CT/ML'CT
S_5	260	0.5348	H-2 → LUMO (39%)<br>HOMO → L+2 (19%)<br>H-1→ L+7 (12%)	IL/LMCT/LL'CT/MLCT
S_6	248	0.466	H-1 → L+7 (23%)<br>HOMO → L+7 (13%)<br>H-6 → L+7 (10%)<br>H-2→ LUMO (10%)	LMCT/LL'CT/IL/MLCT/L'LCT
S_8	244	0.102	HOMO → L+7 (20%)	IL

Table S25. Plots and composition (%) of the frontier MOs and spin density of the first triplet state in CH_2Cl_2 solution.

SOMO	SOMO-1	Spin Density
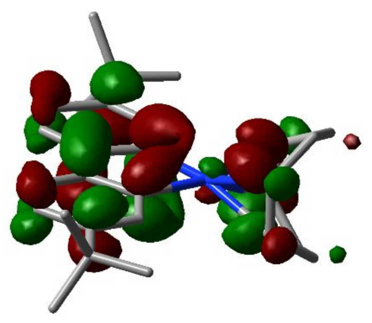	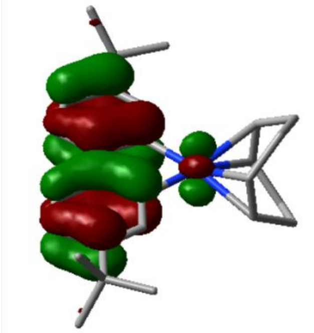	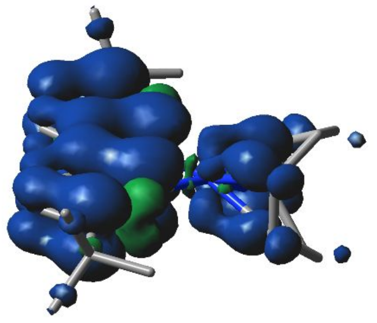
Pt 12%; C^C 63%;<br>COD 25%	Pt 5%; C^C 93%;<br>COD 2%	Pt 0.1297

Theoretical Calculations of $[Pt(C\^C)(CN)_2]^{2-}$ (5^{2-})

Table S26. Selected parameters of DFT optimized geometries for ground state and triplet state in gas phase.

Distance/angle	Single Crystal	S_0	T_1
Pt-C(CN)	/	2.05213<br>2.05206	2.07228<br>2.07357
Pt- C(C^C)	/	2.05669<br>2.05722	2.03022<br>2.02512
C(C^C)-Pt-C(C^C)	/	80.290	81.663
C(C^C)-Pt-C(CN)	/	92.393<br>92.248	92.611<br>92.527
C(CN)-Pt-C(CN)	/	95.069	93.199

Table S27. Selected frontier Molecular Orbitals and Composition (%) of Frontier MOs in terms of ligands and metals for ground state in gas phase.

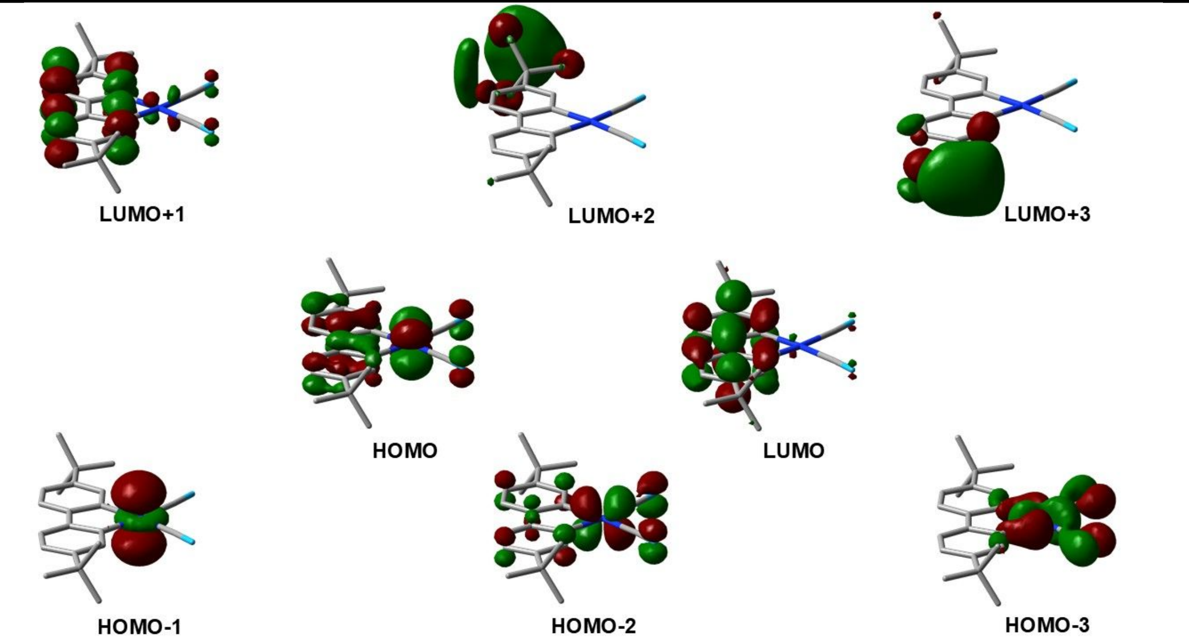

Orbital	Energy (eV)	Pt	C^C	CN
LUMO+5	6.4	0	100	0
LUMO+4	5.83	2	97	1
LUMO+3	5.68	2	98	0
LUMO+2	5.61	5	95	0
LUMO+1	5.11	11	85	4
LUMO	4.62	5	94	2
HOMO	1.79	53	38	10
HOMO-1	1.66	98	4	-1
HOMO-2	1.47	64	22	14
HOMO-3	1.33	53	11	36
HOMO-4	0.75	15	77	9
HOMO-5	0.7	-2	57	46

Table S28. Selected vertical excitation energies singlets (S_n) and first triplet state (T_1) computed by TD-DFT (Gas Phase) with the orbitals involved.				
**State**	**λ/nm**	***f***	**Transition (% Contribution)**	**Character**
T_1	401	/	HOMO → LUMO (81%)	MLCT
S_1	325	0,0622	HOMO → LUMO (90%)	MLCT
S_2	293	0,019	H-1 → LUMO (87%)	MLCT
S_3	279	0,2073	HOMO → L+3 (83%)	MLCT
S_4	278	0,1154	H-2 → LUMO (82%)	MLCT
S_9	246	0,5619	H-4 → LUMO (78%)	IL

Table S29. Plots and composition (%) of the frontier MOs and spin density of the first triplet state in gas phase.		
**SOMO**	**SOMO-1**	**Spin Density**
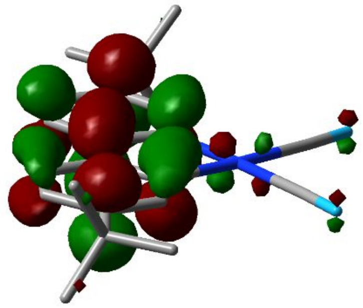	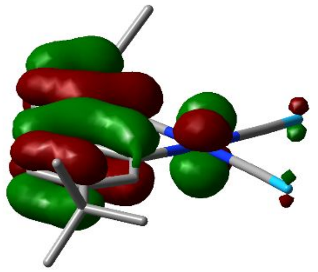	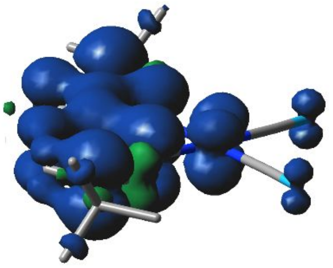
CN 2%, C^C 92% Pt 6%	CN 2%, C^C 78% Pt 19%	Pt 0.2378

Table S30. Selected parameters of DFT optimized geometries for ground state and triplet state in CH_2Cl_2 solution.			
**Distance/angle**	**Single Crystal**	**S_0**	**T_1**
Pt-C(CN)	/	2.04659<br>2.04692	2.06280<br>2.06176
Pt- C(C^C)	/	2.06434<br>2.06378	2.03360<br>2.02863
C(C^C)-Pt-C(C^C)	/	80.437	81.458
C(C^C)-Pt-C(CN)	/	93.113<br>93.183	93.135<br>93.324
C(CN)-Pt-C(CN)	/	93.278	92.094

Table S31. Selected frontier Molecular Orbitals and Composition (%) of Frontier MOs in terms of ligands and metals for ground state in CH_2Cl_2 solution

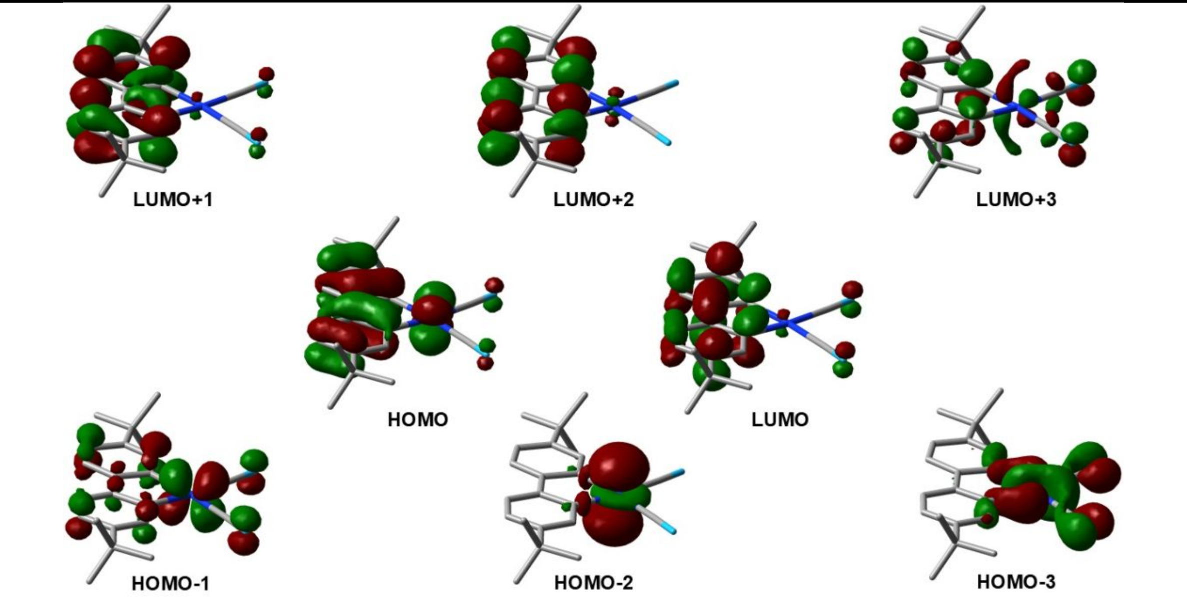

Orbital	Energy (eV)	Pt	C^C	CN
LUMO+5	1.49	44	54	2
LUMO+4	1.43	1	99	0
LUMO+3	1.3	52	33	15
LUMO+2	0.76	1	97	1
LUMO+1	-0.11	10	85	5
LUMO	-0.56	14	79	7
HOMO	-3.69	30	66	4
HOMO-1	-4.17	56	33	11
HOMO-2	-4.18	94	8	-1
HOMO-3	-4.48	54	15	31
HOMO-4	-4.5	26	68	6
HOMO-5	-4.93	0	65	35

Table S32. Selected vertical excitation energies singlets (S_n) and first triplet state (T_1) computed by TD-DFT (CH_2Cl_2 solution) with the orbitals involved.				
**State**	**λ/nm**	***f***	**Transition (% Contribution)**	**Character**
T_1	398	/	HOMO → LUMO (82%)	IL/MLCT
S_1	319	0,0701	HOMO → LUMO (91%)	IL/MLCT
S_2	277	0,2355	HOMO → L+1 (83%)	IL/MLCT
S_3	268	0,208	H-1 → LUMO (85%)	MLCT
S_5	250	0,8287	H-3 → LUMO (67%)	MLCT/L'LCT

Table S33. Plots and composition (%) of the frontier MOs and spin density of the first triplet state in CH_2Cl_2 solution.		
**SOMO**	**SOMO-1**	**Spin Density**
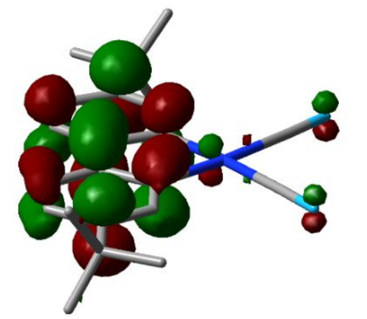	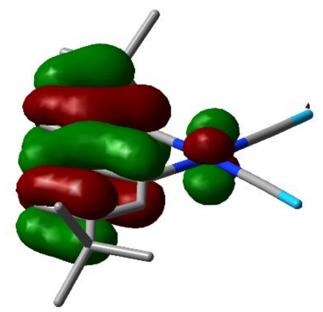	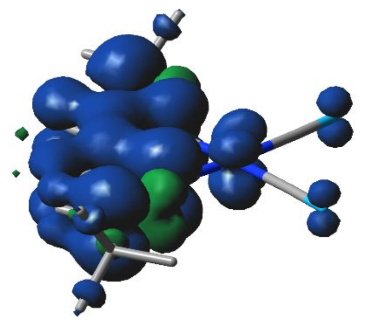
CN 4%, C^C 87%, Pt 9%	CN 1%, C^C 88%, Pt 11%	Pt 0.1810

Table S34. Calculated Emissions of Sn($^tBu_2Bip$)$^nBu_2$ (2), [Au($^tBu_2Bip$)$(CN)_2$]$^-$ (3^-), [Pt($^tBu_2Bip$)(COD)] (4) and [Pt($^tBu_2Bip$)$(CN)_2$]$^{2-}$ (5^{2-}) in different media.		
**Complex**	**CH_2Cl_2 solutions**	**Gas Phase**
**Sn($^tBu_2Bip$)$^nBu_2$ (2)**	532 nm	534 nm
**[Au($^tBu_2Bip$)$(CN)_2$]$^-$ (3^-)**	564 nm	555 nm
**[Pt($^tBu_2Bip$)(COD)] (4)**	659 nm	684 nm
**[Pt($^tBu_2Bip$)$(CN)_2$]$^{2-}$ (5^{2-})**	584 nm	588 nm

S5. Singlet Oxygen Measurements

The singlet oxygen quantum yield measurements were performed by monitoring the phosphorescence of $^1O_2$ generated by **3**, **5** and by the compound used as reference (phenalenone), at 1274 nm employing an Ediumburgh FLS1000 spectrofluorimeter fitted with a Xe lamp and a NIR detector (900 – 1800 nm) while absorbance measurements were performed with a Hewlett Packard 8453 spectrophotometer. For the measurements, 5×10^{-5} M solutions in acetonitrile of **3**, **5** and PN (phenalenone) were used. The emission spectra of **3** were recorded at 348 nm, for **5** the spectra were recorded at 334 and 342 nm and for PN the spectra were recorded at 334, 342 and 348 nm. The area below the band was integrated with Origin Pro2018. The corresponding singlet oxygen quantum yield, Φ ($^1O_2$), was calculated by referencing it to the literature value reported for phenalenone (Φ = 1) in acetonitrile, according to the following equation:[S14]

$$\phi_C = \frac{I_C/A_C}{I_R/A_R} \text{ x } \phi_R \qquad \text{(Equation 2)}$$

In this expression, ϕ_C represents the $^1O_2$ quantum yield of the compound and ϕ_R corresponds to that of the reference; A_C and A_R are the absorbances of the compounds and the reference, respectively, while I_C and I_R denote the integrated areas of the singlet oxygen emission of the compound and the reference.

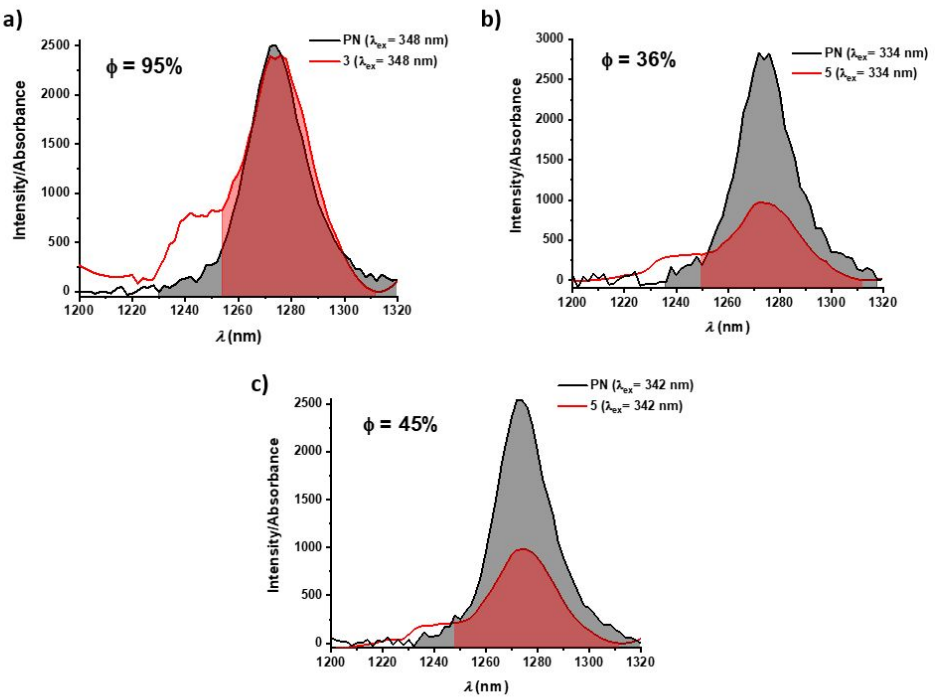

Figure S27. Monitored $^1O_2$ phosphorescence emission of (a) PN and **3** at 348 nm (b) PN and **5** at 334 nm and (c) PN and **5** at 342 nm.

S6. Electrochemical Properties

Cyclic voltammetry (CV) and differential pulse voltammetry (DPV) experiments have been carried out at 298 K in CH_2Cl_2 (5×10^{-4} M) solutions using a 0.1 M solution of $(NBu_4)PF_6$ as support electrolyte and under strict anhydrous and inert atmosphere conditions. All measurements were carried out at 100 mV s^{-1} *vs.* Ag/AgCl reference electrode.

The following formula was used to calculate E_{HOMO} and E_{LUMO} from VC. In this formula the potential of ferrocene *vs.* Ag/AgCl (E^{Fc/Fc^+}) is 0.45 V.

$$E_{HOMO/LUMO} = -(E^{ox/red} + 4.8 - E^{Fc/Fc^+}) \qquad \text{(Equation 1)}$$

A summary of the data is collected in Table S35.

Table S35. Electrochemical data, HOMO energy estimated through electrochemical data and calculated by TD-DFT

Complex	Cyclic Voltammetry				DFT Calculations	
	E^{ox}/V	E^{red}/V	E_{HOMO}/eV	E_{LUMO}/eV	E_{HOMO}/eV	E_{LUMO}/eV
**1**	1.425		-5.775		-5.28	-1.59
**3**	1.280		-5.648		-4.91	-1.5
**5**	0.522<br>0.956<br>1.266		-4.872<br>-5.306<br>-5.616		-4.86	-2.14

S7. Photocatalysis

Stock solutions of K[Au(C^C)$(CN)_2$] **3^K** (6.25 × 10^{-3} M and 6.25 × 10^{-4} M), (NBu_4)[Au(C^C)$(CN)_2$] **3** (6.25 × 10^{-4} M), $(NBu_4)_2$[Pt(C^C)$(CN)_2$] **5** (6.25 × 10^{-4} M), p-bromothioanisole (0.20 M), DABCO (0.075 M) and BQ (0.075 M) were prepared. The photocatalytic reactions were carried out by mixing 400 µL of the diluted solution of KAuCN (for 1% catalysis) or 200 µL of the concentrated solution (for 5% catalysis), 125 µL of p-bromothioanisole solution and with 50 µL of BQ or DABCO solution when they were neccesary. The reactions were made in 600 µL of CD_3OD or CD_3OD/D_2O (1:1) mix irradiating with UV light (λ = 350 nm).

Table S36. Homogeneous UV-Light Oxidative Reactions with Different Conditions

Entry	Catalyst (%)	Light	Atmosphere	Time	% Conversion
1	5 (5)	Yes	O_2	3h	8
2	3 (5)	Yes	O_2	3h	82
3	3^K (1)	Yes	O_2	6h	95
4	3^K (5)	Yes	O_2	2.5h	97
5	3^K (0)	Yes	O_2	2.5h	0
6	3^K (1)	No	O_2	6h	0
7	3^K (5)	Yes	N_2	2.5h	5
8[a]	3^K (5)	Yes	O_2	2.5h	4
9[b]	3^K (5)	Yes	O_2	2.5h	15

[a] DABCO, [b] BQ

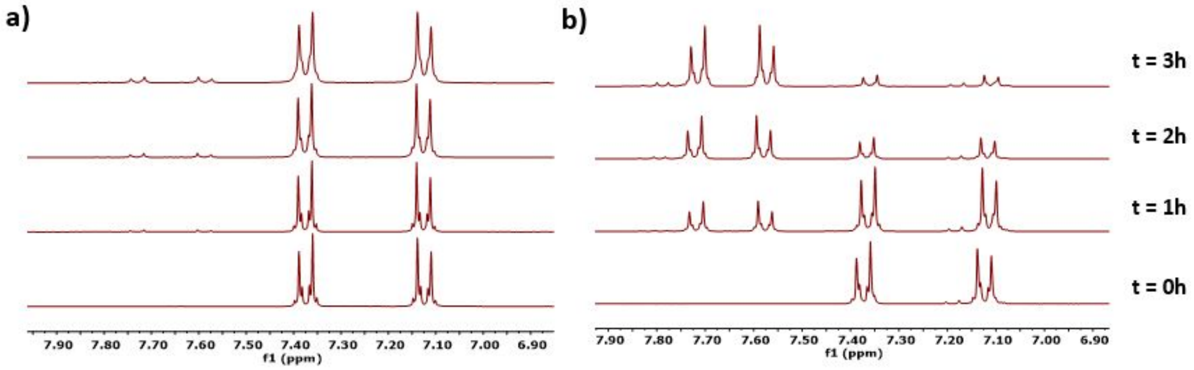

Figure S28. Monitored evolution of homogeneous UV-light oxidative reactions with 5% catalyst (a) **5** or (b) **3**.

Table S37. Monitoring the generation of the oxidized product using different catalysts at 5%		
**Catalyst**	**Time (h)**	**Generate Product (%)**
**5**	0	0
	1	4
	2	6
	3	8
**3**	0	0
	1	30
	2	64
	3	82

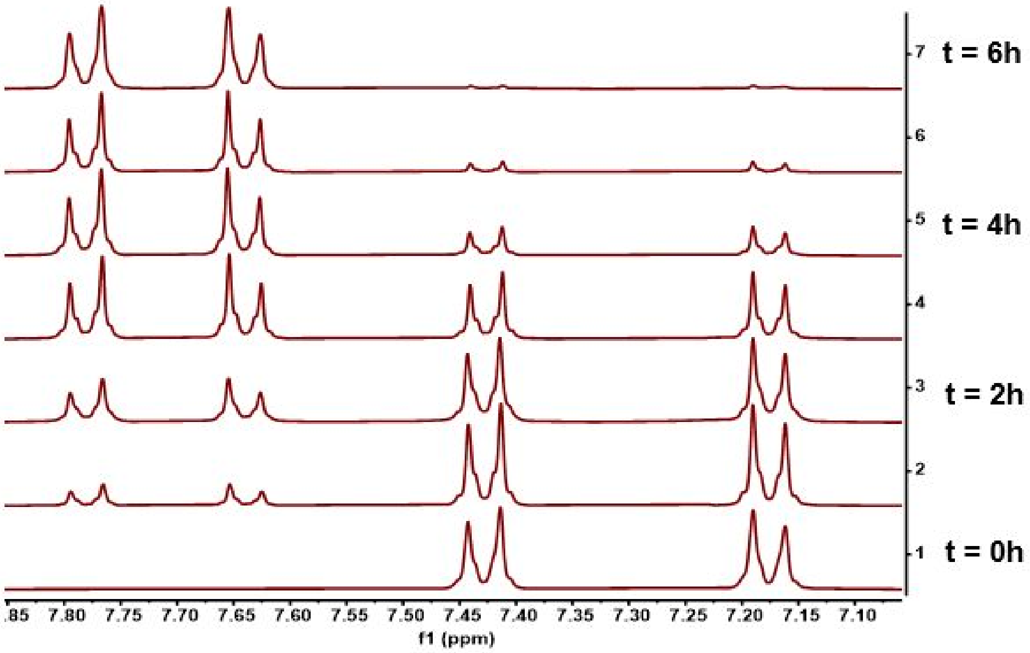

Figure S29. Monitored evolution of homogeneous UV-light oxidative reactions with 1% of catalyst $\mathbf{3^{K}}$.

Table S38. Monitoring the generation of the oxidized product with 1% of catalyst 3^{K}	
**Time (h)**	**Generate Product (%)**
1	16
2	32
3	54
4	74
5	87
6	95

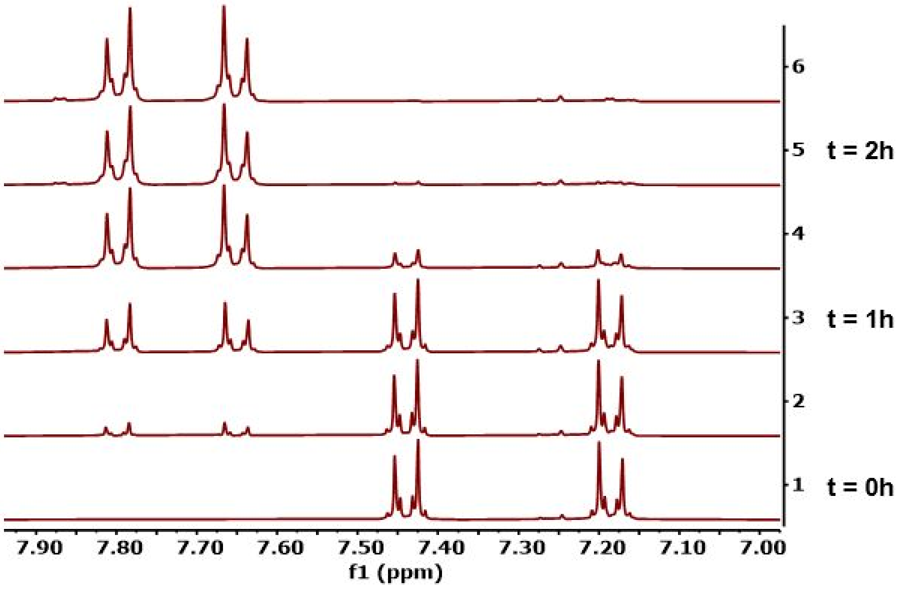

Figure S30. Monitored evolution of homogeneous UV-light oxidative reactions with 5% of catalyst $\mathbf{3^K}$.

Table S39. Monitoring the generation of the oxidized product with 5% of catalyst 3^K	
**Time (min)**	**Generate Product (%)**
30	14
60	38
90	79
120	94
150	97

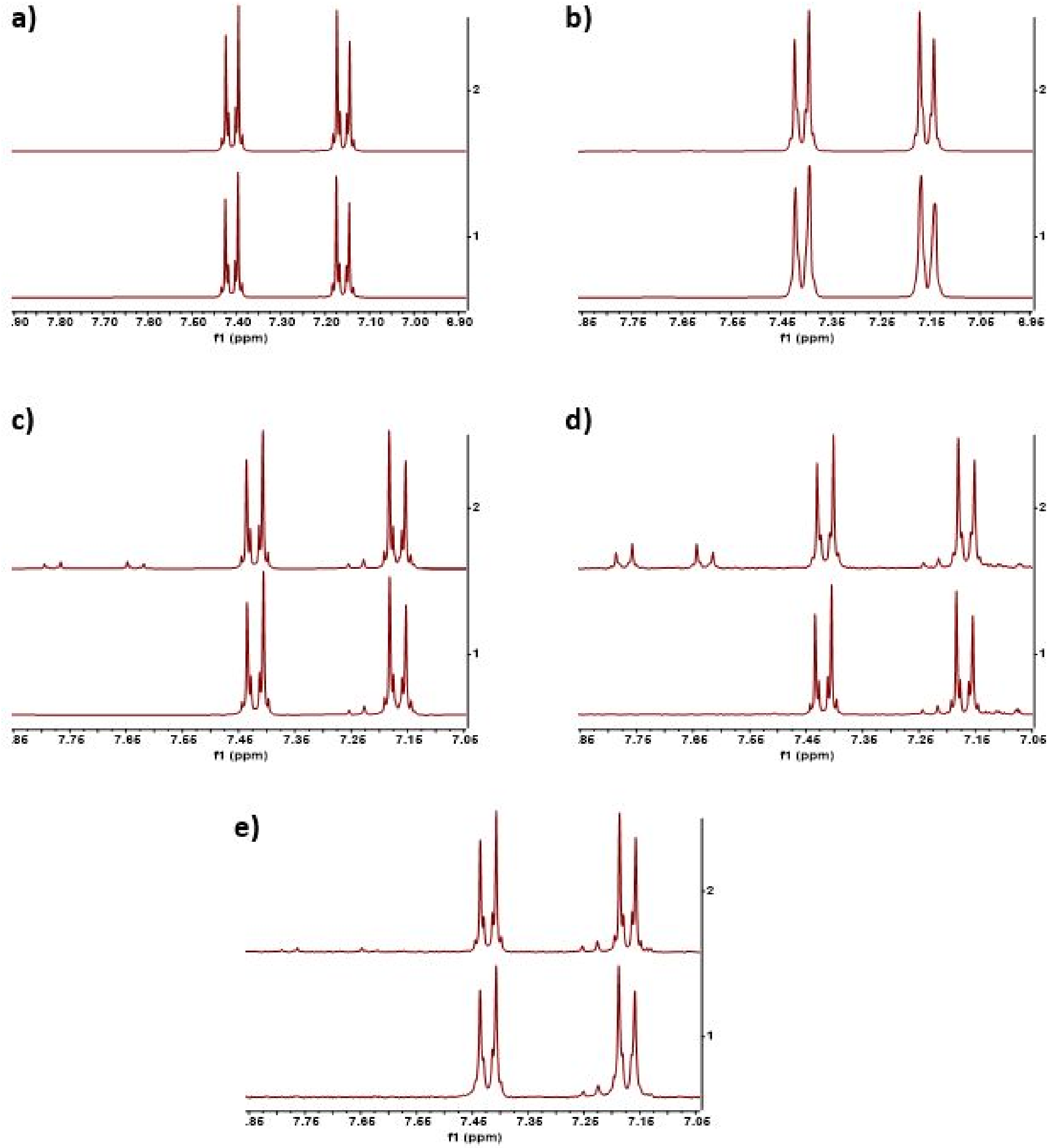

Figure S31. Monitored evolution of homogeneous UV-light oxidative reactions without (a) light, (b) catalyst, (c) oxygen, (d) with DABCO and (e) with BQ.

References.

S1. SAINT, SADABS, Bruker Analytical X-ray Systems, Madison, WI, **2012**.

S2. CrysAlisRED, CCD camera data reduction program. Rigaku Oxford Diffraction, Oxford, UK, **2019**

S3. Bourhis, L. J.; Dolomanov, O. V.; Gildea, R. J.; Howard, J. A. K.; Puschmann, H. "The Anatomy of a Comprehensive Constrained, Restrained, Refinement Program for the Modern Computing Environment - Olex2 Disected" *Acta Cryst.,* **2015**, *A71*, 59-71.

S4. Sheldrick G. M. "Crystal structure refinement with SHELXL" Acta Crystallogr., **2015**, *C71*, 3-8.

S5. Frisch, M. J., Trucks, G. W., Schlegel, H. B., Scuseria, G. E., Robb, M. A., Cheeseman, J. R., Scalmani, G., Barone, V., Petersson, G. A., Nakatsuji, H., et al., Revision A.03, Inc., Gaussian 16, Wallingford CT, 2016, 2016.

S6. (a) Becke, A. D., J. Chem. Phys., 1993, 98, 5648-5652. (b) Becke, A. D., Phys. Rev. A, 1988, 38, 3098-3100.)

S7. Grimme, S., Antony, J., Ehrlich, S., Krieg, H., J. Chem. Phys., 2010, 132, 154104.

S8. Y. Zhao, D. G. Truhlar, J. Chem. Phys. 2006, 125, 194101

S9. Y. Zhao, D. G. Truhlar, Theor. Chem. Acc. 2008, 120, 215– 241.

S10. D. Andrae, U. H-ussermann, M. Dolg, H. Stoll, H. Preuss, Theor. Chim. Acta 1990, 77, 123 – 141.

S11. (a) R. Ditchfield, W. J. Hehre, J. A. Pople, J. Chem. Phys. 1971, 54, 724 – 728; b) W. J. Hehre, R. Ditchfie, J. A. Pople, J. Chem. Phys. 1972, 56, 2257 – 2261; c) P. C. Hariharan, J. A. Pople, Theor. Chim. Acta 1973, 28, 213– 222; d) M. M. Francl, W. J. Pietro, W. J. Hehre, J. Chem. Phys. 1982, 77, 3654.).

S12. (a) Barone, V., Cossi, M., J. Phys. Chem. A, 1998, 102, 1995-2001. (b) S. Miertusˇ, E. Scrocco, J. Tomasi, Chem. Phys. 1981, 55, 117– 129.

S13. O'Boyle, N. M., Tenderholt, A. L., Langner, K. M., J. Comput. Chem., 2008, 29, 839-845.

S14. Schweitzer, Claude, y Reinhard Schmidt. «Physical Mechanisms of Generation and Deactivation of Singlet Oxygen». *Chemical Reviews* 103, n.º 5 (2003): 1685-758.
